# Nonclinical characterization of ICVB-1042 as a selective oncolytic adenovirus for solid tumor treatment

**DOI:** 10.1038/s42003-024-06839-6

**Published:** 2024-09-13

**Authors:** Yu Kato, Nathaniel Rice, Michael Pokrass, Jinkil Jeong, Ruben Rodriguez, Jessica J. Field, Heba Nowyhed

**Affiliations:** IconOVir Bio, New York, NY USA

**Keywords:** Tumour virus infections, Cancer immunotherapy

## Abstract

ICVB-1042 is an oncolytic adenovirus containing modifications to enhance replication, lysis, and viral spreading in tumor cells. The anti-tumor activity, immune activation, tropism, selectivity, and mechanism of action were evaluated in preparation for a first-in-human study. ICVB-1042 was at least 100-fold more cytotoxic in A549 cells than in normal primary cells tested, demonstrating its high tumor selectivity and a low likelihood of targeting primary tissues. ICVB-1042 administered to mice intravenously or intratumorally was effective in reducing tumor burden. Its intravenous administration also inhibited tumor growth in orthotopic models. ICVB-1042 was well tolerated in mice compared to HAdV-C5 (Wt Ad5), with reduced liver sequestration, supporting safety of the drug for systemic delivery. These preclinical data demonstrating the safety and potency of ICVB-1042 for treatment of various solid tumors support the ongoing clinical investigation (NCT05904236).

## Abstract (web summary)

ICVB-1042, an oncolytic adenovirus containing modifications to enhance replication, lysis, and viral spreading in tumor cells, demonstrated safety and potency in multiple tumor cell lines and mouse models of solid tumors.

## Introduction

Oncolytic viruses (OVs) are an emerging class of cancer therapeutics that harness the ability of certain viruses to selectively replicate within cancer cells, leading to their lysis and subsequent viral dissemination within the tumor microenvironment, while sparing normal tissue^1–3^. Adenoviruses (Ads), specifically adenovirus serotype 5 (Ad5, HAdv-C5), offer several advantages for clinical applications. Ad5 exhibits potent tumor lytic activities and can induce immunogenic cell death, leading to tumor inflammation and anti-tumor immune responses^4^. Ad genomes do not integrate into host DNA and can be produced to high titers using established protocols^5,6^. With established safety and manufacturing profiles, Ad5 has been extensively studied for gene therapy and oncolytic viral applications^3,7^. Despite potential advantages, the clinical application of Ad OVs remains restricted by factors such as limited tropism, restricted viral replication, hepatotoxicity, tolerability issues, and insufficient immune activation. ICVB-1042 is a replication-competent Ad5-based OV that has been rationally designed to overcome the limitations of other Ad OVs. Numerous modifications have been incorporated to enhance virus replication, lysis, and spreading in tumor cells. Notably, ICVB-1042 also encodes the yellow fluorescent protein variant YPet^8^ coupled to viral life-cycle, enabling detailed characterization of viral replication not previously possible with other OVs.

ICVB-1042 has engineered chimeric fibers composed of the knob from Ad serotype 34 (Ad34) and the shaft from Ad5. The rationale for using Ad34 was to introduce novelty while leveraging existing knowledge, as it differs from Ad35 by a single amino acid. Many Ad5-based OVs have faced challenges related to variable expression levels of their primary cell entry receptor, coxsackievirus and Ad receptor (CAR), on cancer cells. Since high levels of CAR are needed for efficient entry of Ad5, the variability in CAR expression among different cancer types and even within tumors contributes to resistance to Ad5-based OVs^9,10^. Ad5-based OVs such as Delta-24-RGD incorporate in the fiber knob an RGD motif to target integrins^11,12^. While this strategy increases binding selectivity for certain cancer types with high levels of integrin, it also suffers from variability in its expression levels^13^. The Ad5/Ad34 chimeric fiber of ICVB-1042 is designed to enable cell entry via human CD46 (huCD46), a surface protein widely expressed in tumor cells^14^.

Systemic administration of Ad OVs, while advantageous for achieving optimal tumor coverage compared to intratumoral (IT) administration, presents challenges due to the tendency of Ads to target hepatocytes with the associated hepatotoxicity^15,16^. To improve tolerability and tumor-targeting upon IV administration, the hexon of ICVB-1042 has been modified from that of wild-type (Wt) Ad5 to minimize binding to blood clotting factor X (FX) and reduce liver sequestration^17,18^. Furthermore, genetic modification of the OV achieving selective tumor cell killing while sparing healthy cells becomes particularly crucial for the success of systemic administration, but this is challenging without sacrificing the potency of the virus. Consequently, IT administration has been the predominant approach for evaluating Ad OVs, with limited and unsuccessful clinical trials exploring intravenous (IV) applications^19–21^. ICVB-1042 has been engineered to be tumor-selective via disruption of its ability to induce E2 transcription factor (E2F) activity in infected cells, thereby exploiting dysregulated E2F activity observed in almost all cancer types but not in normal cells^22,23^. The Wt Ad5 encodes adenoviral early region (E)1A protein and adenoviral control protein E4orf6/7 (E4orf6/7), two proteins that circumvent the requirement for cyclin-dependent kinase (CDK) signaling to activate E2F. E1A and E4orf6/7 can both bind Rb to liberate E2F independently of Rb phosphorylation, and E4orf6/7 has a separate function to bind E2F and dimerization partner (DP) heterodimer (E2F-DP) complexes to enhance their activity at target promoters in both the host to induce the cell to enter S-phase, and in viral genomes to promote viral replication^22,24^. ICVB-1042 encodes a mutant E1A protein with the LxCxE motif deleted (E1A^Δ15bp^), preventing E1A-mediated E2F liberation from Rb^25^. The D15 mutation present in ICVB-1042 deletes 5 amino acids (15 base pairs) from E1A as a precise mutation to abrogate pRb binding. Additionally, ICVB-1042 incorporates the deletion of E4orf6/7 to further enhance its tumor selectivity. These dual modifications provide more exquisite tumor selectivity by preventing the induction of S-phase in normal cells.

Further modifications in the ICVB-1042 genome include the deletion of 12.5K, 6.7K, 19K, RID-alpha, and 14.7K genes in the E3 region. However, the adenovirus death protein (ADP; E3-11.6K), critical for efficient cell lysis and release of the viral progeny^26^, is retained as YPet-P2A-ADP fusion with YPet. Deletion of these E3 genes has been shown to increase the expression of the ADP gene, which accelerates the cycle of virus-induced cell lysis and virus spreading^26^. Some of the deleted E3 genes are also involved in host immune evasion^27–32^. While Ad5 is generally considered immunogenic and, in some cases, Ad5-based OVs have been reported to induce cancer cell death in a manner characteristic of immunogenic cell death^33^, the deletion of the E3 genes is also expected to support adaptive immune responses targeting tumors. Since YPet is fused to ADP without additional regulatory elements in ICVB-1042, YPet expression is expected to parallel that of ADP, occurring during late lytic infection stages. While not a direct measure of cytolytic activity, the YPet reporter enables measurements of ICVB-1042 viral replication during lytic infection. The addition of the YPet fused to ADP constitutes a reporter system that will be expressed only when ICVB-1042 is replicating, creating a powerful translational tool.

Here, we conducted extensive preclinical investigations to evaluate the potency, selectivity, immunogenicity, and tolerability of ICVB-1042. ICVB-1042 efficiently entered cells via huCD46 and killed tumor cells. ICVB-1042 showed potent anti-tumor activity across a broad panel of cancer cell lines and primary dissociated tumor cells. Notably, the dual modifications in the E1A and E4orf/7 found in ICVB-1042 resulted in exceptional tumor selectivity, relying on the host cell cycle and E2F activity for replication. ICVB-1042 demonstrated significantly enhanced tolerability compared to Wt Ad5 in vivo, showed good safety and toxicity profiles at high doses in immune-competent mice, and achieved effective tumor control in bladder and breast human xenograft cancer models when administered IV or IT. Pharmacokinetic studies examining ICVB-1042 viral copy numbers in the circulation and tissues showed clear evidence of ICVB-1042 viral replication in tumors. Furthermore, ICVB-1042 induced notable immune activation in vitro and in vivo indicative of immunogenic cell death. The presented preclinical studies underscore the rational engineering of ICVB-1042, representing considerable advancements in safety and performance. These promising attributes warrant further investigation through systemic administration in solid tumor patients in the ongoing Phase 1 clinical trial (NCT05904236).

## Results

**Fig. 1:**
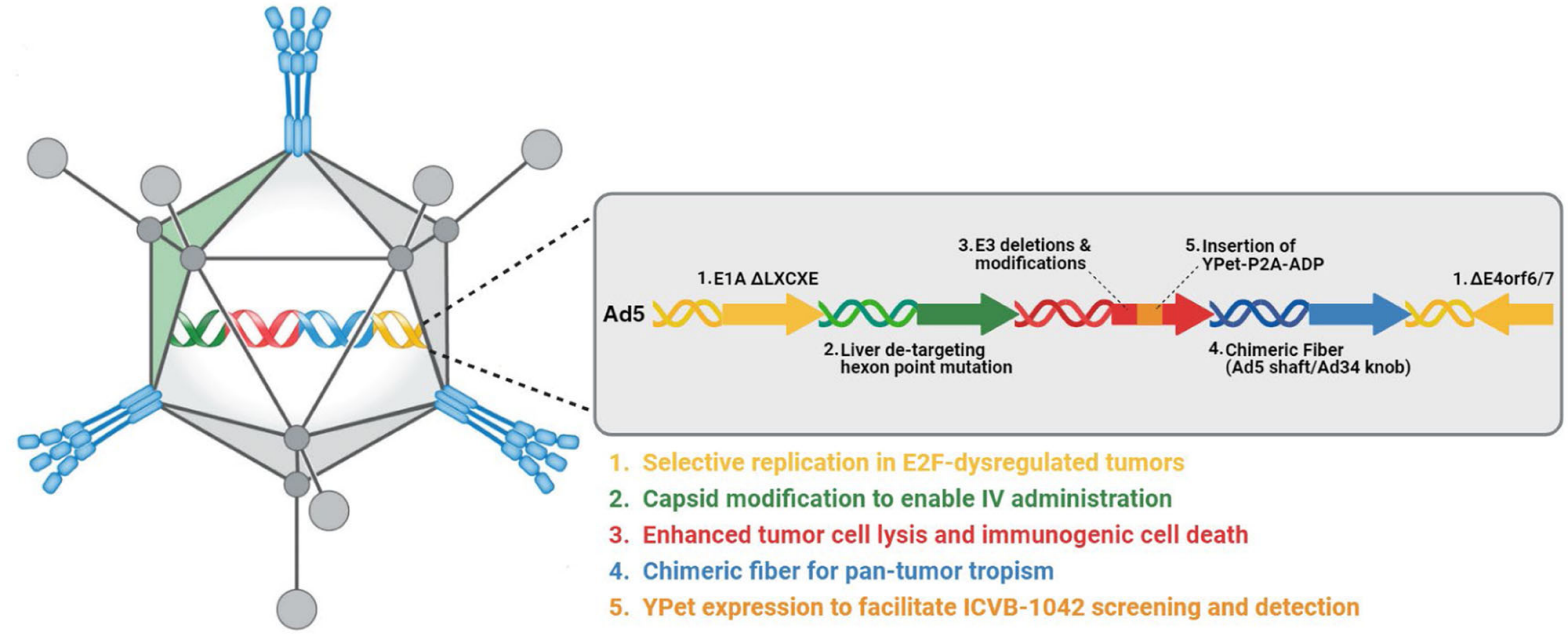
ICVB-1042 is an E2F-dependent oncolytic adenovirus composed of an Ad5 backbone with genomic modifications. 1. ΔLXCXE = mutation preventing binding of E1A to retinoblastoma (pRb) restricting virus to cells with high E2F activity. 2. Hexon E451Q point mutation to evade liver sequestration by the complement Factor X thus enabling intravenous dosing. 3. Deletion of several E3 genes (Δ12.5 K, ΔCR1a0, Δgp19k, ΔRIDa, ΔRIDb, Δ14.7 K) to enhance viral cytotoxicity in tumor cells. 4. Chimeric Ad5 fiber with Ad34 knob for enhanced tropism. 5. YPet-P2A-ADP to allow visualization of viral infection. Ad5 adenovirus serotype 5, Ad34 adenovirus serotype 34, ADP adenovirus death protein, E1A early region 1A, E3 early region 3, E2F early region 2 transcription factor, E4orf6/7 early region 4 open reading frame 6/7, IV intravenous, YPet yellow fluorescent reporter protein. Also refer to Supplementary Table 1. Figure created with BioRender.com.

Modifications to enhance virus replication, lysis, and spreading in tumor cells for ICVB-1042 are illustrated in Fig. 1.

### ICVB-1042 binds and enters cells via human CD46

Infectivity and viral replication of ICVB-1042 can be monitored through the expression of its genetically encoded YPet protein, which is expressed during active viral replication in infected cells. ICVB-1042 incorporates a chimeric Ad5/Ad34 fiber, designed to enable cell entry through huCD46 on target cancer cells^34,35^, while Wt Ad5 relies on CAR for entry (Supplementary Table 1).

**Fig. 2:**
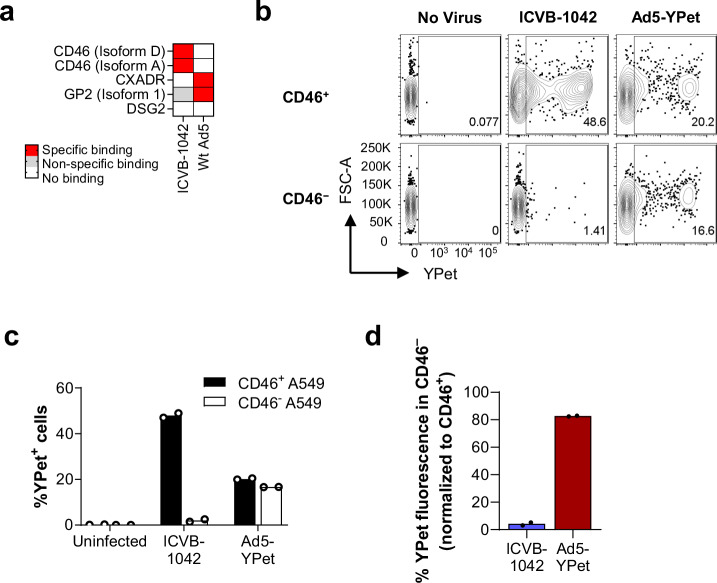
ICVB-1042 binds and enters A549 cells via CD46. **a** Human plasma membrane proteins (6101 full-length proteins and 396 heterodimers) were screened in technical duplicate for binding to ICVB-1042 and Wt Ad5 using the Retrogenix™ Cell Microarray Technology. Subsequent confirmatory screening (n = 34 interactions) in technical duplicate revealed specific binding of ICVB-1042 to CD46 isoform D and CD46 isoform A while Wt Ad5 bound to CXADR (CAR) and glycoprotein 2 (GP2). Neither virus specifically bound to desmoglein 2 (DSG2). **b**–**d** YPet fluorescence on CD46+ A549 and CD46− A549 cells were assessed following incubation with ICVB-1042 or Ad5-YPet (24 h at MOI = 10) by flow cytometry. **b** Representative flow cytometry plots showing YPet fluorescence on CD46+ A549 and CD46− A549 cells. **c** Percentages of YPet+ cells. Bars represent means of technical duplicates. **d** Relative percentage of YPet+ cells within CD46− A549 cells, normalized to percentage of YPet+ cells within CD46+ A549 cells.

To confirm the altered binding pattern of ICVB-1042 compared to Wt Ad5 toward human cell surface proteins, Retrogenix^TM^ Cell Microarray Technology was used to screen a total of 6101 full-length plasma membrane proteins and an additional 396 human heterodimers (Supplementary Data 6). This revealed the specific interactions of Wt Ad5 with CAR and GP2 (Fig. 2a). In contrast, ICVB-1042 showed binding to huCD46 but no other plasma membrane proteins tested, including receptors utilized by other Ads such as desmoglein 2 (DSG2)^8^.

Next, CD46 knockout A549 cells were generated by CRISPR/Cas9 to test the requirement of CD46 for ICVB-1042 entry. The in vitro infectivity of ICVB-1042 was compared to that of Ad5-YPet, a modified version of Wt Ad5 expressing YPet while retaining the Ad5 fiber knob. Analysis of YPet expression revealed a significant reduction in ICVB-1042 infection in the absence of CD46 protein (48% YPet^+^ in CD46^+^ cells versus 2.0% YPet^+^ in CD46^-^ cells), whereas Ad5-YPet infected both cell types with similar efficiencies as expected (Fig. 2b–d). These data confirm that the Ad5/Ad34 chimeric fiber endows ICVB-1042 with the ability to infect cancer cells through CD46.

ICVB-1042 did not infect cells from mice, rats, hamsters, cotton rats, rabbits, dogs, cynomolgus monkeys, or pigs (Supplementary Table 2). We reasoned that this was likely attributed to the restricted expression of CD46 in non-human species and/or limited homology of CD46 protein sequences across species (Supplementary Fig. 1), a feature known to govern the narrow species-specificity of viruses binding to CD46^36–38^. Additionally, non-human cells may be unable to support the replication of ICVB-1042 following cell entry, as has been observed for other human Ad OVs^12^. Experiments examining viral infection of the mouse LL/2 cell line expressing human CD46 support that ICVB-1042 does not replicate upon entering non-human cells (Supplementary Fig. 2) These findings are consistent with previous observations that group B Ad fibers bind CD46^12,34^.

### ICVB-1042 viral activity in human tumor cell lines and dissociated tumor cells

CD46 is expressed on most tumor types^39^ (Supplementary Fig. 3) and may therefore enable ICVB-1042 to infect a wide range of tumor cells. The anti-tumor activity of ICVB-1042 was evaluated in various human tumor cell lines, including 72 solid tumor and 4 hematologic cell lines, representing 14 different tumor types to aid in the selection of clinical indications. ICVB-1042 replication kinetics in each cancer cell line was compared to those in the permissive A549 cell line. The rate of ICVB-1042 replication in each cancer cell line was quantified as the RelT80, representing the time taken to reach 80% maximum YPet fluorescence intensity, normalized to that observed in A549 cells. ICVB-1042 showed infectivity in all 72 solid tumor lines screened, with RelT80 values ranging from 40.5% to 123.9% (Supplementary Table 3). However, ICVB-1042 did not show infectivity in B cell or T cell lymphoma cell lines and exhibited modest activity (RelT80 < 50%) in a myeloma cell line (Supplementary Table 3) suggesting that ICVB-1042 would be more appropriate for the treatment of solid tumors.

**Fig. 3:**
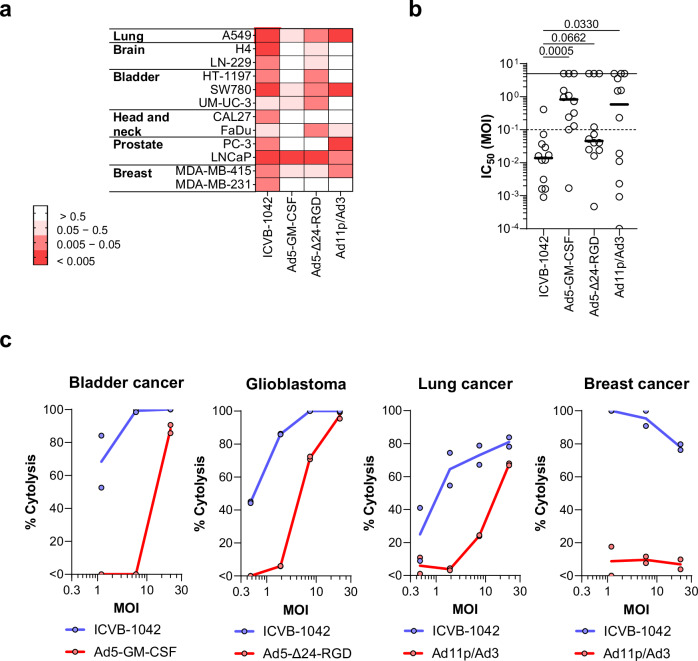
The lytic potency of ICVB-1042 in tumor cell lines and dissociated tumor cells. All viruses had respective dose levels calculated for fair comparison whereby an equal number of infectious virions were used for each virus **a**, **b** ICVB-1042 exhibited broader lytic potency across a wide range of cancer cell lines compared to other Ad OVs. 12 solid human tumor cell lines representing 6 different tumor types were infected with ICVB-1042, Ad5-GM-CSF, Ad5-Δ24-RGD, or A11p/Ad3. IC_50_ MOIs of the viruses were determined for each cell line. A lower IC_50_ MOI indicates higher cytolytic activity. In cases where the IC_50_ MOI could not be derived due to low cytotoxicity, a value of 5 was assigned. **a** Heatmap summary of IC_50_ MOIs. b IC_50_ MOIs of the viruses were graphed in a scatter plot. Each symbol represents a cell line, and the horizontal bars represent the median. Statistical analysis was performed using One-Way ANOVA with Dunnett’s post-hoc test. **c** ICVB-1042 exhibited superior lytic potency in primary dissociated tumor cells compared to the Ad OVs tested in the corresponding cancer type in the clinic. ICVB-1042 was compared to Ad5-Δ24-RGD (a similar Ad OV has been tested in glioblastoma patients), Ad11p/Ad3 (a similar Ad OV has been tested in colorectal and non-small cell lung and colorectal cancer patients), and Ad5-GM-CSF (a similar Ad OV has been tested in bladder cancer patients). Solid lines represent the means of technical duplicates. Data shown are results from one donor per indication, representative of a total of n = 2 donors per indication. Also see Supplementary Fig. 5.

To further assess the anti-tumor activity of ICVB-1042, its cytolytic effects were evaluated in 12 solid human tumor cell lines representing 6 different tumor types, and comparative analysis was performed with other Ad-based OVs (Fig. 3a, Supplementary Table 1). ICVB-1042 was evaluated in various tumor cell lines to assess tumor selectivity, with its activity assessed relative to closely matched primary cell types. The A549 cell line, derived from human adenocarcinoma of alveolar basal epithelial cells, was compared to primary intestinal epithelial cells (Supplementary Fig. 4). For the H4 cell line, originating from neuroglioma in the central nervous system (CNS), astrocytes - also CNS-derived, were used as the appropriate control. CNS-derived primary cells are more suitable for comparing H4 cells than cells from larger organs outside the CNS. Additionally, due to the limitations in culturing primary cells, the best-matched cell types capable of growing in culture were used as controls to evaluate the activity of ICVB-1042. Ad5-GM-CSF is an Ad5 OV that encodes granulocyte-monocyte colony-stimulating factor (GM-CSF). CG0070, an Ad5 OV similarly encoding GM-CSF, has been clinically tested in patients with bladder cancer^40^. Ad5-Δ24-RGD is an Ad5 OV with 24-base pair deletion in the conserved region 2 (CR2) domain of E1A and bearing Arg-Gly-Asp (RGD) sequence in the HI loop of the Ad5 fiber knob. DNX-2401, an Ad5 OV incorporating similar modification, has been clinically tested in glioblastoma patients^41^. Ad11p/Ad3 is a chimeric OV that combines elements of Ad11p and Ad3. A similar virus, enadenotucirev, has been clinically tested in patients with non-small cell lung carcinoma and colorectal cancer patients^42^. ICVB-1042 exhibited cytotoxicity in all tested tumor cell lines (Fig. 3a). The half-maximal inhibitory concentration (IC_50_) MOI for ICVB-1042 was below 0.1 in 11 of 12 tumor cell lines. In contrast, Ad5-GM-CSF, Ad5-Δ24-RGD, and Ad11p/Ad3 exhibited IC_50_ MOI below 0.1 only in 1, 8, and 5 cell lines, respectively, out of the 12 cell lines tested (Fig. 3a, b). On average, ICVB-1042 displayed greater potency than other engineered Ad OVs in tumor lines tested.

To confirm the observed cytolytic potency of ICVB-1042, primary dissociated tumor cells from glioblastoma, lung cancer, bladder cancer, and breast cancer were assessed, comparing ICVB-1042 to our engineered versions of clinical stage OVs being tested for these specific indications (Ad5-GM-CSF, Ad5-Δ24-RGD, and Ad11p/Ad3, respectively). These analyses revealed that ICVB-1042 exhibited substantially higher potency than other engineered Ad OVs in corresponding cancer indications (Fig. 3c, Supplementary Fig. 5). These findings demonstrated a marked improvement in lytic potency of ICVB-1042 across diverse tumor types compared to previous generations of Ad OVs.

### E2F host-dependent activity is required for ICVB-1042 replication

CD46 is widely expressed not only across different solid tumor types but also in healthy tissues (Supplementary Fig. 3). To ensure preferential replication within tumors, ICVB-1042 has been engineered with a small deletion in E1A, preventing its interaction with Rb1 to bypass a cell cycle checkpoint. This renders the virus dependent on host-driven cell cycle progression frequently observed in tumor cells with dysfunctional E2F signaling. In addition, E4orf6/7 has been shown to enhance transactivation of E2F-DNA complexes thereby synergizing with E1A activity. Thus, we hypothesized that the elimination of E4orf6/7 would confer additional tumor-selective properties.

**Fig. 4:**
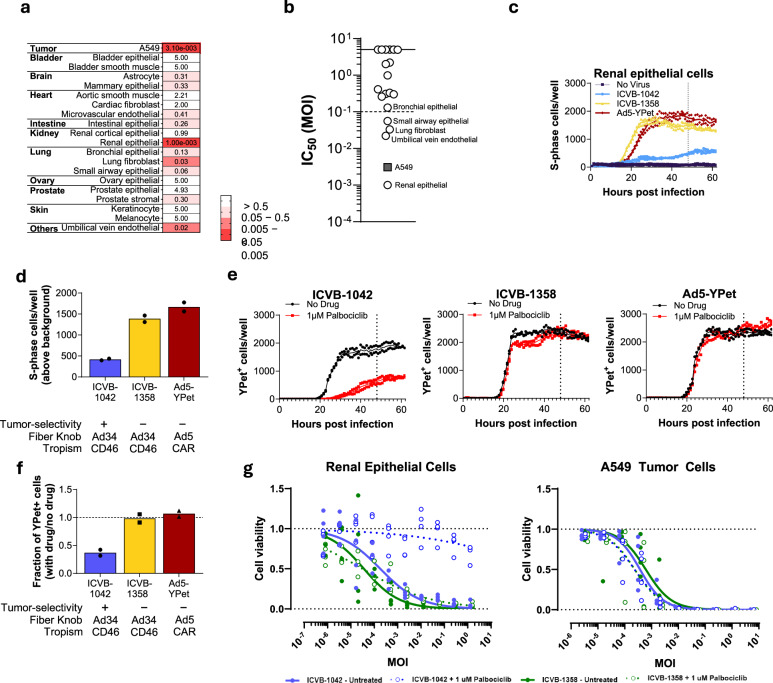
Characterization of ICVB-1042 tumor selectivity. **a**, **b** A panel of 19 human primary cell types from 10 tissues of different origins was examined. Primary cells were infected with ICVB-1042 and IC_50_ MOIs were determined for each cell type. A lower IC_50_ MOI indicates higher cytolytic activity. In cases where the IC_50_ MOI could not be derived due to low cytotoxicity, a value of 5 was assigned. **c**, **d** Primary normal (non-transformed) renal epithelial cells (HREs) expressing mCherry-geminin were infected with ICVB-1042 (blue), ICVB-1358 (yellow; E1AWT and E4orf6/7WT), or Ad5-YPet (red) at MOI of 10, or left uninfected in the presence of 1 µM palbociclib. Cells positive for mCherry-geminin expression were tracked over time to determine cells entering the S-phase. **c** Longitudinal analysis of the number of S-phase cells per well (mean represented by solid line) revealed ICVB-1042 did not robustly induce cell cycle progression of renal epithelial cells in the presence of palbociclib. **d** S-phase cells (solid bars represent the means) at 48 h (n = 2 technical replicates). See Supplementary Fig. 4c–f for data generated with bronchial epithelial cells (BrECs). **e**, **f** Normal HREs were infected with the same viruses as in (**c**) and (**d**) in the presence or absence of 1 µM palbociclib. **e** Longitudinal analysis of YPet+ cells per well (means represented by solid lines) reveals palbociclib reduced ICVB-1042 activities in normal HRE, but no reduction of ICVB-1358 or Ad5-YPet activities, by palbociclib (n = 2 replicates). **f** Fraction of YPet+ cells (means represented by solid bars) at 48 h (n = 2 technical replicates). See Supplementary Fig. 4G, H for data generated with BrECs. **g** WST-1 assays examining cytolytic activity of ICVB-1042 (blue) and ICVB-1358 (green) in normal HREs and A549 cancer cells in the presence (open circles) or absence (filled circles) of 1 µM palbociclib. Lines represent the mean, n = 4.

To evaluate the tumor selectivity of ICVB-1042, we assessed its potential to infect normal human cells relative to the A549 tumor cell line. A panel of 19 human primary cell types from 10 tissues of different origins was examined. ICVB-1042 activity was lower in 18 of 19 human primary normal cells tested compared to the A549 tumor cell line (Fig. 4a, b, Supplementary Fig. 4). However, ICVB-1042 had notable cytolytic activity (IC_50_ MOI < 0.1) on a minority (4 of 19) of primary cells tested, including renal epithelial cells (HRE) (Fig. 4a, b, Supplementary Fig. 4). It is important to consider that these cells were proliferating in vitro, which may not accurately reflect their physiological state in vivo. Accordingly, we observed a positive correlation between the measured cytolytic activity in normal primary cells and their proliferative state at the time of the assay (Supplementary Fig. 6A).

To test ICVB-1042 activity in non-cycling primary normal cells, HRE and bronchial epithelial cells (BrEC) were treated with the CDK4/6 inhibitor palbociclib. Palbociclib is a highly selective ATP-competitive inhibitor that blocks the phosphorylation of Rb, thereby stabilizing Rb-E2F repressive complexes to inhibit E2F activity^13,14^. As expected, palbociclib significantly inhibited the in vitro proliferation of HRE and BrEC (Supplementary Fig. 6B). We compared ICVB-1042 to Ad5-YPet and ICVB-1358, a variant of ICVB-1042 with wild-type E1A and E4orf6/7 (Supplementary Table 1), using HRE or BrEC expressing mCherry-geminin (1-110)^43^ to enable visualization of cell cycle status (i.e., G1 or S-phase). ICVB-1358 and Ad5-YPet infection induced potent S-phase entry in palbociclib-treated cells, consistent with Wt E1A and E4orf6/7 function, whereas ICVB-1042 infection resulted in nearly 3-fold less S-phase entry (Fig. 4c, d, Supplementary Fig. 6C–H). Consequently, palbociclib treatment steeply reduced ICVB-1042 viral replication, but had no effect on ICVB-1358 or Ad5-YPet infections (Fig. 4e, f, Supplementary Fig. 6I, J). Palbociclib treatment proved ineffective in restraining the in vitro proliferation of A549 tumor cells (Supplementary Fig. 6B), known for dysregulated E2F activity^44^. Whereas the cytolytic activity of ICVB-1042 in HRE was notably subdued in the presence of palbociclib, its cytolytic activity in A549 cancer cells remained unaffected by palbociclib (Fig. 4g). Palbociclib had no effect on the cytolytic activity of ICVB-1358 in HRE or A549 tumor cells (Fig. 4g). These findings suggest that the tumor-selectivity mutations in ICVB-1042 do not compromise its lytic activity in hyperproliferative tumor cells.

Ad5-Δ24-RGD-YPet, incorporating E1A^Δ24bp^ tumor-selectivity modification while retaining wild-type E4orf6/7, demonstrated a comparable capacity to induce S-phase entry of HRE and BrCE as Ad5-YPet in the presence of palbociclib (Supplementary Fig. 6C–G). Accordingly, unlike ICVB-1042, Ad5-Δ24-RGD-YPet displayed significant viral activity in HRE and BrEC even in the presence of palbociclib treatment (Supplementary Fig. 6K–N). These data suggest that the combined modifications of E1A and E4orf6/7 in ICVB-1042 act in concert to prevent S-phase induction or viral replication in quiescent non-cancerous cells.

In silico analyses were additionally performed to evaluate the risk of potential off-tumor activity of ICVB-1042. Consistent with previous reports^45^, tissues with high-turnover rates, such as epithelial cells in the gastrointestinal tract and the lungs, exhibited high levels of proliferation markers (Supplementary Fig. 3) and these sites represent tissues with potential concerns for off-tumor activity of ICVB-1042, similar to other anti-proliferative agents used clinically. Notably, immune cells, which often exhibit high proliferation rates in vivo, are not permissive to ICVB-1042 (Supplementary Table 2). These data collectively underscore that the dual modifications of E1A and E4orf6/7 in ICVB-1042 reduce its capacity to induce S-phase cell cycle checkpoint transition in non-cancerous cells, increasing its reliance on dysregulated E2F1 transcriptional activity in tumor cells.

### Capsid modification enables intravenous administration of ICVB-1042

**Fig. 5:**
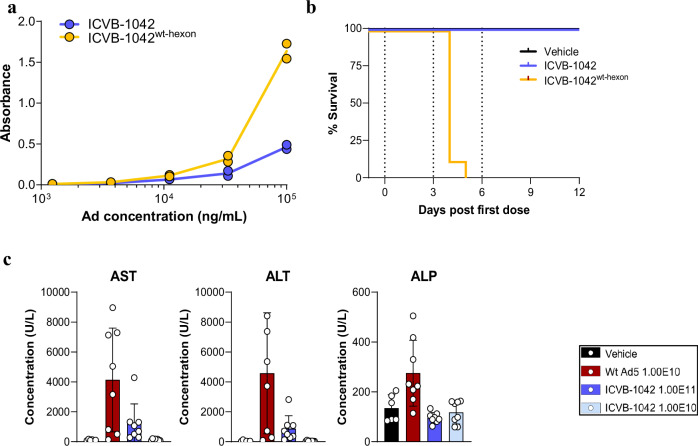
The hexon modification enables systemic administration of ICVB-1042. **a** In vitro binding of ICVB-1042 and ICVB-1042wt-hexon to human FX was assessed by ELISA. Representative of two independent experiments; nude, female mice 6–8 weeks old. **b** Survival of nude, female, 6–8 week old mice bearing SW780 bladder tumor xenograft after IV treatment with ICVB-1042 (blue), ICVB-1042wt-hexon (yellow), or vehicle (black). Vertical dash lines represent dosing days. n = 8 mice per group. **c** Levels of liver enzymes AST, ALT, and ALP in plasma were monitored in C57BL/6 male and female mice, which were 6–7 weeks old at implant, 24 h after IV administration of ICVB-1042 (dark blue, 1E11 VP/dose; light blue, 1E10 VP/dose), Wt Ad5 (red, 1E10 VP/dose), or vehicle (black) in C57BL/6 mice. n = 8 mice per group.

The propensity of Ads to interact with FX, leading to liver targeting, is a major factor contributing to their hepatotoxicity and limiting their systemic administration for oncolytic applications^15^. ICVB-1042 incorporates a hexon modification intended to reduce FX binding. In vitro assessment confirmed reduced FX binding with ICVB-1042 compared to its Wt Ad5 hexon variant (ICVB-1042^wt-hexon^; Fig. 5a, Supplementary Fig. 6A). FX binding is reported to protect Ads from natural IgM^17^. Serum neutralization assays revealed ICVB-1042 is neutralized by IgM or complement in mouse, but in humans IgG is likely the dominant neutralizing factor (Supplementary Fig. 7). To assess if reduced FX binding with ICVB-1042 in vitro translates to improved tolerability following IV administration, Nude mice bearing subcutaneous SW780 human bladder carcinomas were treated IV with high dose levels of ICVB-1042, ICVB-1042^wt-hexon^, or vehicle control on days 0, 3, and 6 (2E10 PFU per dose). All animals (8 of 8) treated with ICVB-1042^wt-hexon^ exhibited severe clinical manifestations and did not survive beyond the third administration. By contrast, none (0 of 8) of the animals treated with ICVB-1042 died (Fig. 5b).

Acute toxicity studies were additionally conducted with C57BL/6 mice to evaluate liver enzyme levels in serum, comparing IV administration of ICVB-1042 (at doses 1.00E11 or 1.00E10 VP) and Wt Ad5 (at a dose of 1.00E10 VP) dosed daily for three consecutive days. While Wt Ad5 induced transient elevations in aspartate aminotransferase (AST), alanine aminotransferase (ALT), and alkaline phosphatase (ALP), ICVB-1042 (1.00E10 VP) had limited effect on these enzymes, resulting in notably no elevation at 24 h post the third dose. At a 10-fold higher dose than Wt Ad5 ICVB-1042 had no effect on ALP and a similar effect on AST and ALT (Fig. 5c, Supplementary Fig. 8B). Systemic pro- and anti-inflammatory cytokine responses driven by IV administration of ICVB-1042 or Wt Ad5 were similar, with both viruses inducing transient elevation of IFNγ, IL-10, IL-6, and TNFα (Supplementary Fig. 9). Collectively, these findings demonstrate that the capsid modification in ICVB-1042 reduces liver toxicity commonly observed with systemic administration of Ad OVs^46^, underscoring the improved tolerability profile of ICVB-1042.

ICVB-1042 displayed expected dose-dependent pharmacokinetic profiles and biodistribution patterns in immune-competent mice following IV administration (Supplementary Figs. 10 and 11). Histopathological examination revealed no notable toxicity from systemic administration of ICVB-1042. Any observed findings were transient and mild and in line with patterns seen in mice systemically exposed to viruses^47–49^. Collectively, these data provide evidence that the hexon modification in ICVB-1042 reduces its binding to FX, thereby decreasing the potential for hepatotoxicity. IV administration of ICVB-1042 was well tolerated. This improvement in tolerability potentially enhances the therapeutic index of ICVB-1042 compared to other clinically tested Ad OVs.

### In vivo anti-tumor activity of ICVB-1042 administered IV or IT

**Fig. 6:**
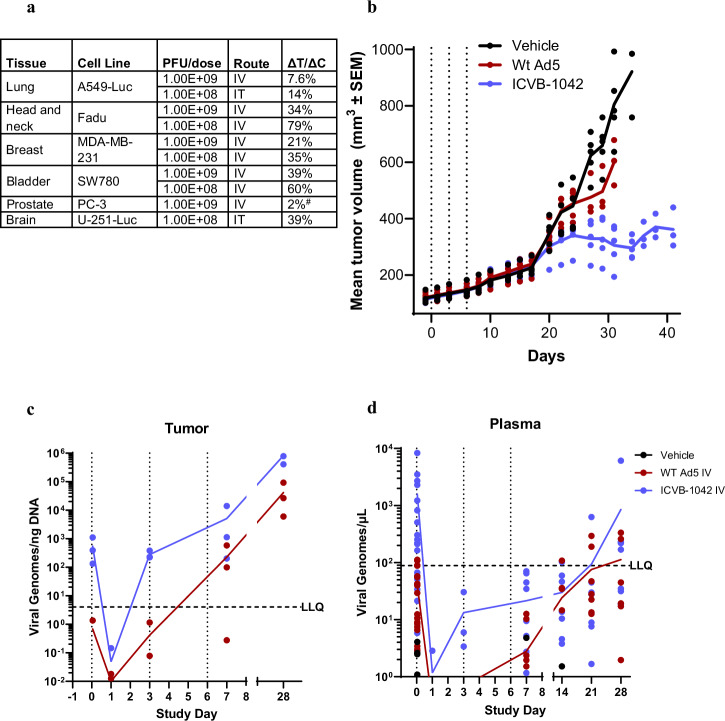
Efficacy of ICVB-1042 in human tumor xenograft mouse models. **a** Efficacy of IV or IT administration of ICVB-1042 was evaluated using various human tumor xenograft models in NSG mice (subcutaneous or orthotopic). Tumor growth inhibition by ICVB-1042 was evaluated by calculating the delta treatment (ΔT)/delta control (ΔC) ratio. Note that PC-3 tumor growth inhibition was determined based on measurements with bioluminescent imaging (BLI) rather than caliper measurements. In all models, mice previously implanted with tumors were treated with three administrations of ICVB-1042 or vehicle IV or IT on days 0, 3, and 6. TGI graphs and overall survival for each model can be found in Supplementary Fig. 11. **b**–**d** NSG, female mice bearing orthotopic human breast carcinoma tumors (6–7 weeks old at implant) were treated IV with ICVB-1042 (red) or Wt Ad5 (blue) (2.00E8 PFU), or vehicle, on day 0, 3, and 6. **b** Mean tumor volume, represented by solid lines (n = 5 animals per group). Data has been excluded when less than 50% of animals were alive. Statistical test with One-way ANOVA followed by a post-hoc pairwise comparison (Holm–Sidak) on Day 28 post the first ICVB-1042 administration indicated a significant (p < 0.05) difference between ICVB-1042 and Wt Ad5. **c** Viral genome copies per ng DNA recovered. **d** Viral genome copy concentrations per µL of plasma.

Anti-tumor activity of ICVB-1042 in vivo was evaluated in immune-deficient NSG mice using various human carcinoma xenograft models. ICVB-1042 displayed broad in vivo efficacy following IV administration (Fig. 6a, Supplementary Fig. 12), consistent with in vitro data showing its potency across different cancer types (Fig. 3a, b). There was dose-dependent anti-tumor activity of ICVB-1042 as expected, and ICVB-1042 administered IT also displayed robust anti-tumor efficacy (Fig. 6a, Supplementary Fig. 12).

The efficacy of ICVB-1042 was compared to that of Wt Ad5 using a triple-negative breast cancer (TNBC) orthotopic xenograft model in which MDA-MB-231 cancer cells were implanted in the mammary fat pad. At 2.00E8 PFU dose level, IV administration of either ICVB-1042 or Wt Ad5 was well tolerated and did not lead to acute weight loss or deaths. Treatment with ICVB-1042 inhibited MDA-MB-231 tumor growth compared to vehicle control (median ΔT/ΔC of 30%), whereas treatment with Wt Ad5 had a more modest impact (median ΔT/ΔC of 76% at Day 28) (Fig. 6b). An ANOVA test followed by a post-hoc pairwise comparison by the method of Holm–Sidak was used to compare tumor volume on day 28. These differences in tumor volumes (primary endpoint) were significant (p < 0.05). ICVB-1042 treatment also resulted in increased overall survival relative to Wt Ad5-treated animals, although overall survival was not a primary goal of the study, (Supplementary Fig. 13). ICVB-1042 was detected in the tumor following IV administration, exhibiting a progressive increase (Fig. 6c), indicating successful viral replication within the tumor. The viral genomes in tumors of ICVB-1042-treated tumors consistently exceeded those of Wt Ad5-treated tumors, likely reflecting more extensive replication of ICVB-1042 compared to Wt Ad5. Viral genome copies detected in the plasma increased over time (Fig. 6d), indicating that the viruses continued to enter circulation after lysing tumors. Notably, there was no evidence of ICVB-1042 replication in mice not bearing tumors following IV administration (Supplementary Fig. 14).

### Induction of immune responses by ICVB-1042 infection of cancer cells

**Fig. 7:**
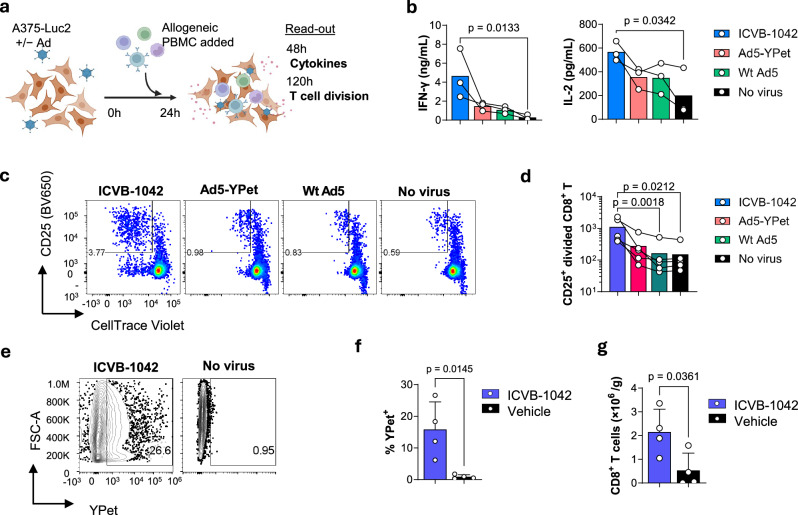
Immune responses elicited by cancer cells infected with ICVB-1042. **a**–**d** Primary PBMCs from healthy donors were stimulated by coculturing with A375-Luc2 human melanoma cells pre-infected with ICVB-1042, Ad5-YPet, Wt Ad5 at an MOI of 15, or no virus. **a** Schematic of the coculture experiment. **b** IFN-γ and IL-2 in the culture supernatant at 48 h were quantified using the Meso Scale Discovery platform. **b** Representative flow cytometry plots showing proliferation of CD8+ T cells at 120 h. Note that divided CD8^+^ T cells are identified as CTV^low^ CD25^+^. The number in the gate indicates the percentage. **c** Quantification of divided CD8+ T cells at 120 h. See Supplementary Fig. 16 for the gating strategy. **b**, **d** Each symbol represents the average (mean) value of an assay run for a PBMC donor in duplicate, n = 3 (**b**) and n = 5 (**d**). Matched data from the same PBMC donors are connected by lines. The bar represents the geometric mean. Statistical analysis was performed using the Friedman test, followed by Dunn’s post-test. Data are representative of 2 independent experiments. **e**–**g** Humanized NCG, female, 25-week-old mice (note that these mice were older since they were humanized which is a 12–14-week process) subcutaneously harboring A549 human lung carcinoma were injected IV with ICVB-1042 (n = 4) or vehicle (n = 4) on day 0, 3, and 6. Mice were sacrificed on day 22 and the tumor was analyzed by flow cytometry. **e** Representative flow cytometry plots showing YPet^+^ A549 lung carcinoma cells (pre-gated as live, HLA-A/B/C^+^ single cells). YPet fluorescence indicates infection with ICVB-1042 in vivo. The number in the gate indicates the percentage. **f** Percentage of YPet^+^ A549 lung carcinoma cells. **g** Tumor-infiltrating CD8^+^ T cells (CD3^+^ CD8^+^ live single cells) per gram of tumor tissue.

Induction of anti-tumor adaptive immune responses is important for long-term control of cancer following oncolytic virotherapy^33^. To assess the impact of ICVB-1042 on the immunogenic potential of cancer cells, allogeneic primary human PBMCs from healthy donors were cocultured with A375-Luc2 human melanoma cells transduced with ICVB-1042, Ad5-YPet, Wt Ad5, or no virus. The induction of early proinflammatory cytokines (IFNγ and IL-2) and the proliferation of allogeneic CD8^+^ T cells were assessed (Fig. 7a).

ICVB-1042 infection of A375-Luc2 cells led to a significant increase in IFNγ and IL-2 production by allogeneic PBMCs (Fig. 7b). In contrast Ad5-YPet or Wt Ad5 infections showed subdued effects, with consistently lower cytokine levels compared to ICVB-1042 infection across all tested PBMC donors. Similar findings were observed with other cancer cell lines (A549-Luc2 and PC-3-Luc2) where ICVB-1042-transduced cancer cells induced more robust cytokine production by allogeneic PBMCs (Supplementary Fig. 15). A375-Luc2 cells transduced with ICVB-1042 induced robust proliferation of CD8+ T cells, as evidenced by dilution of cell division dye (CellTrace Violet; CTV) and CD25 upregulation (Fig. 7c, d, Supplementary Fig. 16 for gating strategy). By contrast, A375-Luc2 cells transduced with control Ad5 viruses or uninfected A375-Luc2 cells induced very little CD8+ T cell proliferation, with approximately a 10-fold reduction compared to ICVB-1042-transduced A375-Luc2 cells (Fig. 7c, d). Thus, infection with ICVB-1042 increased the immunogenic potential of cancer cells to stimulate proinflammatory cytokines and T cell responses.

Interactions of ICVB-1042, cancer cells, and the immune system were modeled in vivo using A549 tumor-bearing humanized NCG (huNCG) mice. After three IV doses of ICVB-1042, a fraction of tumor cells had detectable YPet fluorescence (Fig. 7e, f), indicating successful targeting of tumors and active ICVB-1042 infection in the presence of an intact humanized immune system. Interestingly, there was a modest increase in tumor-infiltrating CD8+ T cells in ICVB-1042-treated mice compared to control, suggesting that lysis of tumor cells by ICVB-1042 following IV administration can enhance recruitment and/or activation of CD8+ T cells in tumor microenvironments.

## Discussion

ICVB-1042 was generated through rational engineering of Ad genomes to enable targeting various cancer types through expanded tropism, increased potency, improved tolerability, and enhanced ability to engage anti-tumor T cell responses. Here, we have shown that ICVB-1042 exhibits broad and potent anti-tumor effects in vitro and in vivo. The selectivity and tolerability of ICVB-1042 have enabled IV administration at high doses, facilitating systematic targeting of a wide spectrum of tumor types. These promising preclinical findings provide strong rationales for the ongoing clinical evaluation of ICVB-1042.

Traditional Ad5-based OVs have faced challenges due to inconsistent expression levels of their primary cell entry receptor, CAR, on cancer cells. A prevalent method to tailor the tropism includes modifications to the fiber knob that controls receptor binding. ICVB-1042 combines in its design the knob from Ad34 with the shaft from Ad5, creating a chimeric fiber. This design enables the virus to enter cells expressing human CD46, which is widely expressed in tumor cells. Our data using cell microarray technology validated that ICVB-1042 binds selectively to CD46, and knockout studies confirmed the requirement for CD46 for ICVB-1042 entry into cells.

Potential off-tumor toxicities of ICVB-1042 have been mitigated through dual tumor-selective modifications of E1A and E4orf6/7. Past strategies to enhance tumor selectivity in Ad OVs include a 24-base pair deletion in the E1A gene (Δ24), which was employed in DNX-2401, ONCOS-102, ORCA-010, and CAdVec. This mutation prevents E1A from binding to the pRb protein to bypass a cell cycle checkpoint. Other strategies involve the use of tumor-associated promoters to drive the expression of E1A, like CG0070 (E2F1 promoter)^50^, OBP-301 (hTERT promoter)^51^, CRAd-S-pk7 (Survivin promoter)^52^, and AdVince (CgA promoter)^53^, that can be combined with d24 mutation to further improve selectivity, like in SynOV1.1^54^, VCN-01^55^, TILT-123^56^, or LOAd-703^57^. However, disruption of transcriptional control of E1A via modification of the wild-type promoter has been shown to impair oncolytic potency when broadly tested across tumor models^58,59^. The E1A^Δ15bp^ employed in ICVB-1042 represents a more conservative deletion of the Rb interaction motif (LxCxE) intended to preserve the E1A function.

Another mutation commonly used is the deletion of the E1B-55K gene, which has been suggested to enable tumor-selective replication in p53-null cells, although the mechanism has been disputed and is not fully understood^60,61^. Only about half of human cancers harbor p53 mutations^62^, severely limiting the indications in which this approach would be effective. This modification is found in ONYX-15 and Xvir-N-31. Variants to improve tumor selectivity have also been developed by using libraries of recombinant virus and guided evolution such as ColoAd1^63,64^ containing multiple mutations in E2B, E3, and E4 region, although the exact mechanism that confers improved tumor selectivity is not fully understood. ICVB-1042 introduces modifications in E1A and E4orf6/7 that, when combined, produced an improved selectivity and dependency on proliferating cells. Though the exact mechanism warrants further exploration, we speculate that E1A and E4orf6/7 may act on different pathways. While E1A mutation prevents binding to pRb; E4orf6/7 has been described to inhibit DNA damage response to enable efficient viral replication^65^. DNA repair pathways are commonly inactivated or suppressed in tumor cells, hence elimination of E4orf6/7 may facilitate replication in cells with low DNA damage sensing response.

In addition to tumor selectivity, hepatotoxicity has been another limiting factor for the IV administration of Ad OVs. This toxicity has been postulated to arise from the interaction between Ad5 hexon proteins and FX. To counteract this, ICVB-1042’s hexon has been modified to minimize binding to FX, aiming to reduce liver sequestration and thus, the associated toxicity. In vivo studies demonstrated significantly enhanced tolerability compared to Wt Ad5 and the version of ICVB-1042 that retained Wt Ad5 hexon. Of note, the elimination of FX binding is expected to sensitize ICVB-1042 to natural IgM binding in mice and a subset of humans^17,66^. Systemic cytokine responses, likely driven by innate recognition of ICVB-1042 viral components, were modest and transient.

YPet has enabled detailed preclinical characterization of ICVB-1042 activity in cancer cells. Pharmacokinetic studies measuring viral genomes in plasma and tumors showed clear evidence of long-term viral replication potential in tumors. YPet levels in blood and tumors will also be assessed in the ongoing clinical study of ICVB-1042 (NCT05904236). YPet may prove to be a valuable biomarker to evaluate ICVB-1042 activities in patients.

One factor that may impact clinical response to ICVB-1042 is pre-existing immunity, which may be driven by prior exposure to endemic Ads (e.g., Ad5). However, previous clinical studies with Ad5-based VCN-01 did not find a correlation between pre-existing neutralizing antibody titers and anti-tumor response^67^. This lack of correlation may be due to the high viral doses utilized for therapeutic applications compared to the titers that most subjects are exposed to during natural infections. Furthermore, it is expected that ICVB-1042 can be administered at sufficiently high doses to overcome pre-existing immunity as ICVB-1042 has been engineered to improve tolerability.

Compared to other viruses, oncolytic adenoviruses have been shown to be able to activate an immune response more effectively^4^. This innate activity can be further magnified by deleting the early region 3 (E3) group of genes, which encode for a group of immunomodulatory proteins that helps the virus evade immune surveillance by interfering with antigen presentation, cytokine secretion, cell apoptosis, and inflammation response. The E3 genes include E3 gp19K, which inhibits the expression of class I MHC molecules^32^, E3 14.7K, which protects the virus from antiviral responses^68^, and E3 RID, which binds to the leukocyte common antigen CD45 and modulates T cell activation^27^. Additionally, proinflammatory molecules can be engineered in the viral genome to further invigorate the immune response. For instance, GM-CSF expression is utilized in the case of the oncolytic adenovirus CG0070^50^, while IL-12 expression is seen in yCD/mutTKSR39rep-hIL-12^69^. The enhanced lytic activity observed in ICVB-1042 showed the increase in immunogenic cell death and immune activation in vitro, and T cell infiltration in vivo, suggesting ICVB-1042 can efficiently synergize and enhance an anti-tumor immune response without additional engineering.

In summary, the preclinical data presented demonstrate that ICVB-1042 is an engineered oncolytic adenovirus that addresses the limitations of current OV therapies. ICVB-1042 exhibited enhanced lytic capacity, expanded tropism, and tumor selectivity. These attributes allowed ICVB-1042 to be safely administered systemically to treat various solid tumor types as shown using multiple mouse models. These promising preclinical data provide a scientific rationale for the ongoing phase 1 clinical study evaluating ICVB-1042 for various solid tumor indications (NCT05904236).

## Materials and methods

### Adenoviruses

ICVB-1042 and other engineered Ads used in this study were originally generated using a proprietary modular adenovirus assembly platform based on the Gibson assembly method. ICVB-1042 is an Ad5-based engineered virus with key modifications from Wt Ad5 including (1) 15 base pair deletion in E1A and a deletion of E4orf6/7 for enhanced tumor selectivity, (2) chimeric Ad5/Ad34 fiber to target CD46, (3) YPet fluorescent reporter encoded as a YPet-P2A-ADP fusion, (4) modified capsid/hexon to reduce liver sequestration, (5) deletion of 12.5K, 6.7K, 19K, RID-alpha, and 14.7K genes in the E3 region.

Ads used in this study were manufactured in-house or at Viraquest (North Liberty, Iowa, USA). Briefly, adherent A549 cells (ATCC; CCL-185) were used to expand stock viral lysates, and virus purification was performed using cesium chloride gradient ultracentrifugation. The purified viruses were formulated in A195 buffer (10 mM Tris, 10 mM histidine, 75 mM NaCl, 5% sucrose (w/v), 1 mM MgCl2, 0.02% (w/v), PS-80, 0.1 mM EDTA, 0.5% ethanol (v/v), pH 7.4) and stored frozen until use. For each virus lot, viral particle titer and infection titer were determined by OD_260_ and TCID_50_ ELISA, respectively.

### Human cell lines and primary cells

Human cell lines and primary normal cells used in this study were sourced from American Type Culture Collection (ATCC), MilliporeSigma, Ximbio, Japanese Collection of Research Bioresources (JCRB), Lonza Biosciences (Lonza), or Cell Biologics. Refer to Supplementary Table 4 and Supplementary Table 5 for the respective catalogs of all human primary normal cells.

### Screening ICVB-1042-binding proteins

The Retrogenix™ Cell Microarray Technology was used to screen for human proteins bound by ICVB-1042 or Wt Ad5. See Supplementary Data 6 for the list of proteins and heterodimers screened. Briefly, expression vectors encoding the proteins were reverse-transfected into HEK293 cells in duplicate cell microarray spots on glass slides and fixed. A control expression vector (pIRES-hEGFR-IRES-ZsGreen1) was also used in four cell microarray spots on every slide to ensure acceptable transfection efficiency. Transfected cells were fixed and ICVB-1042 or Wt Ad5 was added to each slide at a final concentration of 5 × 10^3^ particles/cell. Cells incubated with the virus were fixed again and detection of binding was performed using rabbit polyclonal anti-adenovirus type 5 antibody (Abcam, Cat# ab6982) and secondary polyclonal anti-rabbit IgG H+L conjugated to Alexa Fluor 647. Fluorescent images were analyzed using ImageQuant software (GE Healthcare, Version 8.2). A binding interaction was identified as a duplicate spot showing a raised signal compared to background levels. Vectors encoding all interactions identified in the library screen were arrayed and expressed in HEK293 cells on new slides for confirmation analyses. Confirmatory analyses were carried out for the library screen after cell fixation in duplicate.

### Tropism of ICVB-1042 for CD46

A549 cells engineered to express a codon-optimized CRISPR-associated protein 9 (Cas9) RNA-guided endonuclease (A549-Cas9) were used to generate A549-Cas9-single guide ribonucleic acid (sgRNA)-CD46 cells in-house. Approximately 50% of the A549-Cas9-sgRNA-CD46 cells lacked the surface expression of CD46. The cells were seeded in 96-well tissue culture plates at a density of 10,000 cells/well and incubated overnight and then infected with ICVB-1042 or Ad5-YPet at an MOI of 10. At 24 h post-infection, the cells were stained using LIVE/DEAD fixable near-IR dead cell stain kit (Invitrogen, Cat# L34976) and anti-human CD46 conjugated to Alexa Fluor 700 (clone MEM-258, Invitrogen, Cat# MA5-28579), followed by fixation with 0.5% formaldehyde (Thermo Fisher Scientific, Cat# 28906) in PBS for 30 min at room temperature. Since the A549-Cas9-sgRNA-CD46 cell line contains both CD46+ and CD46- cells, this allowed for the direct comparison of viral entry/replication efficiencies in these cells within the same experimental well. Data were acquired using BD FACSymphony A3 and analyzed using FlowJo 9 (BD). Single cells were gated on FSC-A versus FSC-H and viable cells were identified as negative for the LIVE/DEAD stain. CD46+ and CD46- negative populations were separated according to the signal of the anti-CD46 stain, using a uniform CD46+ population as a positive control. Viable cells expressing YPet were enumerated for both CD46+ and CD46- cell populations.

LL/2 C57BL/6 Lewis lung carcinoma cells were engineered in-house to express a human CD46 transgene. The resulting LL/2-huCD46 cells, along with the parental LL/2 cell line, were employed to investigate whether the presence of human CD46 protein on murine cells facilitates viral entry and replication. Each cell line was seeded at a density of 15,000 cells per well in 96-well plates coated with rat tail collagen (Sciencell Research Labs, Cat#8188a) and incubated overnight. Subsequently, the cells were infected with ICVB-1042 or Ad5/Ad34-GFP at a MOI of 100 for 24 h. Imaging was conducted using a BioTek Cytation 5 (Agilent) and analyzed with BioTek Gen5 (Agilent).

### Characterization of ICVB-1042 activity in human tumor cell lines

ICVB-1042 activities in human tumor cell lines were first assessed by a YPet fluorescence-based readout. Briefly, cells were plated in 96-well plates at 10,000 cells per well of cell-specific media and incubated overnight. They were then infected with ICVB-1042 over a range of MOI and were incubated in BioTek BioSpa (Agilent) connected with Cytation 5 plate reader (Agilent) for fluorescence intensity measurements every 2 h over a period of 6–10 days. The T80 value is the length of time elapsed post-infection when 80% of the maximum observed YPet intensity is achieved at the interpolated MOI = 0.05. For interpolation, linear regression was conducted on a semi-log graph of log-transformed MOI and 80%-YPet timing across examined MOIs. A relative timing of 80%-maximum YPet intensity (RelT80) value is calculated by dividing the T80 of A549 cells infected with ICVB-1042 by the T80 of a cell line of interest in the same experiment. A higher RelT80 indicates faster virus-induced YPet fluorescence kinetics for the cell line of interest.

As a confirmatory experiment, cytotoxicity of ICVB-1042 and comparator viruses (Ad5-GM-CSF, Ad5-d24-RGD, and Ad11p/Ad3; See Supplementary Table 1) in a panel of cancer cell lines was measured with a colorimetric/metabolic cell viability assay using water-soluble tetrazolium salt (WST-1) 6 days post-infection.

### Characterization of ICVB-1042 activity in primary normal cells

Cells were thawed according to the vendor’s protocol and allowed to recover and expand in tissue culture flasks. A proper cell type-specific media was used for each cell type as indicated by the vendor. Upon reaching confluence, normal human primary cells were resuspended to a concentration of 3.0E5 cells/mL and plated in 96-well plates at 3.0E4 cells per well. Two days post plating, complete growth media was replaced with basal media containing 0.5% fetal bovine serum (FBS) and cells were incubated at 37°C in 5% CO_2_ and 5% O_2_ for a further 2 days. For experiments conducted in the presence of palbociclib, 1 µM palbociclib was added to the cells at this time. A549 control cells were thawed 2 days prior to infection in Dulbecco’s Modified Eagle Medium (DMEM) + 0.5% FBS at 37°C in 5% CO_2_ and 20% O_2_ and allowed to grow for at least 24 h prior to plating. For experiments conducted in the presence of palbociclib, A549 cells were thawed directly into DMEM + 0.5% FBS and 1 µM palbociclib. Three days after thawing, A549 cells were resuspended to a concentration of 1.0E5 cells/mL and plated at 1.0E4 cells/well.

Normal human primary cells and A549 tumor cells were infected from a common plate of serially diluted ICVB-1042. The highest dose of ICVB-1042 was approximately 3.0E5 PFU which was serially diluted through 10 sequential 5-fold dilutions. Cell counts in representative wells were determined on the day of infection through Hoechst 33342 and propidium iodide staining to calculate the MOIs tested. Daily 50% media changes were performed for 6 days following infection. For experiments conducted with palbociclib, fresh 1 µM palbociclib was added during media changes to ensure continued bioactivity. On Day 6 post-infection, a WST-1 assay was performed to determine cell viability of all conditions. Plates were read on a Biotek Cytation 5 instrument. All conditions were performed in triplicate.

IC_50_ MOI values (an MOI level corresponding to 50% cell viability) were derived from non-linear regression (dose-response inhibition model with 4 parameters) using GraphPad Prism. The bottom level of the regression was constrained to 0, whereas the top level was constrained to 1 and the IC_50_ value was constrained to be greater than zero. All other parameters were not constrained. In the event regression could not be performed due to insufficient levels of cytotoxicity, the IC_50_ value could not be determined and instead, an approximation of the IC_50_ is reported as greater than the maximum MOI assayed for that cell type.

### Mechanistic studies of E2F host-dependent activity and ICVB-1042 replication in human primary normal cells

Human primary normal human cells were transduced to express mCherry-Geminin, a biosensor that enables the determination of cell cycle status in single cells. Primary normal cells expressing the biosensor were seeded to optical plates and incubated with or without palbociclib for 24 h to block cell cycle progression. Cells were infected with a virus, and images were collected in brightfield, red, and green channels to quantify S-phase entry (mCherry-Geminin biosensor) and viral infection (YPet) in real-time for 60 h on an xCELLigence RTCA eSight instrument. S-phase entry and viral infection were compared between ICVB-1042 and viruses that have Wt Ad5 selectivity including Ad5-YPet and ICVB-1358 (see Supplementary Table 1). In confirmatory studies, cell culture and viral infection were performed in the presence of the cyclin-dependent kinase 4/6 inhibitor palbociclib at a concentration of 1 µM to restrict the proliferation of primary cells.

### Cytotoxicity of ICVB-1042 and other Ads in primary dissociated tumor cells from patients

All primary dissociated tumor cells from patients, including glioblastoma, lung adenocarcinoma, breast cancer (invasive ductal carcinoma), and bladder cancer (squamous cell carcinoma or transitional cell carcinoma), were procured from Discovery Life Sciences (Supplementary Table 6). A statement of ethics approval for the use of these cells was provided by Discovery Life Sciences. Comparative studies were conducted using ICVB-1042 and our engineered versions of clinically relevant OVs (Ad5-GM-CSF, Ad5-Δ24-RGD, and Ad11p/Ad3) tailored for these specific indications. Frozen glioblastoma primary dissociated cells were thawed and cultured for 7 days in laminin-coated tissue culture flasks (MilliporeSigma, Cat# L2020-1MG), while frozen lung adenocarcinoma, invasive ductal carcinoma, and bladder cancer primary dissociated cells were thawed and cultured for 5 to 14 days in collagen I-coated tissue culture flasks (ScienCell, Cat # 8188). Following the expansion, 10,000 dissociated tumor cells were seeded onto 96-well E-Plates (Agilent, Cat# 300600910). After an overnight incubation, the virus was added, and changes in electrical impedance (cell index) were monitored. Concurrently, cell images (brightfield and YPet fluorescence) were captured over time using xCELLigence RTCA eSight.

### ELISA to evaluate the binding capability of ICVB-1042 to factor X

ELISA plates (Thermo Fisher Scientific, Cat# 3455) were coated with recombinant human factor Xa (R&D Systems, Cat# 1063-SE-010) or recombinant mouse factor Xa (Novus Biologicals, Cat# 6724-se) at a concentration of 2 μg/mL in ELISA Carbonate Coating Buffer (Thermo Fisher Scientific, Cat# CB01100) at 4 °C over-night. Control wells were left uncoated. The next day, wells were blocked using SuperBlock Blocking Buffer (Thermo Fisher Scientific Cat # 37535) for 1 h. Serially diluted ICVB-1042 or ICVB-1042^wt-hexon^, prepared in TBS plus normal goat serum (Thermo Fisher Scientific, Cat# 31873), was added to wells, and incubated for 2 h on a plate rocker. Bound virus was detected using rabbit polyclonal anti-adenovirus type 5 antibody (Abcam, ab6982) and goat anti-rabbit IgG (H+L) secondary antibody conjugated to alkaline phosphatase (Thermo Fisher Scientific, Cat# 31342). Alkaline phosphatase was detected by incubating with PNPP substrate (Thermo Fisher Scientific, Cat#34047) for 10 min in the dark, and color development was performed by incubating with 1-Step TMB ELISA Substrate Solution (Thermo Fisher Scientific, Cat# 34029).

### Flow cytometry

To evaluate the impact of ICVB-1042-infection on the immunogenicity of cancer cells, the proliferative response of human CD8+ T cells was studied in the allogeneic coculture of CTV (Thermo Fisher Scientific, Cat# C34557A)-labeled PBMCs and virus-infected A375-Luc2 cancer cell line. Subsequently, cells were stained with Live/Dead Fixable Near-IR (780) Viability Kit (Thermo Fisher Scientific, Cat# L34994, 1:1000) in PBS, followed by staining with fluorescent antibodies against CD3 (Alexa Fluor 700; Thermo Fisher Scientific, 56-0038-42; 1:25), CD4 (Brilliant Ultraviolet 395; BD, Cat# 563550, 1:50), CD8 (Brilliant Ultraviolet 805; BD, 612889, 1:50), CD19 (Phycoerythrin; BioLegend, Cat# 302208, 1:200), CD25 (Brilliant Violet 650; BD, 740634, 1:200), PD-1 (Brilliant Violet 711; BioLegend, Cat# 329928, 1:200), CD69 (Brilliant Violet 785; BioLegend, Cat# 310932, 1:50), and TCRα/β (PerCP/Cyanine 5.5; BioLegend, Cat# 306724, 1:50) in the presence of HumanTruStain FcX (BioLegend, Cat# 422302), diluted in Brilliant Stain Buffer (BD, Cat# 563794). Cells were then washed, fixed using Cytofix™ Fixation Buffer (BD, Cat # 554655), and prepared in Stain Buffer (FBS) (BD, Cat# 554656). Sphero Blank Calibration Particles 6.0–6.4 μm were introduced to samples for absolute quantification of cell numbers via flow cytometry. Samples were acquired using a BD FACSymphony A5 flow cytometer equipped with a High Throughput Sampler, and data were analyzed using BD FlowJo 10.

CD25+ CTVlow CD8+ T cells (pre-gated on live, lymphocytes, singlets, TCRα/β+, CD8+, CD25+, and CTVlow T cells) that underwent proliferation following stimulation were identified and quantified.

### Quantification of cytokines

For assessment of the production of cytokines IFNγ and IL-2 by human PBMCs following a 24-h stimulation with virus-infected A375-Luc2, A549-Luc2, or PC-3-Luc2 cell lines, cell culture supernatant was collected and subjected to MSD assay. The V-PLEX Human Proinflammatory Panel I (4-Plex) kit (MSD, Cat# K15052D) was customized by adding additional SULFO-TAG-labeled detection antibodies specific to IL-2 (MSD, Cat# B21TV), IL-4 (MSD, Cat# B21TW), and IL-12p70 (MSD, Cat# B21UA-3). The assay was conducted using the manufacturer’s standard method on 1:10 diluted supernatant (5 μL diluted to 50 μL total volume using the assay diluent) with the exception of the use of additional detection SULFO-TAG-labeled antibodies as previously described.

For assessment of systemic cytokine responses induced in mice following IV administration of viruses, the V-PLEX Proinflammatory Panel 1 Mouse Kit (MSD, Cat# K15048D) was used to quantify IFNγ, IL-1β, IL-2, IL-4, IL-5, IL-6, chemokine ligand 1 (CXCL-1), IL-10, IL-12p70, and tumor necrosis factor-alpha (TNFα) present in plasma samples. The assay was performed on thawed plasma samples using the manufacturer’s standard method.

MSD plates were read with an MSD SECTOR Imager 2400, and data analyses were performed using the MSD Discovery Workbench v.4.0.13.

### Quantification of ICVB-1042 and Wt Ad5 genome copy numbers in plasma and tissues

Quantification of ICVB-1042 and Wt Ad5 genome copy numbers was performed using a qualified analytical method to ensure adequate assay performance parameters including assay precision (repeatability and reproducibility), accuracy, specificity, and sensitivity are met in each experiment. DNA was isolated from plasma and tissue homogenates using MagMAX™ DNA Multi-Sample Ultra 2.0 Kit (Thermo Fiser Scientific, Cat# A36570) with the KingFisher^™^ Flex System (Thermo Fisher Scientific, Cat# 5400630). The Bio-Rad droplet digital polymerase chain reaction (ddPCR) platform was used to determine copy numbers of ICVB-1042 or Wt Ad5 viral genomes in each tissue. Briefly, DNA samples were partitioned into droplets using the QX200 Automated Droplet Generator. PCR amplification was performed using PCR primers and a fluorescein-based probe specific for fiber or penton regions specific to each of the viruses (Custom Order, Integrated DNA Technologies). The ICVB-1042 genome was detected using primers and a probe targeting the Ad5/Ad34 chimeric fiber region: forward primer (CCTAAACTAGGAACTGGCCTTAG), reverse primer (GACAGTTAGGTGGAGGGTTTATT), and the FAM-probe (/56-FAM/TGACAGCAC/ZEN/AGGTGCCATTACAGT/3IABkFQ/). The Wt Ad5 genome was detected using primers and a probe targeting the Wt Ad5 penton: forward primer (TCGACACCACCCGTGTGTAC), reverse primer (TGCTGTGGTCGTTCTGGTAGTT), and the FAM-probe (/56-FAM/TGGACAACA/ZEN/AGTCAACGGATGTGGCA/3IABkFQ/). Multiple dilutions of gBlocks^™^ gene fragments (Custom Order, Integrated DNA Technologies) for the ICVB-1042 fiber and Wt Ad5 penton, spanning the assay dynamic range, were evaluated in each experiment as controls. After the PCR reaction, droplets were analyzed using the QX200 Droplet Reader and QX Manager 1.2 Standard Edition software.

### In vivo studies

**Mice.** NOD.Cg-*Prkdc*^scid^*Il2rg*^tm1Wjl^/SzJ (NSG) (Strain #055570), C57BL/6J (Strain #000664), and CD46tg mice (B6.FVB-Tg(CD46)2Gsv/J) (Strain #004971) were obtained from The Jackson Laboratory. Hsd:Athymic Nude-*Foxn1*^*nu*^ mice (Strain #069) were purchased from Envigo (8520 Allison Pointe Blvd, Suite 400, Indianapolis, IN 46250). CD1 mice (ICR) (Strain #022) were purchased from Charles River Laboratories (251 Ballardvale St, Wilmington, MA 01887–1000). NOD-*Prkdc*^*em26Cd52*^*Il2rg*^*em26Cd22*^/NjuCrl (NCG) (Strain #572) were purchased from Charles River Laboratories and humanized at TransCure bioServices (260 Av. Marie Curie, 74160 Archamps, France).

All xenograft studies were conducted at LabCorp (655 Fairfield Court, Ann Arbor, MI, 48108 or 671 S. Meridian Rd. Greenfield, IN 46140 USA) (Fig. 6 and Supplementary Figs. 12–14). Cell lines for xenograft studies were provided by LabCorp. The biodistribution and PK study in CD46tg mice (Supplementary Fig. 11) was performed at LabCorp at the Greenfield site. The studies in Nude mice, C57BL/6 mice (Fig. 5), and CD46tg mice (Supplementary Figs. 8 and 9) were conducted at the IconOVir Bio vivarium (CRL, Spectrum III, 3115 Merryfield Row, San Diego, CA 92121). The distribution and PK study in CD1 mice was conducted at Southern Research (2000 9th Avenue S. Birmingham, AL 35205). Studies in huNCG mice (Fig. 7f, g) were performed at TransCure bioServices (260 Av. Marie Curie, 74160 Archamps, France). See Supplementary Methods for a detailed description of in vivo studies. The age and sex of mice used in studies are described in figure legends.

**IACUCs.** All animal studies were performed in strict accordance with the guidelines and regulations of each institution’s Institutional Animal Care and Use Committee (IACUC) and adhered to the ethical standards governing animal research and each institution’s Standard Operating Procedures. The respective IACUC for each institution approved the animal experiments. The names for each IACUC and protocol numbers and titles are:

*IconOVir*: IACUC Protocol Number #: EB17-010-084

Project Title: Understanding the efficacy and biodistribution of different viruses in xenograft models

*LabCorp:* IACUC Protocol # and Title: AA05 Subcutaneous tumor models for evaluation of test agents

IACUC Protocol # and Title: AA73 Abdominal Orthotopic Tumor Models for Evaluation of Cancer Therapies

IACUC Protocol # and Title: ACUA 22-009 8490442 Efficacy Study

IACUC Protocol # and Title: AA25 Metastatic Tumor Models for Evaluation of Test Agents

IACUC Protocol # and Title: ACUA 21-124 8474318 Biod IV Intratumoral Mouse

IACUC Protocol # and Title: ACUA 21-189 8478553 Tissue Distribution Mouse

*Transcure*: IACUC Protocol Number #: CEEA - 101

Title: Comité d’Éthique Local pour l’Expérimentation Animale – Genevois (CELEAG)

*Southern research:* ACUP No.: 21-04-011B

We have complied with all relevant ethical regulations for animal use. Mice at or nearing euthanasia criteria were euthanized. There are a few animals in vehicle groups (Fig. 5, Supplementary Fig. 12A–C) that went slightly over the limit because the prior day they were not near the limit. Euthanasia criteria for LabCorp IACUC: ACUA 21-124 8474318 Biod IV Intratumoral Mouse was >1500 mm^3^ (Fig. 5). Euthanasia criteria for LabCorp IACUC: AA05 Subcutaneous tumor models for evaluation of test agents was >1500 mm^3^ (Supplementary Fig. 12A). Euthanasia criteria for LabCorp IACUC: AA25 Metastatic Tumor Models for Evaluation of Test Agents was >1000 mm^3^ (Supplementary Fig. 12C). Euthanasia criteria for LabCorp IACUC: AA05 Subcutaneous tumor models for evaluation of test agents, LabCorp IACUC: AA25 Metastatic Tumor Models for Evaluation of Test Agents, LabCorp IACUC: ACUA 22-009 8490442 Efficacy Study, and LabCorp IACUC: AA73 Abdominal Orthotopic Tumor Models for Evaluation of Cancer Therapies were >2000 mm^3^ (Supplementary Fig. 12B, D–F).

### Statistics and reproducibility

All statistical analyses were performed using GraphPad Prism. All data presented are the results of analyses of technical and/or biological replicates as specified in figure legends. Specific statistical tests performed on datasets are indicated in figure legends and/or within the relevant “Methods” and “Supplementary Methods” sections. Data presented are either from one experiment that is representative of multiple independent experiments or pooled data from multiple experiments.

Figure 2a presents a binary data analysis that does not employ statistical tests; however, reproducibility is ensured through confirmatory testing which was conducted upon initial screening.

Figure 2b–d present representative data from at least two experiments. No statistical tests were performed but there were clear (>10-fold) differences in the capability ICVB-1042 to infected CD46-expressing or deficient A549 cells. This was also biologically expected based on the known tropism of group B adenovirus fiber knob and the virus binding analysis shown in Fig. 2a.

In the experiments presented in Fig. 3a, b, cancer cell lines were exposed to various viruses in technical triplicates across two batches. Each cell line was included in one batch, except for A549 cells, which were included in both. The log-transformed data in Fig. 3b were analyzed using one-way ANOVA with Dunnett’s post-hoc test after confirming log-normality via the Shapiro-Wilk test (p < 0.05). Reproducibility was ensured by assessing the relative infectivity of viruses across different cancer cell lines in separate experiments, tracking YPet fluorescence over time. Both approaches consistently showed broad infection of various solid cancer cell lines by ICVB-1042. The data presented in Fig. 3c are from one dissociated tumor cell donor per indication, representative of a total of n = 2 donors per indication. Results from the second donor are shown in Supplementary Fig. 3. ICVB-1042 displayed consistent effects across all tumor types. The data presented in Fig. 4a, b were derived from the average values from two independent experiments per primary cell type. In each experiment, cells were cultured in technical quadruplicate and the average values were reported. All animal experiments were conducted once, typically using 6–8 animals per group. The efficacy of ICVB-1042 was evaluated in various human tumor xenograft models such as SW780, MDA-MB-231, A549, and PC-3 in at least two independent experiments. The FaDu and U-251-Luc models were assessed once but with multiple animals. All ddPCR data presented were generated using qualified assays to ensure assay precision, reproducibility, and accuracy. Statistical analyses for Fig. 7 are detailed within the figure legend.

### Reporting summary

Further information on research design is available in the Nature Portfolio Reporting Summary linked to this article.

## Supplementary information

Supplementary Information

Description of Additional Supplemental Material

Supplementary Data 1

Supplementary Data 2

Supplementary Data 3

Supplementary Data 4

Supplementary Data 5

Supplementary Data 6

Supplementary Data 7

Reporting Summary

## Data Availability

The source data supporting this study’s findings are available in the Supplementary Data files. Source data for Fig. 2 are in Supplementary Data 1. Source data for Fig. 3 are in Supplementary Data 2. Source data for Fig. 4 are in Supplementary Data 3. Source data for Figs. 5 and 6 are in Supplementary Data 4. Source data for Fig. 7 are in Supplementary Data 5. Supplementary Data 6 has the table of the human plasma membrane and secreted proteins screened by Retrgoenix^™^ Cell Microarray Technology. Supplementary Data 7 has source data for Supplementary Figs. 2B, 5, 6E, H, J, L, N, 7, 8A, 9–12, and 14.
