# Supplementary material for: Nonclinical characterization of ICVB-1042 as a selective oncolytic adenovirus for solid tumor treatment
Source: Commun Biol. 2024 Sep 13;7:1132. doi: 10.1038/s42003-024-06839-6 (PMC11399272; doi:10.1038/s42003-024-06839-6)
Supplement: Supplementary file 1 — Supplementary Information [file 42003_2024_6839_MOESM1_ESM.pdf]

Supplemental Methods

Evaluation of ICVB-1042 anti-tumor activity in a subcutaneous model of human bladder carcinoma

The anti-tumor activity of ICVB-1042 was evaluated in NSG mice implanted subcutaneously (SC) with SW780 human bladder carcinoma cells in the high axilla. ICVB-1042 was administered on Days 0, 3, and 6 both intravenously (IV) and intratumorally (IT) at 2 dose levels (2.00E+08 PFU or 1.00E+09 PFU). Tumor growth was evaluated by tumor volume measurements. To assess biodistribution and plasma concentrations of ICVB-1042, ddPCR was performed on tissue samples. Plasma and tumor samples were collected 72 hours post dosing (3 animals per group). Plasma, tumor, liver, spleen, and lung samples were collected at end of life (6 animals per group).

Evaluation of ICVB-1042 anti-tumor activity in an orthotopic model of human breast carcinoma in NSG mice

Human MDA-MB-231 breast carcinoma cells (1.00E+06 cells) were injected into the fourth mammary fat pad of female NSG mice. When the tumors were approximately 123 mm^3 in size, the mice were randomized into 14 groups based on tumor burden. Seven groups contained 5 animals per group and were used for efficacy evaluation. Vehicle, Wt Ad5, or ICVB-1042 were administered every third day for 3 total administrations. End of life for these animals was either death due to the disease or removal from the study when the tumor burden reached >1200 mm^3, body weight loss was >20% and/or severe clinical signs of diseases were observed, or end of the study was reached on 41 days post first administration of ICVB-1042. All animals were observed for clinical signs at least once daily. Individual body weights were recorded 3 times weekly. A gross pathological exam was performed at the time of death for all animals. For the efficacy

assessment, tumor volume was measured by caliper 3 times per week, and animal survival was monitored daily throughout the study. PK and biodistribution of ICVB-1042 and Wt Ad5 viral genomes in plasma and tissues were detected by ddPCR and normalized to plasma volume.

Evaluation of ICVB-1042 versus Wt-Capsid virus in a subcutaneous model of human bladder carcinoma

Nude mice implanted SC with SW780 human bladder carcinoma cells in the high axilla. ICVB-1042 or Wt-Capsid virus was administered intravenously at 2.00E10 PFU per dose when tumors reached a mean value of approximately 125 mm^3. Clinical observations were taken after each dose and overall survival was used as the end point.

Viral Kinetics and Biodistribution of ICVB-1042 Following Repeat Intravenous Administration to Female CD-1 mice

Viral kinetics and biodistribution of ICVB-1042 viral DNA were assessed in plasma and tissues from female CD-1 mice at multiple timepoints after repeated IV administration of ICVB-1042. ICVB-1042 at 2.00E11, 1.00E11 or 2.00E10 viral particles per day or vehicle (phosphate-buffered saline) (N=24/group) once every 3 days (Days 0, 3, and 6). The viral kinetics and biodistribution of ICVB-1042 in plasma and tissues was assessed by quantifying viral genomes in plasma, liver, spleen, brain, heart, lungs, bone marrow of femur, kidneys, and ovaries from samples that were collected at 1, and 24 hours after the first administration and on Days 15, 30, 60, 90 (day of sacrifice). ICVB-1042 viral genomes in plasma and tissues were measured by ddPCR.

Viral Kinetics and Biodistribution of ICVB-1042 Following Repeat Intravenous Administration to Female huCD46Tg C57BL/6 Transgenic Mice and C57BL/6 Littermates

Viral kinetics and biodistribution of ICVB-1042 viral DNA were assessed in plasma and tissues from female huCD46Tg C57BL/6 or C57BL/6 littermate mice at multiple timepoints after repeated IV administration of ICVB-1042. ICVB-1042 at 1.00E11 viral particles per day or vehicle (phosphate-buffered saline) were administered IV to female huCD46Tg C57BL/6 mice or their C57BL/6 littermates (N=36/group) once every 3 days (Days 0, 3, and 6). The viral kinetics and biodistribution of ICVB-1042 in plasma and tissues was assessed by quantifying viral genomes in plasma, liver, spleen, brain, heart, lungs, bone marrow of femur, kidneys, and ovaries from samples that were collected at 0.25, 1, and 24 hours and at 7, 31, and 60 days (day of sacrifice) post first dose. ICVB-1042 viral genomes in plasma and tissues were measured by ddPCR.

Immunotoxicity of ICVB-1042 delivered IV in mice expressing the human CD46 receptor

huCD46Tg and Wt mice received IV injections of ICVB-1042 or Wt Ad5 (dose ranging from 5.00E9 to 2.00E10 VP/day) Q3D×3 on Day 0, Day 3, and Day 6. Blood (plasma) was collected before the first dose (pre-dose) and on Day 7, Day 15, and Day 30. Changes in plasma concentration of 10 cytokines, known to be associated with inflammatory responses, were assessed by MSD.

Immune response to IV administered ICVB-1042 in humanized NCG mice engrafted with A549 tumor cells

This study was designed to assess the human immune cell activation by ICVB-1042; inferred by tumor infiltration by human immune cells that may potentially result in therapeutic benefit. This

study was carried out in female CD34+ humanized NOD-*Prkdc*^{em26Cd52}*Il2rg*$^{em26Cd22/}$NjuCrl (huNCG) mice engrafted with human A549 lung carcinoma and treated systemically with ICVB-1042 whereby immune cell infiltration and activation were assessed by flow cytometry. A549 human lung carcinoma cells (5.00E6 cells) were implanted subcutaneously in female CD34+ huNCG mice. At 27 days post tumor implant (designated as Day 0), when the tumor volume reached a mean size of 101 mm^3, the animals were randomized into 2 groups based on tumor burden, humanization rate (defined by human immune cell engraftment), and cord blood donor (equal donor distribution; 6 animals/group). Vehicle or ICVB-1042 was administered intravenously every third day for 3 total administrations. The first and second doses of ICVB-1042 were 1.00E9 PFU, and the third administration was 5.00E8 PFU. Beginning 4 days prior to the first dose and continuing weekly after the initial dose, survival bleeds (~100 µL) were collected from all animals for flow cytometry analysis. Since flow cytometry was the primary end point analysis, ICVB-1042-treated animals were sacrificed when the vehicle group tumor volume neared, but did not exceed, 1000 mm^3. Animals were removed from study due to disease burden; tumor volume was >1000 mm^3, or body weight loss was >20%. All animals were observed for clinical signs at least once daily, and animal survival was monitored throughout the study. At study end, whole blood and tumors were collected for flow cytometry analysis. The concentration of human immune cell subsets was quantified using flow cytometry. Cell counts in the blood were normalized to the volume of whole blood assayed (µL). Cell counts in the tumor were normalized to tumor mass (g) and reported as cells/g. The mean values are reported with SEM calculated (4 animals for each assay timepoint).

Viral Neutralization Assays

Serum samples were thawed and an aliquot of each was heat inactivated at 56ºC for 1 hour. Serum was diluted in PBS to twice the final concentration and combined with an equal volume of 2.5E5 PFU virus in PBS (e.g. 5% serum was combined with virus to test neutralization at 2.5% serum). Virus was incubated with serum for 2 hours at room temperature before transferring reaction volume to 96 well plates containing 2.5E4 A549-Luc2 cells per well to achieve a MOI of 10. For experiments with mouse serum, 2.5E4 PFU virus was used for a MOI of 1. Cells were incubated 4-5 days following addition of virus then cell viability was assessed by measuring luciferase activity. Percent neutralization was quantified as the percent viable cells relative to the no-virus controls.

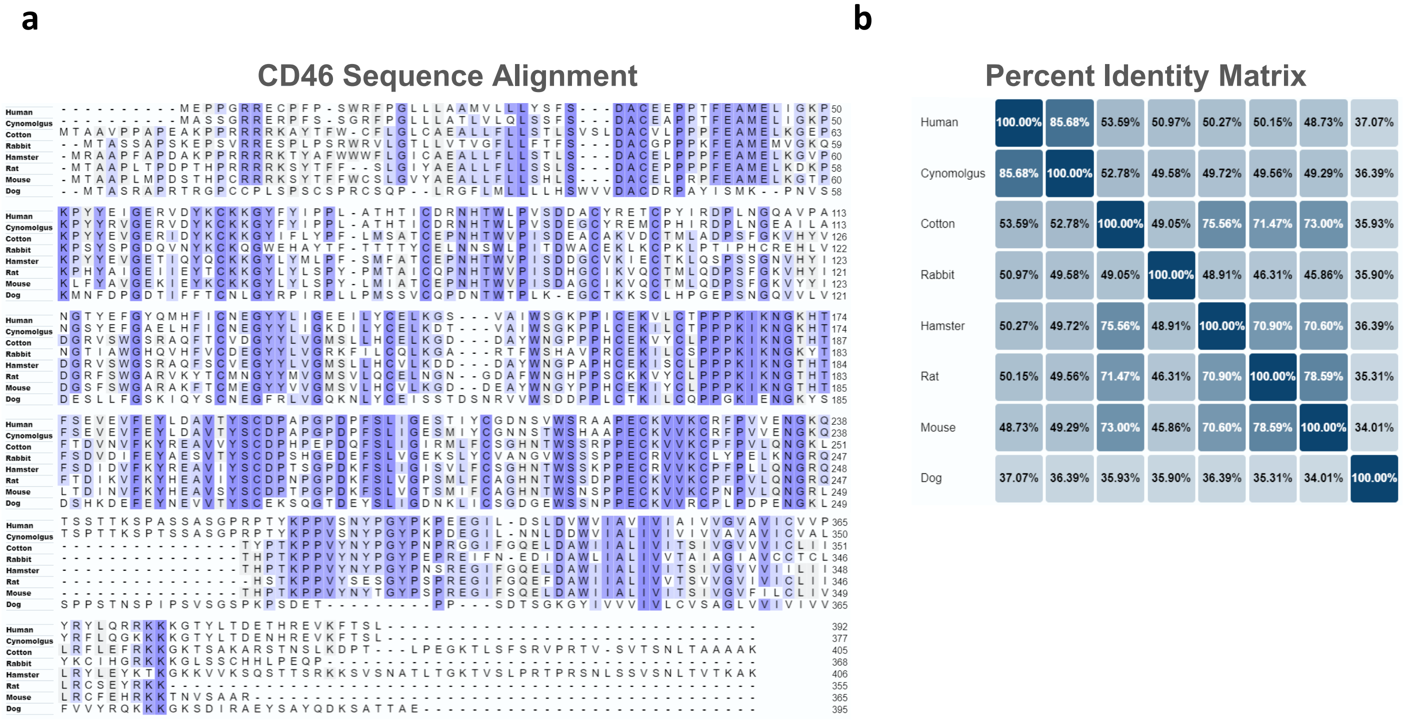

Supplemental Figure 1. CD46 homology across human and non-human species. (**a**) CD46 amino acid sequences from human and non-human species were aligned. (**b**) Percent identity matrix. Sequence alignment was performed using clustalo version 1.2.4. Human = Homo sapiens (Uniport #P15529). Cynomolgus = Macaca fascicularis (Uniport #A0A2K5WCL8). Rabbit = Oryctolagus cuniculus (Uniport #U3KP03). Dog = Canis lupus (Uniport #A0A8C0YZN5). Hamster = Mesocricetus auratus (Uniport #A0A1U8CRM3). Cotton = Sigmodon hispidus (Uniport #A0A977P3R8). Rat = Rattus norvegicus (Uniport #Q9Z0M4).

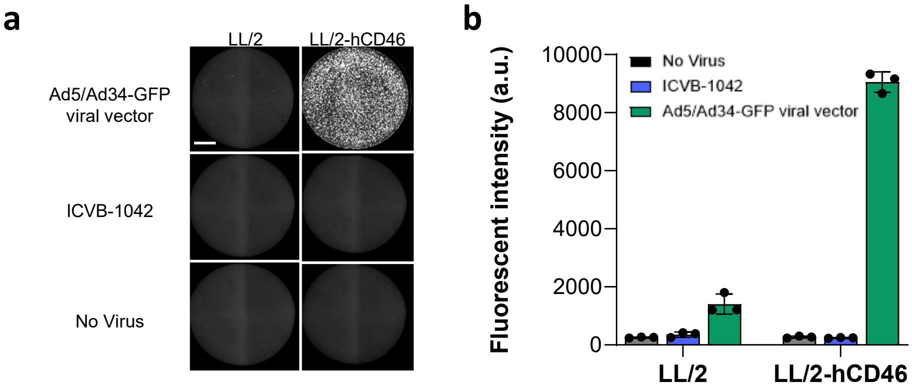

Supplemental Figure 2. Infection of Mouse LL/2 Cells Expressing Human CD46 with ICVB-1042 and Ad5/Ad34-GFP viral vector. (**a**) LL/2 and LL/2-hCD46 cell lines plated at 15,000 cells per well were infected with Ad5/Ad34-GFP viral vector or ICVB-1042 at MOI of 100 or left uninfected ("No Virus"). After 24-hour incubation, YPet or GFP-expressing cells were visualized. Entire well reconstructed images are shown for each condition. Scale bar = 1 mm. (**b**) Average pixel intensities above background were determined from images in (a) and represented as mean values showing individual data points (n=3) with error bars representing standard deviation.

Incubation with ICVB-1042 did not lead to detectable YPet expression in LL/2-huCD46 cells or LL/2 cells. In contrast, incubation with Ad5/Ad34-GFP viral vector led to GFP expression in the LL/2-huCD46 cells but not in LL/2 cells, indicating that while the expression of huCD46 expression is sufficient for the chimeric Ad5/Ad34 fiber-mediated entry into mouse cells, huCD46-expressing mouse cells are still unable to support replication of ICVB-1042.

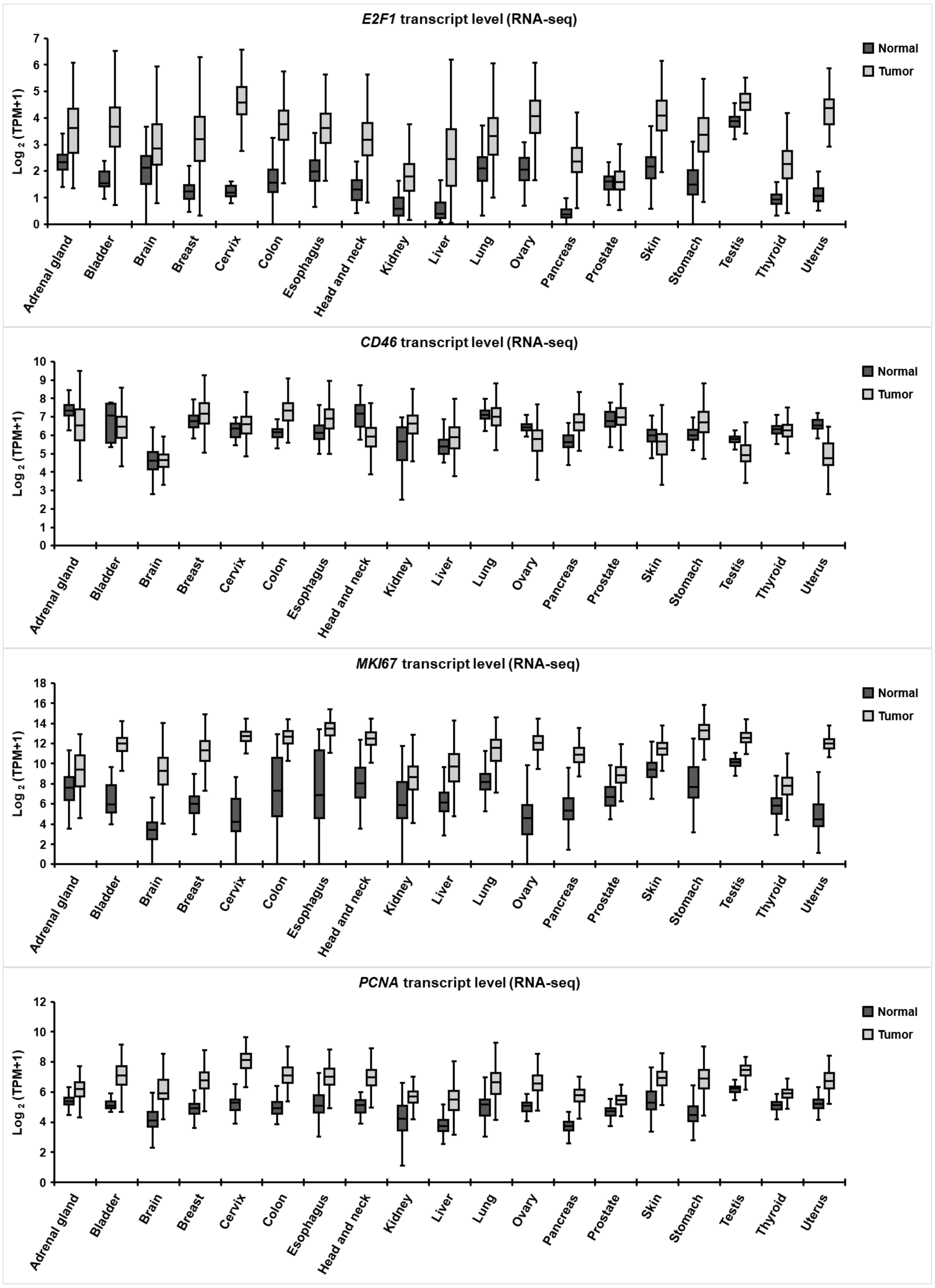

Supplemental Figure 3. Transcript Levels of *E2F1*, *MKI67*, *PCNA* and *CD46* in normal and tumor tissues in vivo based on GTEx and TCGA RNA-seq datasets. Sample size was variable between 9 to 1212. *E2F1* transcript levels were significantly higher in tumor than in normal across all tissue types ($P < 1E\text{-}7$, t-test) except for prostate. In prostate, *E2F1* transcript level was slightly higher in normal than in tumor. *MKI67* transcript levels were significantly higher in tumor than in normal across all tissue types ($P < 1E\text{-}4$, t-test). *PCNA* transcript levels were significantly higher in tumor than in normal across all tissue types ($P < 1E\text{-}5$, t-test). TPM = Transcript per million.

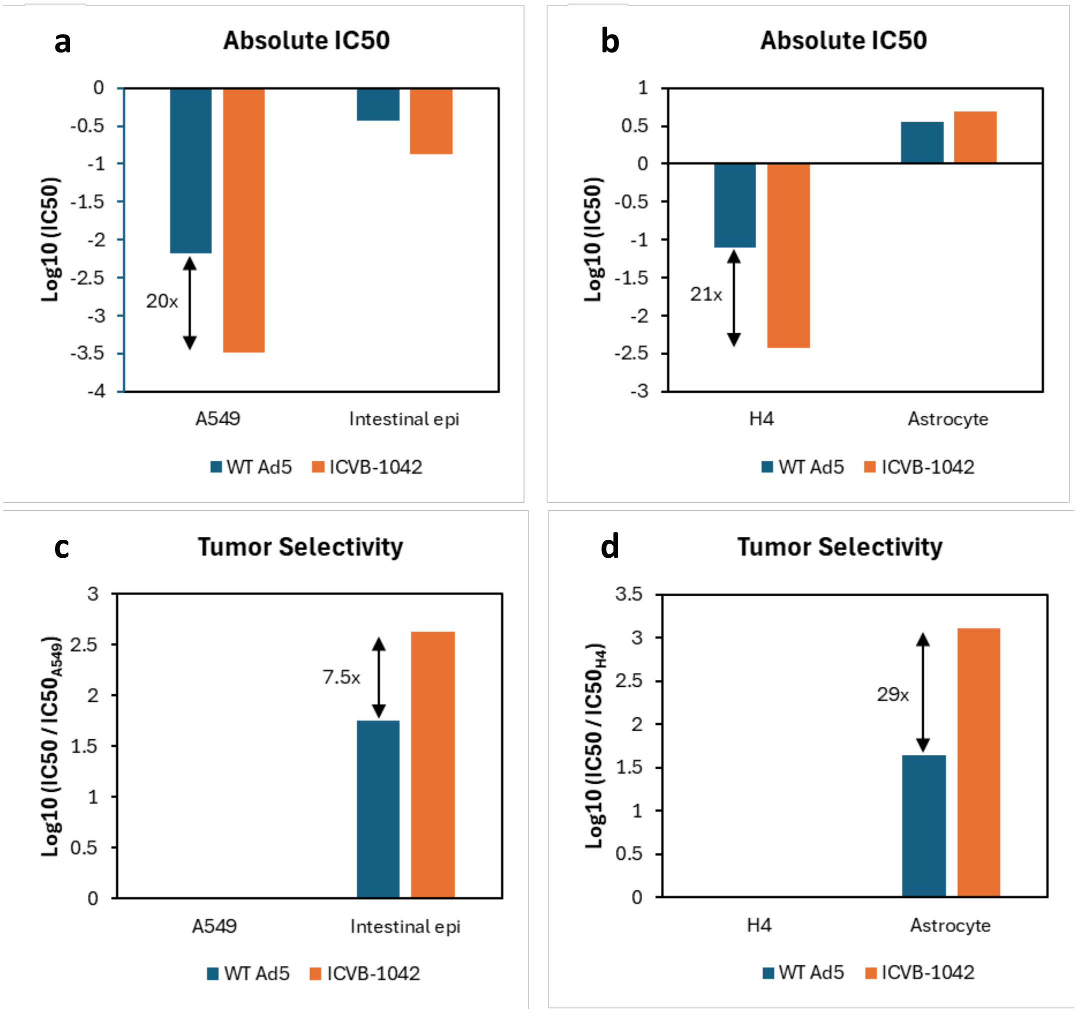

Supplemental Figure 4. Potency and selectivity of ICVB-1042 relative to Wt Ad5. ICVB-1042 is more potent relative to Wt Ad5 in both A549 (Figure a) and H4 (Figure b) cancer cell lines, showing 20- and 21-times potency, respectively. ICVB-1042 also demonstrates improved relative tumor selectivity compared to Wt Ad5, eg, A549 cell line vs Intestinal epithelial primary cells (7.5x selectivity) (Figure c) and H4 cell line versus primary astrocytes (29x selectivity) (Figure d). IC50 was measured by WST-1 cell viability assay. Indicated viruses were serially diluted (5-fold 8 dilutions or 4-fold 9 dilutions) and infected on indicated cancer cell lines and primary cells followed by WST-1 measurement 6 days post infection. Cell culture was maintained with daily partial media change. Dose-response kill curves were fitted to 3 or 4 technical replicate data points per dose. At least 2 independent experiments were conducted, and similar results were observed.

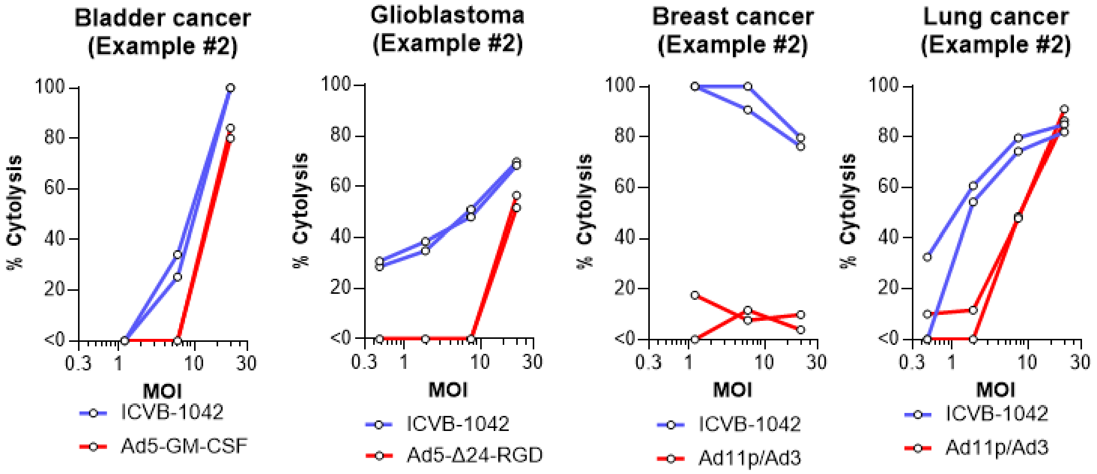

Supplemental Figure 5. The lytic potency of ICVB-1042 in dissociated tumor cells. ICVB-1042 was compared to Ad5-Δ24-RGD (a similar AdOV has been tested in glioblastoma patients), Ad11p/Ad3 (a similar AdOV has been tested in colorectal and non-small cell lung and colorectal cancer patients), and Ad5-GM-CSF (a similar Ad OV has been tested in bladder cancer patients). Data shown are results from one donor per indication, n=2 replicas. Related to Figure 3C.

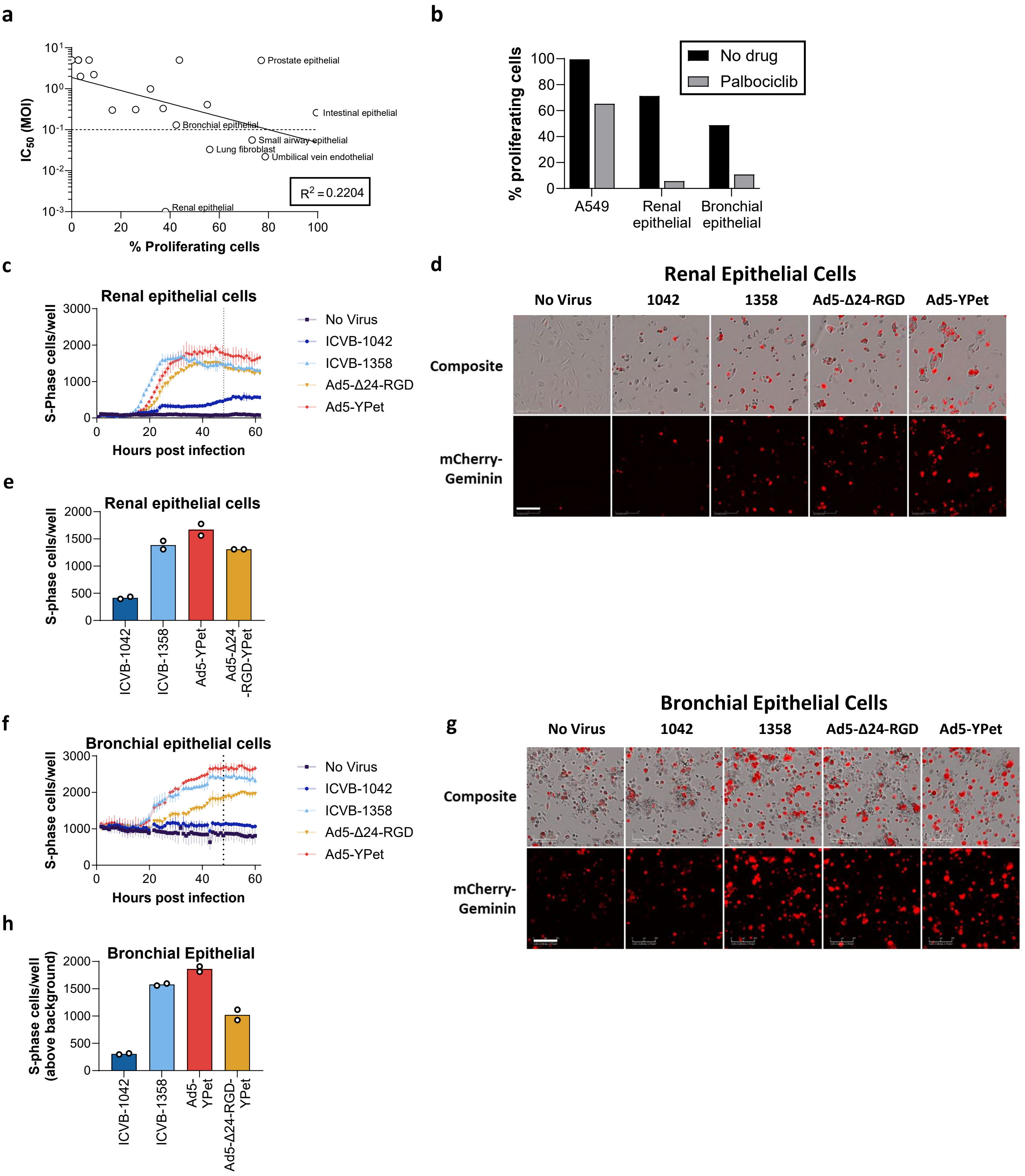

a
IC50 (MOI)
Prostate epithelial
Intestinal epithelial
Bronchial epithelial
Small airway epithelial
Lung fibroblast
Umbilical vein endothelial
Renal epithelial
R2 = 0.2204
% Proliferating cells
b
% proliferating cells
No drug
Palbociclib
A549
Renal epithelial
Bronchial epithelial
c
Renal epithelial cells
S-Phase cells/well
Hours post infection
No Virus
ICVB-1042
ICVB-1358
Ad5-Δ24-RGD
Ad5-YPet
d
Renal Epithelial Cells
No Virus
1042
1358
Ad5-Δ24-RGD
Ad5-YPet
Composite
mCherry-Geminin
e
Renal epithelial cells
S-phase cells/well
ICVB-1042
ICVB-1358
Ad5-YPet
Ad5-Δ24-RGD-YPet
f
Bronchial epithelial cells
S-phase cells/well
Hours post infection
No Virus
ICVB-1042
ICVB-1358
Ad5-Δ24-RGD
Ad5-YPet
g
Bronchial Epithelial Cells
No Virus
1042
1358
Ad5-Δ24-RGD
Ad5-YPet
Composite
mCherry-Geminin
h
Bronchial Epithelial
S-phase cells/well (above background)
ICVB-1042
ICVB-1358
Ad5-YPet
Ad5-Δ24-RGD-YPet

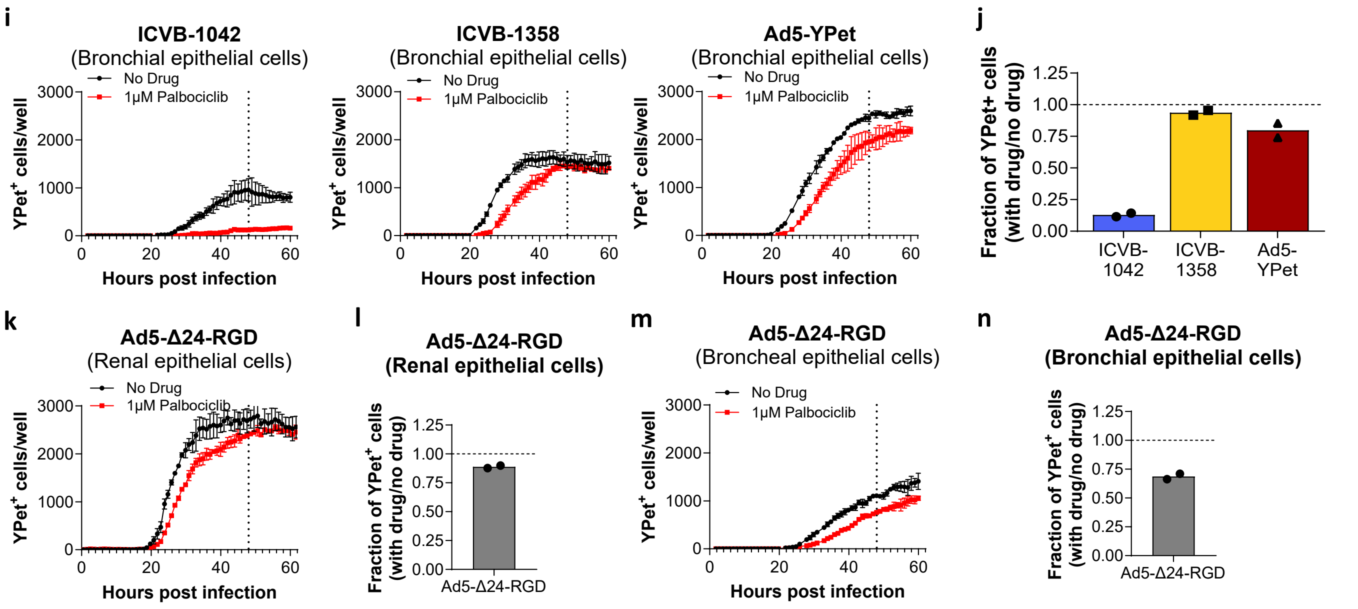

Supplemental Figure 6. Mechanistic studies of ICVB-1042 tumor selectivity.

(a) IC_{50} MOIs of ICVB-1042 and percentage of proliferating cells were determined for 19 primary normal cells types from 10 different tissue origins).

(**b**) Effects of palbociclib treatment on cell proliferation was evaluated for A549, renal epithelial cells (HREs), and bronchial epithelial cells (BrECs) were assessed.

(**c-e**) Primary normal HREs expressing mCherry-geminin were infected with ICVB-1042, ICVB-1358 ($E1A^{WT}$ and $E4orf6/7^{WT}$), Ad5-YPet, and Ad5-Δ24-RGD-YPet at MOI of 10 or left uninfected in the presence of 1μM palbociclib. Cells positive for mCherry-geminin expression were tracked over time to determine cells entering S-phase. Results of HRE. (c) Longitudinal analysis of the number of S-phase cells per well (mean ± SD) revealed ICVB-1042 did not robustly induce cell cycle progression of renal epithelial cells in the presence of palbociclib. (d) Representative images of HREs at 48 hours post-infection, red = mCherry. Scale bar = 200 μm. (e) S-phase cells at 48 hours (n=2 replicates).

(**f-h**) Primary BrECs expressing mCherry-geminin were infected with ICVB-1042, ICVB-1358 ($E1A^{WT}$ and $E4orf6/7^{WT}$), Ad5-YPet, and Ad5-Δ24-RGD-YPet at MOI of 10 or left uninfected in the presence of 1μM palbociclib. Cells positive for mCherry-geminin expression were tracked over time to determine cells entering S-phase. Results of HRE. (f) Longitudinal analysis of the number of S-phase cells per well revealed ICVB-1042 did not robustly induce cell cycle progression of renal epithelial cells in the presence of palbociclib. (g) Representative images of BrECs at 48 hours, red = mCherry. Scale bar = 200 μm. (h) S-phase cells at 48 hours (n=2 replicates).

(**i** and **j**) Primary normal BrECs were infected with viruses in the presence or absence of 1 μM palbociclib. (i) Longitudinal analysis of YPet+ cells per well (mean ± SD). (j) Fraction of YPet+ cells at 48 hours (n=2 replicates).

(**k-n**) Primary HREs (k and l) or BrECs (m and n) were infected with Ad5-Δ24-RGD-YPet at MOI of 10. (k and n) Longitudinal analysis of YPet+ cells per well (k: mean ± SD). (l and n) Fraction of YPet+ cells at 48 hours (n=2 replicates).

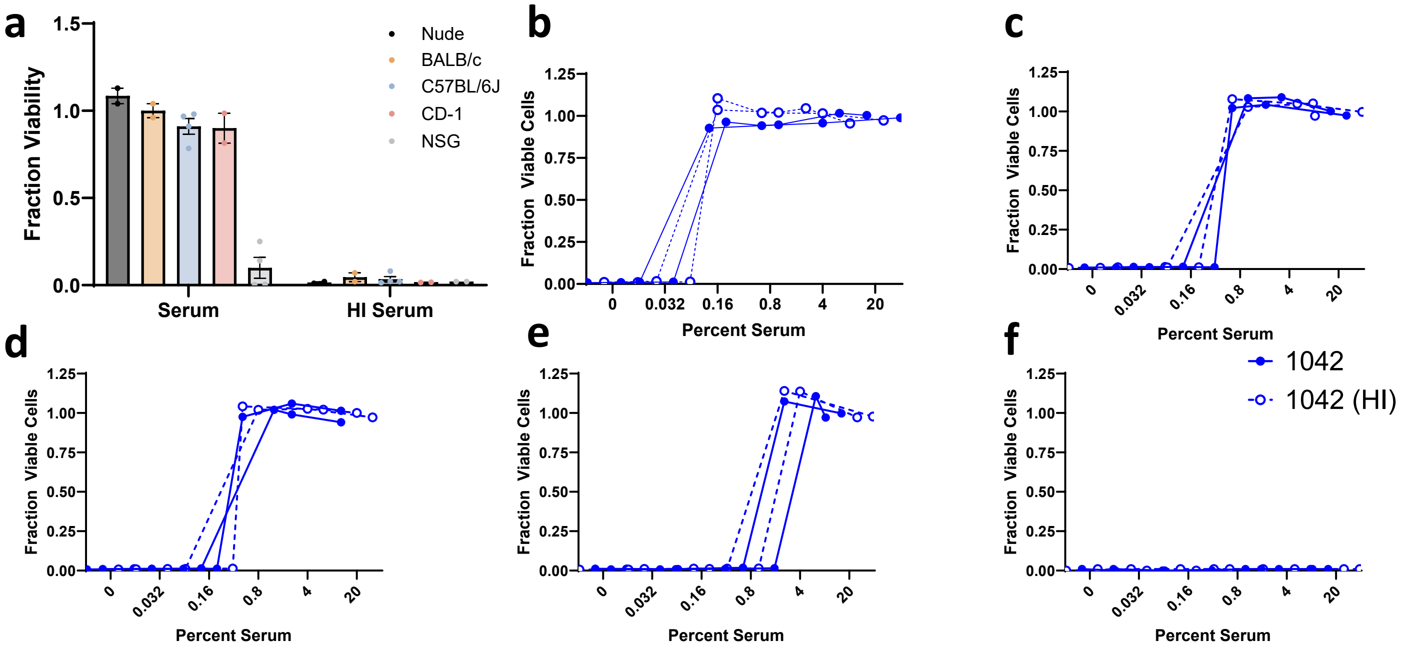

Supplemental Figure 7. a. Mouse serum-mediated neutralization of ICVB-1042. A549-Luc2 cells were infected with ICVB-1042 at a MOI of 1 following pr-incubation of virus with 2.5% mouse serum or heat-inactivated (HI) mouse serum of different strains of mice (Nude [black bar], BALB/c [yellow bar], C57BL/6 [blue bar], CD-1 [red bar], or NSG [grey bar] each with different levels of immune competence) to allow cell-independent viral neutralization to occur. After 4-day incubation, luciferase activity was quantified to determine cell viability relative to control cells that were incubated in the matching mouse serum with no viral input (data not shown). Plot shows fraction of viable cells normalized to no-virus controls in each group (n≥2/group). Error bars represent SD. NSG mice are fully immunodeficient and therefore there is no neutralization of ICVB-1042. **b-f. Neutralization of ICVB-1052 by human serum**. A549-Luc cells were infected with indicated virus at a MOI of 10 following incubation of virus with titrating dilutions of individual human donor sera (± 1 hour heat inactivation at 56° C).

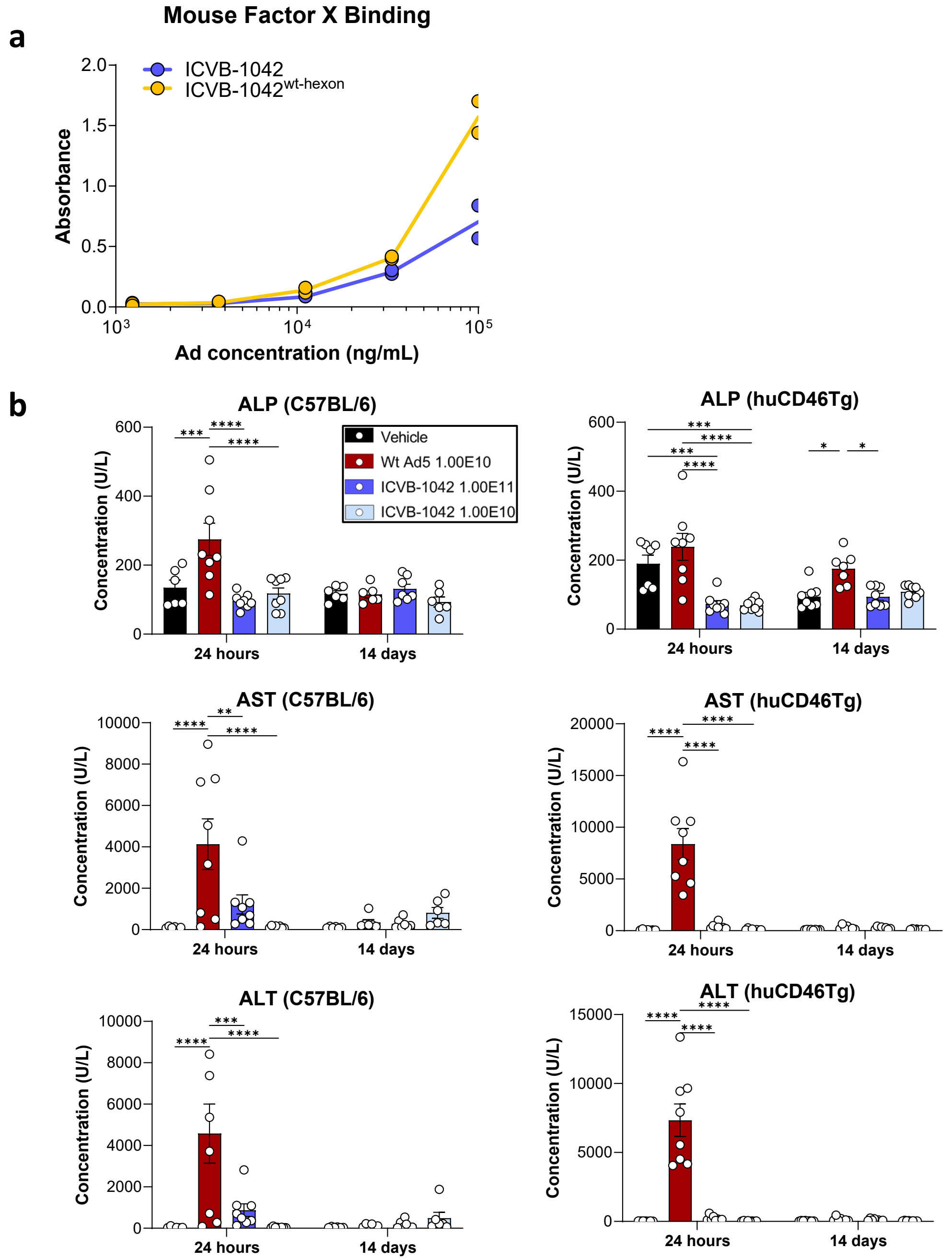

Supplemental Figure 8. Detargeting from Factor X enables systemic administration of ICVB-1042 in mice. (**a**) *In vitro* binding of ICVB-1042 and ICVB-1042$^{wt\text{-}hexon}$ to mouse FX was assessed by ELISA. (**b**) Levels of liver enzymes AST, ALT, and ALP in plasma were monitored in C57BL/6 and CD46tg mice 24 hours and 14 days after IV administration of ICVB-1042 (dark blue, 1E11 VP/dose; light blue, 1E10 VP/dose),Wt Ad5 (red, 1E10 VP/dose), or vehicle (black) in C57BL/6 mice and huCD46Tg mice.

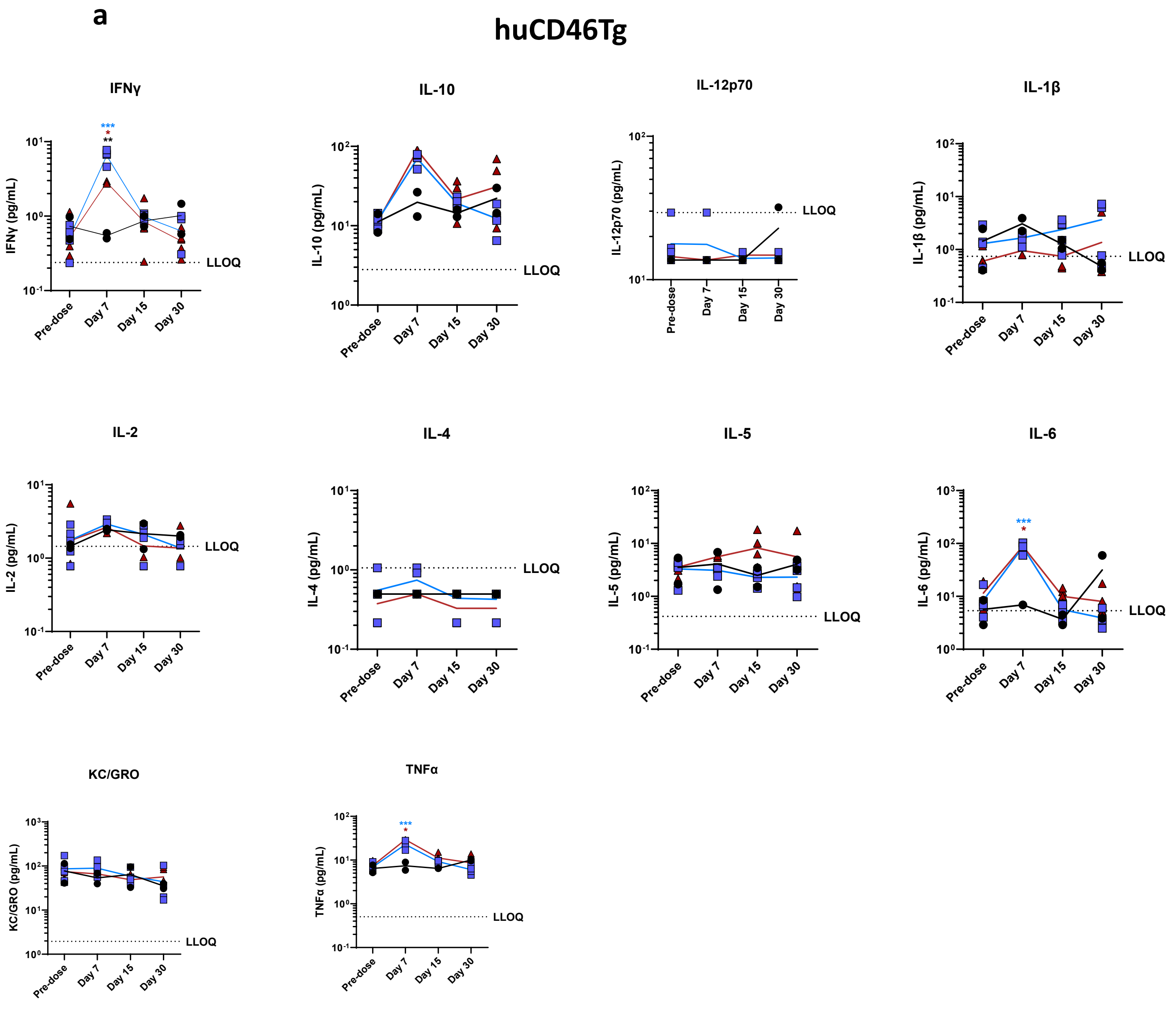
a
huCD46Tg
IFNγ
IFNγ (pg/mL)
IL-10
IL-10 (pg/mL)
IL-12p70
IL-12p70 (pg/mL)
IL-1β
IL-1β (pg/mL)
IL-2
IL-2 (pg/mL)
IL-4
IL-4 (pg/mL)
IL-5
IL-5 (pg/mL)
IL-6
IL-6 (pg/mL)
KC/GRO
KC/GRO (pg/mL)
TNFα
TNFα (pg/mL)
LLOQ
Pre-dose
Day 7
Day 15
Day 30

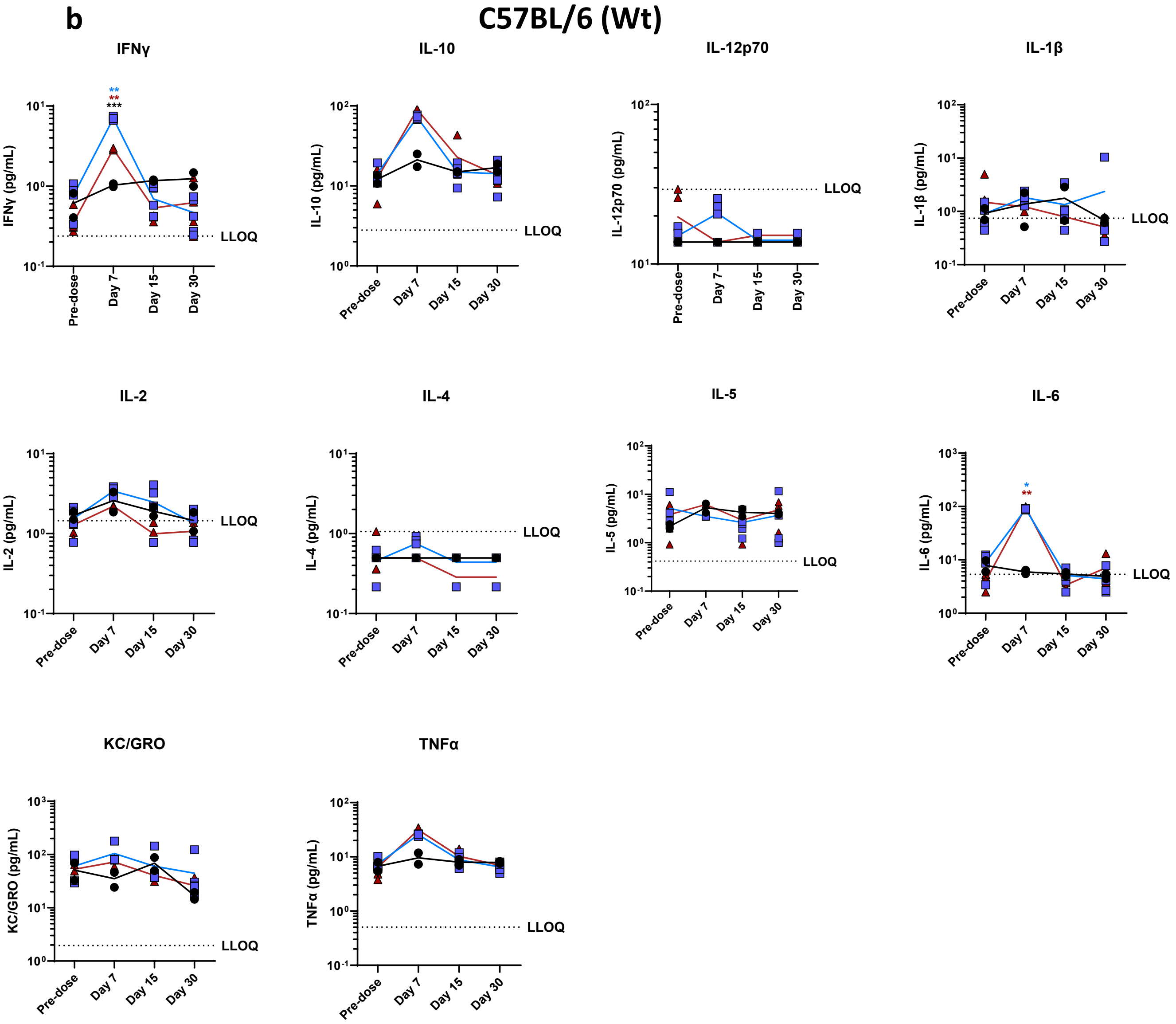

Supplemental Figure 9. Plasma cytokine levels in huCD46Tg and C57BL/6 mice after repeat IV administration of ICVB-1042 versus Wt Ad5. huCD46Tg mice and C57BL/6 mice were treated IV with vehicle (n=2), ICVB-1042 (5.00E9-2.00E10 VP, n=5) or Wt Ad5 (5.00E9-2.00E10 VP, n=7) on Days 0, 3, and 6. Plasma samples were collected pre-dosing and at Day 7, Day 15, and Day 30. Proinflammatory cytokines (IFNγ, IL-1β, IL-2, IL-4, IL-5, IL-6, CXCL1, IL-10, IL-12p70 and TNFα) levels in plasma samples were determined using the Meso Scale Discovery platform. (**a**) Plasma concentration of proinflammatory cytokines over 30 days in huCD46Tg mice. **(b)** Plasma concentration of proinflammatory cytokines over 30 days in Wt mice. The raw data obtained from the assay are plotted on the graphs as the geometric mean. The horizontal dotted line represents the lower limit of quantification (LLOQ). The LLOQ for each cytokine was the lowest calculable detection limit, defined by the assay, multiplied by the dilution factor of the most diluted plasma sample analyzed in the experiment. Data points below the LLOQ were represented on the graph as the LLOQ of the cytokine. The log-transformed values of the data depicted in the figure were analyzed by a mixed-model ANOVA with a Geisser-Greenhouse correction; *p <0.05; **p <0.01; ***p <0.001; ****p <0.0001.

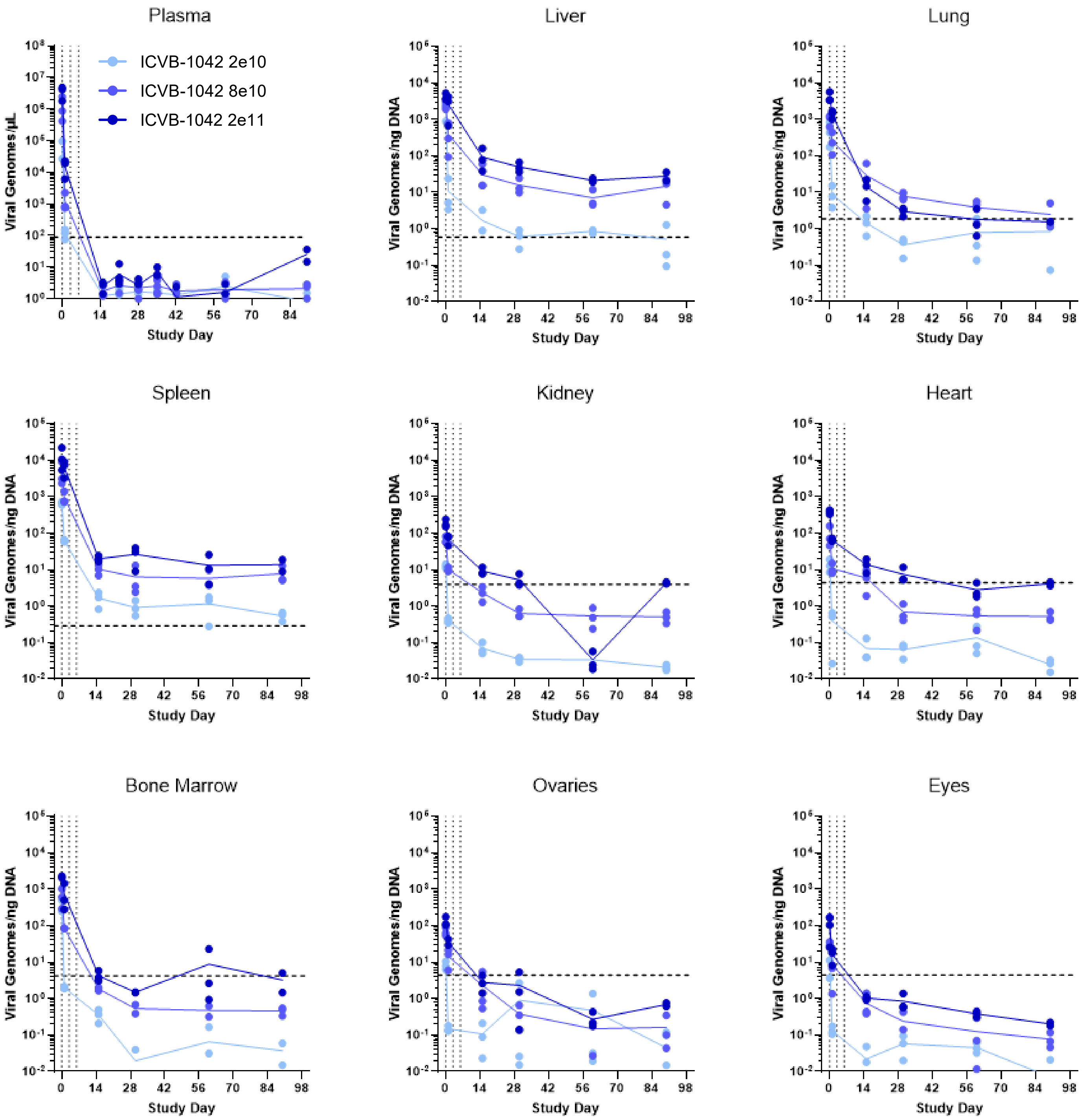
Plasma
Liver
Lung
Spleen
Kidney
Heart
Bone Marrow
Ovaries
Eyes
ICVB-1042 2e10
ICVB-1042 8e10
ICVB-1042 2e11
Viral Genomes/µL
Viral Genomes/ng DNA
Study Day

Supplemental Figure 10. PK and Biodistribution in CD-1 mice following three administrations of ICVB-1042. Twenty-four CD-1 mice per group were intravenously administered ICVB-1042 at 2.00E10 VP, 8.00E10 VP or 2.00E11 VP in 100 µL once every 3 days (Day 0, 3, and 6 indicated by the vertical dotted lines) for up to 3 doses. Solid blue lines present the mean of each group. The biodistribution of ICVB-1042 in plasma and tissues was assessed ex vivo by quantifying viral genomes in plasma, liver, spleen, brain, heart, lungs, bone marrow of femur, kidneys, eyes and ovaries from samples that were collected at 1 hour and 24 hours after the first administration and on Days 15, 60, and 90. ICVB-1042 viral genomes were determined using ddPCR, LLQ for each matrix is shown by the horizontal dashed line. ICVB-1042 viral genomes were detected at all dose levels 1 hour and 24 hours post dose, but by 15 days post dose, all levels of viral genomes fell 13-fold less in the liver and 38- to 239-fold less in the spleen than at 24 hours post initial dose. Viral genomes were detected above LLQ in lung (LLQ = 1.86E0) at the 2 highest doses throughout the course of the study. Viral genomes below the LLQ (88.8 viral genomes/µL) and were not detected for any of the remaining time points. Vehicle animal samples were analyzed alongside treatment animals. All plasma samples from all the animals tested were below LLQ. ICVB-1042 was detected, in a dose-dependent manner, at 1 hour and 24 hours post initial dose in tissue samples. The mean viral kinetics of ICVB-1042 in tissues are shown. Detected viral genomes in the expected target organs were 6- to were 9- to 99-fold less than at 24 hours post initial dose. Viral genomes were detected at low levels above LLQ in bone marrow (LLQ = 4.15E0), heart (LLQ = 4.35E0), and kidney (LLQ = 3.92E0) only at the highest dose at later timepoints. Viral genomes were not detected above LLQ in brain (LLQ = 4.27E0), eye (LLQ = 4.39E0), or ovary (LLQ = 4.35E0) at any dose after the initial 24 hours. Vehicle animal samples were analyzed alongside treatment animals, all tissues from all the animals tested were below LLQ.

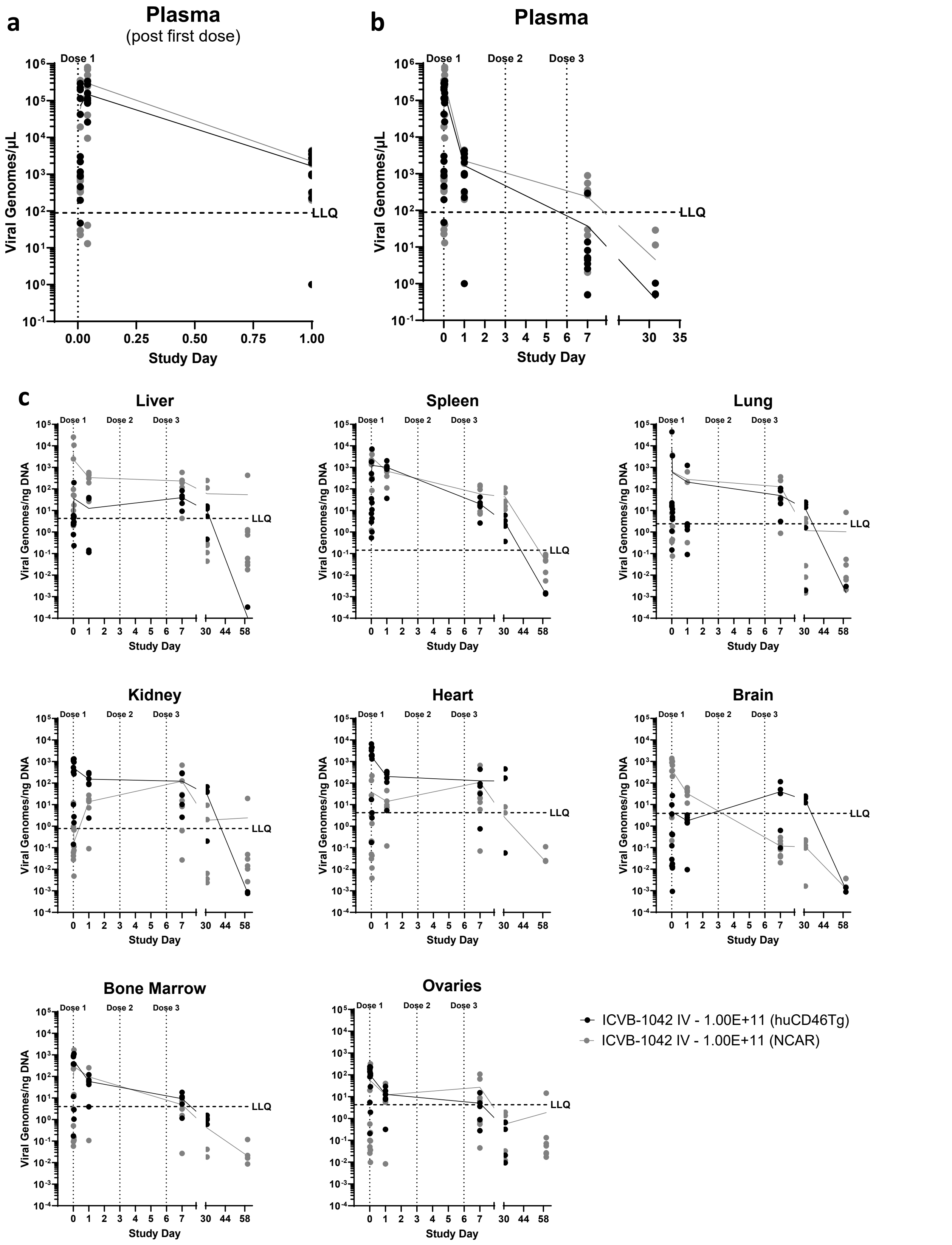

Supplemental Figure 11. Pharmacokinetics and biodistribution of ICVB-1042 in huCD46Tg mice and C57BL/6. ICVB-1042 (1.00E11 VP/dose) was injected IV into huCD46Tg mice and C57BL/6 mice on day 0, 3, and 6 and ICVB-1042 genome copy numbers in plasma and various tissues were determined using ddPCR. Solid lines represent the mean. (**a**) The kinetic of CVB-1042 genome copy numbers in plasma in the first 24 hours after the first dose (**b**) The kinetic of ICVB-1042 genome copy numbers in plasma over 31 days. (**c**) The kinetic of ICVB-1042 viral copy number in liver, spleen, lung, kidney, heart, brain, bone marrow, and ovaries. Vertical dotted lines represent each dose.
Horizontal dotted lines represent lower limit of quantification (LLQ).

The difference in ICVB-1042 biodistribution between wild-type C57BL/6 mice and hCD46Tg mice was varied and dependent on tissue type. Comparing the two strains, similar levels of ICVB-1042 was detected in the spleen, lungs, bone marrow and ovaries throughout the study. In these tissues, there was less than a 6-fold difference in detected ICVB-1042 at any time, except the spleen and lung tissues at Day 31. At this timepoint, ICVB-1042 in the spleen was 16-fold greater in the hCD46tg animals compared to the wild-type C57BL/6, and below LLQ in the lungs of the wild-type animals while still within the quantifiable range in the hCD46Tg. In both strains, ICVB-1042 was below LLQ or undetected by Day 60 in these tissues.

Biodistribution of ICVB-1042 to the liver and brain was decreased in the hCD46Tg mice compared to the wild type C57BL/6 at the 15 minutes, 1-, and 24-hour post-initial dose timepoints. In the liver, ICVB-1042 levels were lower in the hCD46Tg, compared to the wild-type mice, with 1389-fold lower levels detected at 15 minutes, 62-fold lower at 1 hour post initial dose, 27-fold lower at 24 hours. By Day 60 ICVB-1042 in the liver was below LLQ for the hCD46tg, while still in the quantifiable range for the wild-type. Similarly, detected ICVB-1042 in the brain was also lower in hCD46Tg compared to wild-type mice after dosing. In hCD46Tg mice, detected ICVB-1042 was below LLQ at 15 minutes, 1 hour, 24 hours post initial dose. However, in the wild-type mice, ICVB-1042 was detected at 647.62 viral genomes/ng, 374.80 viral genomes/ng, and 32.48 viral genomes/ng respectively. By Day 60 ICVB-1042 in the brain was below LLQ for both groups.

Biodistribution of ICVB-1042 to the kidney and heart was increased in the hCD46Tg mice compared to the wild type C57BL/6 at the 15 minutes, 1-, and 24-hour post-initial dose timepoints. In the kidneys of the huCD46Tg mice, viral genomes were 206-fold greater at 15 minutes post initial dose, 2692-fold greater at 1 hour, 11-fold greater at 24 hours, compared to the wild type group. In the heart of the huCD46Tg mice, viral genomes were 98-fold greater at 15 minutes post initial dose, 44-fold greater at 1 hour post initial dose, 14-fold greater at 24 hours. In both treatment groups for both the kidney and heart, detectable viral genomes were equal by Day 7 and below the LLQ at Day 60.

There was a minimal difference in ICVB-1042 plasma pharmacokinetics between wild type C57BL/6 and hCD46Tg mice throughout the study. In both models ICVB-1042 is detected at the 15 minutes, 1-, and 24-hour post-initial dose timepoints, the fold difference in viral genome concentration in the plasma of the huCD46Tg mice compared to the wild-type C57BL/6 ranged from 0.50-1.21. By 7 days post initial dose, viral genomes were detected in both groups, however in the hCD46Tg mice, were below the lower limit of quantification. After the Day 7 timepoint, ICVB-1042 was below LLQ in both groups and were not detectable by Day 60. Plasma ICVB-1042 viral genomes were BLQ by day 7 in both transgenic and control mice. All tissues were BLQ by day 60 except the liver in transgenic mice only. In addition, it is important to note that the viral genomes detected do not directly correspond to replication-competent virions. As expected, no indication of ongoing viral replication was observed post dosing. Based on the biodistribution and pharmacokinetic data, the expression of huCD46 in the mouse model transiently impacted biodistribution of ICVB-1042 in some tissues but did not impact viral kinetics in plasma. Increased accumulation of virus in the lung and reduced accumulation in the liver in huCD46Tg mice compared to control mice has been reported for other cluster of differentiation 46 (CD46) tropic viruses administered IV (Stone 2005, Verhaagh 2006).

Stone D, Ni S, Li ZY, et al. Development and assessment of human adenovirus type 11 as a gene transfer vector. *J Virol.* 2005;79(8):5090-104. doi: 10.1128/JVI.79.8.5090-5104.2005
Verhaagh S, de Jong E, Goudsmit Jet al. Human CD46-transgenic mice in studies involving replication-incompetent adenoviral type 35 vectors. J Gen Virol. 2006;87(Pt 2):255-65. doi: 10.1099/vir.0.81293-0.

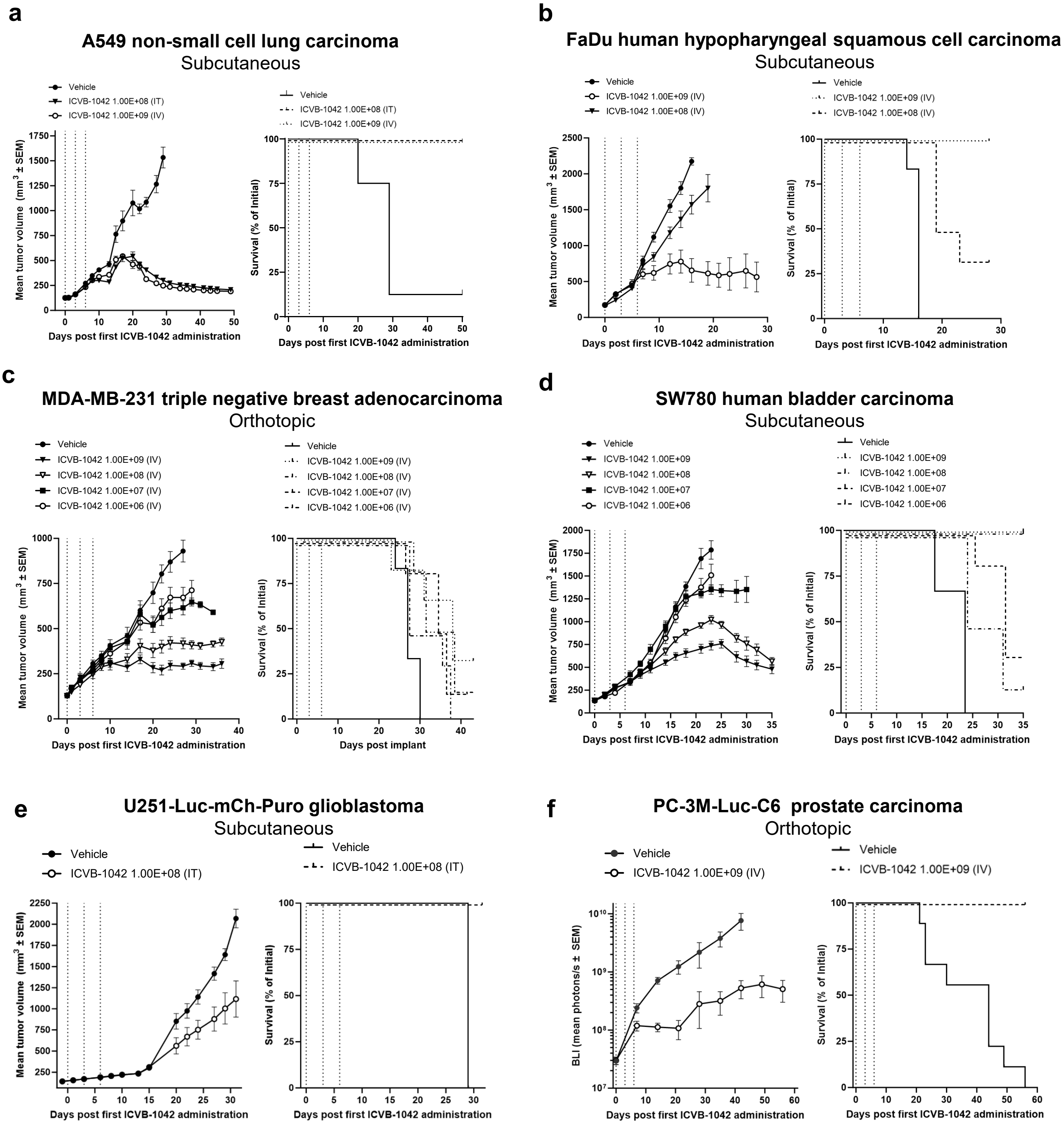
a
A549 non-small cell lung carcinoma
Subcutaneous
Vehicle
ICVB-1042 1.00E+08 (IT)
ICVB-1042 1.00E+09 (IV)
Mean tumor volume (mm³ ± SEM)
Survival (% of Initial)
Days post first ICVB-1042 administration
b
FaDu human hypopharyngeal squamous cell carcinoma
Subcutaneous
Vehicle
ICVB-1042 1.00E+09 (IV)
ICVB-1042 1.00E+08 (IV)
Mean tumor volume (mm³ ± SEM)
Survival (% of Initial)
Days post first ICVB-1042 administration
c
MDA-MB-231 triple negative breast adenocarcinoma
Orthotopic
Vehicle
ICVB-1042 1.00E+09 (IV)
ICVB-1042 1.00E+08 (IV)
ICVB-1042 1.00E+07 (IV)
ICVB-1042 1.00E+06 (IV)
Mean tumor volume (mm³ ± SEM)
Survival (% of Initial)
Days post first ICVB-1042 administration
Days post implant
d
SW780 human bladder carcinoma
Subcutaneous
Vehicle
ICVB-1042 1.00E+09
ICVB-1042 1.00E+08
ICVB-1042 1.00E+07
ICVB-1042 1.00E+06
Mean tumor volume (mm³ ± SEM)
Survival (% of Initial)
Days post first ICVB-1042 administration
e
U251-Luc-mCh-Puro glioblastoma
Subcutaneous
Vehicle
ICVB-1042 1.00E+08 (IT)
Mean tumor volume (mm³ ± SEM)
Survival (% of Initial)
Days post first ICVB-1042 administration
f
PC-3M-Luc-C6 prostate carcinoma
Orthotopic
Vehicle
ICVB-1042 1.00E+09 (IV)
BLI (mean photons/s ± SEM)
Survival (% of Initial)
Days post first ICVB-1042 administration

Supplemental Figure 12. In vivo efficacy of ICVB-1042 in various human tumor xenograft models. For all tumor xenograft models, the mean tumor size (left) and survival of animals (right) were tracked over time. **(a**) Subcutaneously (SC) implanted A549 non-small cell lung adenocarcinoma in female NSG mice (n=8). Disease progression and response to therapy was assessed by caliper measurement of the tumor. Anti-tumor efficacy was evident with ICVB-1042 administrated via IV and IT. IV administration of 1.00E+09 PFU/injection of ICVB-1042 produced a Day 29 Median ΔT/ΔC of 7.6%. The IT administration of 1.00E+08 pfu/injection produced a Day 29 Median ΔT/ΔC of 14%. ICVB-1042 treatment resulted in 100% overall survival and inhibition of tumor growth progression. Day 29 was chosen for ΔT/ΔC evaluation since it was the last day at least 75% of control mice remained on study. (**b)** SC implanted FaDu human hypopharyngeal squamous cell carcinoma in female NSG mice (n=6). Disease progression and response to therapy was assessed by caliper measurement of the tumor. Treatment with ICVB-1042 at 1.00E+09 PFU or 1.00E+08 produced statistically significant and dose-dependent anti-cancer activity. Treatment with 1.00E+09 PFU IV resulted in a Day 14 median ΔT/ΔC of 34% ($p<0.001$). ANOVA test followed by a post-hoc pairwise comparison by the method of Holm-Sidak). Although statistically significant, treatment with 1.00E+08 PFU produced limited anti-cancer activity, resulting in a Day 14 median ΔT/ΔC of 79% ($p=0.02$). By scheduled study end (Day 28) treatment with ICVB-1042 at 1.00E9 resulted in 100% overall survival, whereas treatment with 1.00E8 resulted in an overall survival of 34%. All control animals were euthanized by Day 16. Day 14 was chosen for ΔT/ΔC evaluation since it was the last day all control mice remained on study. (**c)** Orthotopically implanted MDA-MB-231 triple negative breast carcinoma in female NSG mice (n=6). Disease progression and response to therapy was assessed by caliper measurement of the tumor. Treatment with ICVB-1042 at 1.00E+09 PFU or 1.00E+08 produced statistically significant and dose-dependent anti-cancer activity; these two dose levels are presented in the table in Figure 5 whereas all four dose levels are shown here. Treatment with 1.00E+09 produced a median ΔT/ΔC of 21% (p value: <0.001; non-parametric ANOVA test followed by a post-hoc pairwise comparison by the method of Holm-Sidak), treatment with 1.00E+08 produced a median ΔT/ΔC of 35% (p value: <0.001). Although treatment at the lower doses (1.00E+07 or 1.00E+06 PFU/animal) produced statistically significant activity compared to the control group, the efficacy was not biologically relevant (median ΔT/ΔC value >42%). Day 24 was chosen for ΔT/ΔC evaluation since it was the last measurement day before any animal was euthanized due to tumor burden. (**d)** SC implanted SW780 human bladder carcinoma in female NSG mice (n=6). Administration of ICVB-1042 IV at four different dose levels was examined to determine the minimum effective dose in this model. Disease progression and response to therapy was assessed by caliper measurement of the tumor. Treatment with ICVB-1042 produced a dose dependent anti-tumor response. The two efficacious dose levels are represented in the table in Figure 5; all four dose levels are represented here. 1.00E+09 pfu/injection produced a Day Median ΔT/ΔC of 39% ($p<0.001$; non-parametric ANOVA test followed by a post-hoc pairwise comparison by the method of Shapiro-Wilk), 1.00E+08 pfu/injection produced a Median ΔT/ΔC of 60% ($p<0.001$). ICVB-1042 administered at 1.00E+07 and 1.00E+06 PFU did not demonstrate a statistically significant response. Day 18 was chosen for ΔT/ΔC evaluation since it was the last measurement day before any animal was euthanized due to tumor burden. (**e**) SC implanted U251-Luc-mCh-Puro human glioblastoma in female NSG mice (n=6). Intratumoral treatment with ICVB-1042 at 1.00E+08 pfu/injection produced significant anti-cancer activity as compared to the control group with a Day 31 median ΔT/ΔC value of 52% ($p=0.001$, ANOVA test followed by a post-hoc pairwise comparison by the method of Holm-Sidak). Day 31 was chosen as it was the day all median animals remained on study. (**f**) Orthotopically implanted PC-3M-Luc-C6 prostate carcinoma in male NSG mice (n=9). Tumor burden was assessed by bioluminescence imaging (BLI). Bioluminescence imaging (BLI) of luciferase-expressing tumor cell lines enables a noninvasive determination of site-localized tumor burden. The quantity of emitted light from the tumor after systemic injection of D-luciferin is expected to correlate with viable tumor burden. Treatment with ICVB-1042 at 1.00E+09 PFU produced anti-tumor activity with a Day 54 T/C value of 1.8% ($p<0.05$, ANOVA test followed by a post-hoc pairwise comparison by the method of Dunn was used to compare ΔTs and ΔCs between groups on Day 48 the last day the median control mouse was on study).

(a-f) Dotted vertical lines indicate ICVB-1042 administration on Days 0, 3 and 6. Data is excluded in control groups when 50% or less of animals remain on study. Treatment was well tolerated in all models and produced minimal body weight loss and no deaths within the treatment window (2 weeks past the first administration).

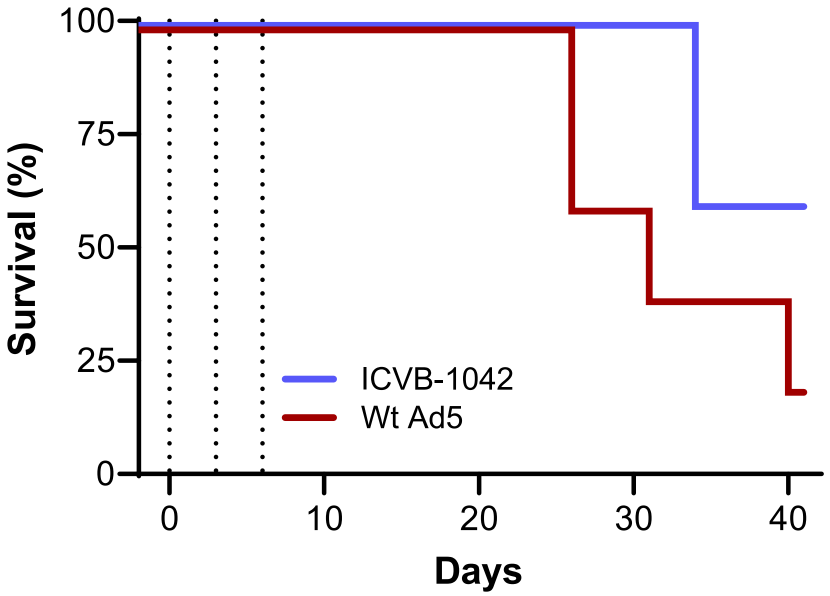

Supplemental Figure 13. Enhanced survival of NSG mice with a triple-negative breast cancer MDA-MB-231 following ICVB-1042 administration IV compared to Wt Ad5 administration IV. NSG mice bearing orthotopic human breast carcinoma tumors (n=5/group) were treated IV with ICVB-1042 (red) or Wt Ad5 (blue) (2.00E8 PFU), on day 0, 3, and 6. Survival was monitored throughout the study and is presented here as days post the first administration of ICVB-1042. ICVB-1042 resulted in increased survival with 60% of the animals remaining on study at study end versus Wt Ad5 treated animals where 20% of animals remained on study. Survival data correlated with tumor growth inhibition and body weight loss.

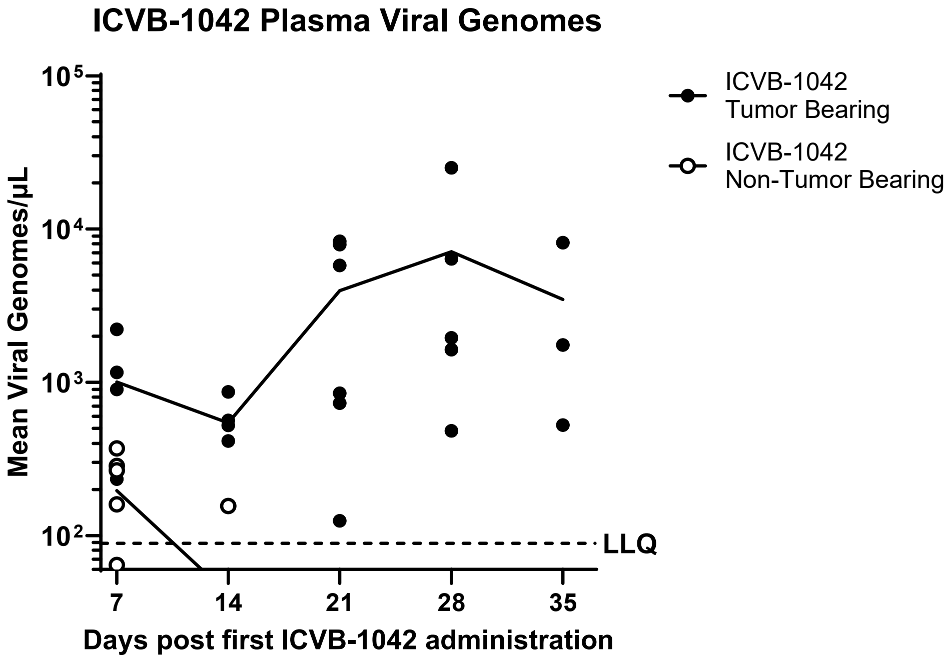

Supplemental Figure 14. Replication of ICVB-1042 in tumor leads to systemic viral dissemination in NSG mice. NSG mice orthotopically implanted MDA-MB-231 triple negative breast carcinoma (black circles) versus NSG mice (no tumor) (white circles) were administered ICVB-1042 at 1.00E9 PFU/dose every third day for a total of 3 administrations (ie. Administration on days 0, 3 and 6). Analysis of ICVB-1042 viral genomes in plasma were assessed by ddPCR. Tumor bearing animals had detectable levels of ICVB-1042 at 24 hours post third administration (Day 7) throughout the course of the sampling (up to 35 days post first administration of ICVB-1042). At Day 14 post the first administration of ICVB-1042 until Day 35 (last sampling timepoint), the level of detectable ICVB-1042 increased in these animals, indicating virus replication in the human tumors as expected. Whereas the non-tumor bearing animals only had detectable genome copies of ICVB-1042 at the 24-hour post third dose administration timepoint since there is no virus replication due to lack of human tumor on board these animals. The solid lines represent the mean of each group. This graph is from the same study as Supplemental Figure 11c. This was also observed in the SW780 model (Supplemental Figure 11d).

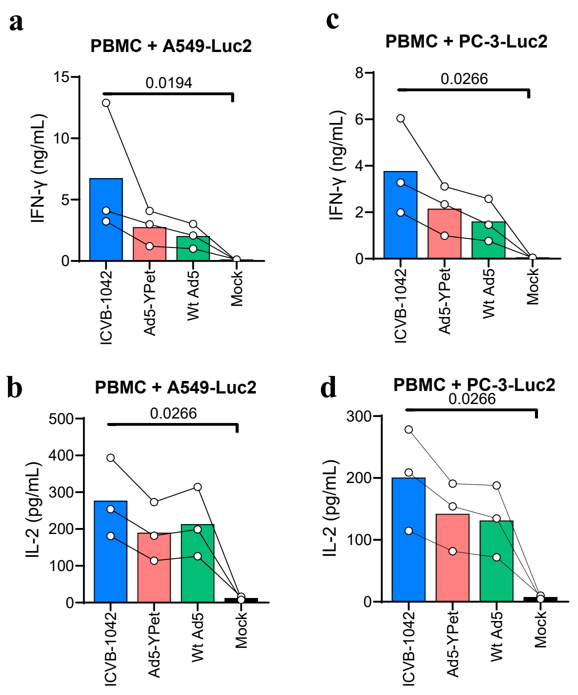

Supplemental Figure 15. Cytokine responses elicited by cancer cell lines infected with ICVB-1042. 20,000 cancer cells per well were infected with ICVB-1042, Ad5-YPet, or Wt Ad5 at MOI=15 or left uninfected (MOI=0, “Mock”). After 24 hours of incubation, cells were cocultured with PBMCs labeled with CTV at the target (cancer cells) to CD3+ T-cell ratio of 1:10 for an additional 24 hours. (a, b) A549-Luc2 cells were infected. (c, d) PC-3-Luc2 cells were infected. (a, c) IFN-g and (b, d) IL-2 concentration in culture supernatants were quantified using the Meso Scale Discovery platform. All assays were performed in duplicate. Each symbol (white circle) represents the average data from a unique donor (n=3 PBMC donors). Each bar represents the mean. Data from the same donor across different viral infection conditions are connected by lines. Statistical analysis was performed using Friedman test followed by Dunn’s post-test.

Supplementary Table 1 Summary of adenoviruses used

Virus	Fiber composition (Receptor)[1]	Reporter	Modifications	Purpose of modification	Additional notes
ICVB-1042 (Clinical Product)	[Ad5]-[Ad34] (CD46)	YPet	$E1A^{\Delta 15bp[122\text{-}LTCHE\text{-}126]}$	Restrict virus replication to cells with high E2F activity	See footnotes for modification references
			ΔE4orf6/7	Restrict virus replication to cells with load DNA damage sensing response	
			$Hexon^{E451Q}$	Evade liver sequestration by the complement Factor X and enable IV dosing.	
			Δ12.5K, ΔCR1a0, Δgp19k, ΔRIDa, ΔRIDb, Δ14.7K	E3 genes deletion to enhance viral cytotoxicity in tumor cells	
			YPet-P2A-ADP	Visualization of viral infection	
			Fiber [5-34]	Chimeric Ad5 fiber carrying theAd34 knob for enhanced tropism	
ICVB-$1042^{wt\text{-}hexon}$	[Ad5]-[Ad34] (CD46)	YPet	Incorporates all modifications present in ICVB-1042, excluding $hexon^{E451Q}$.	As in ICVB-1042.	Engineered to evaluate the effect of (the lack of) $hexon^{E451Q}$ modification
Ad5/34 wtE1A-E4orf6/7	[Ad5]-[Ad34] (CD46)	YPet	$Hexon^{E451Q}$	Evade liver sequestration by the complement Factor X and enable IV dosing	Non-tumor-selective version of ICVB-1042
			Δ12.5K, ΔCR1a0, Δgp19k, ΔRIDa, ΔRIDb, Δ14.7K	E3 genes deletion to potentially enhance viral cytotoxicity in tumor cells	
			YPet-P2A-ADP	Visualization of viral infection	
			Fiber [5-34]	Chimeric Ad5 fiber, Ad34 knob for enhanced tropism	
Wt Ad5	[Ad5]-[Ad5] (CAR)	None	None	N/A	Reference Wt Ad5 with no modifications (American Type Culture Collection; #VR-1516)
Ad5-YPet	[Ad5]-[Ad5] (CAR)	YPet	YPet-P2A-ADP	Visualization of viral infection	Wt Ad5 virus with YPet.
Ad5/Ad34-GFP viral	[Ad5]-[Ad34] (CD46)	Firefly luciferase EGFP	ΔE1A-promoter, DE1A, ΔE1B19K, DE1B55K	E1 genes and promoter deletion to restrict virus replication to cells with exogenous E1A expression	Tumor-selective virus with the same tropism and
			EEF1A1promoter-	Visualization of viral infection. Luciferase and EGFP are	

Supplementary Table 1 Summary of adenoviruses used

Virus	Fiber composition (Receptor)[1]	Reporter	Modifications	Purpose of modification	Additional notes
vector			Luciferase-EGFP	expressed as a fusion peptide under the control of the Human Eukaryotic Elongation Factor 1 a1 (EEF1A1, also known as EF1A) promoter.	liver de-targeting mutation present in ICVB-1042, and expressing the Luciferase reporter
			Hexon^{E451Q}	Evade liver sequestration by the complement Factor X and enable IV dosing	
			Fiber [5-34]	Chimeric Ad5 fiber, Ad34 knob for enhanced tropism	
Ad5-GM-CSF or Ad5-GM-CSF-YPet	[Ad5]-[Ad5] (CAR)	None or YPet	DE1A promoter:: E2F1 promoter	A substitution of the endogenous E1A promoter with the one of the Human E2F1 gene drives expression of the E1 viral genes in cells with high E2F activity	Engineered based on CG0070, which has been clinically tested in patients with bladder cancer
			Dgp19k::HuGM-CSF	Expression of the Human granulocyte macrophage-colony stimulating factor (GM-CSF) is inserted in place of the deleted gp19k gene and transcribed under the control of the endogenous viral E3 promoter for the purpose of generating an increased immune stimulation	
			YPet-P2A-ADP	Visualization of viral infection	
Ad5-Δ24-RGD or Ad5-Δ24-RGD-YPet	[Ad5]-[Ad5-RGD] (CAR, and $\alpha_v\beta_3$ and $\alpha_v\beta_5$ integrins)	None or YPet	E1A$^{D24bp[122-LTCHEAGF-129]}$	Restrict virus replication to cells with high E2F activity	Engineered based on DNX-2401, which has been clinically tested in patients with glioblastoma.
			Fiber^{RGD4C}	The RGD4C motif (CDCRGDCFC) was inserted in the HI loop of the fiber knob to promote cell targeting via CAR-independent interaction through $\alpha_v\beta_3$ and $\alpha_v\beta_5$ integrins	
			YPet-P2A-ADP	Visualization of viral infection	
Ad11p/Ad3 Or Ad11p/Ad3-YPet	[Ad11p]-[Ad11p] (CD46, DSG2)	None or YPet	Chimeric Ad11p/Ad3 genome	Modified based on ColoAd1, which was originally generated through directed evolution	Engineered based on ColoAd1, which has been clinically tested in patients with NSCLC and colorectal cancer.
			15K-P2A-YPet	Visualization of viral infection	

YPet: Inserted to visualize viral infection and replication, **E3 deletion**: Modified to increase viral cytotoxicity[26] and host immune evasion[27-32], **Fiber**: Enhance viral tropism by binding ubiquitous CD46 for cell entry[10], **E1A + E4orf6/7**: Restrict virus to host cells with high E2F activity[1-4], **Hexon**: Modified to avoid Factor X-mediated liver sequestration[17,18].

Ad3=adenovirus serotype 3; Ad5=adenovirus serotype 5; Ad11p=adenovirus serotype 11p; Ad34=adenovirus serotype 34; CAR=coxsackie adenovirus receptor; CD46=cluster of differentiation 46; DSG2=desmoglein-2; ::=substitution; EEF1A1=Human Eukaryotic Elongation Factor 1 α1; EGFP=enhanced green fluorescent protein; huGM-CSF=human granulocyte macrophage-colony stimulating factor; N/A=not applicable; NSCLC=non-small cell lung cancer; OV=oncolytic virus; Wt Ad5=wild type adenovirus serotype 5; YPet=yellow fluorescent reporter protein.

[1] [tail/shaft]-[knob]

Supplementary Table 2. IC_{50} values from WST-1 assay used to assess cytotoxicity of ICVB-1042 and comparator Ads in nonhuman primary cells and cell lines

Species	Primary or Cell line	Cell name	Tissue	Wt Ad5 IC50 (MOI)	ICVB-874 IC50 (MOI)	ICVB-1041 IC50 (MOI)	ICVB-1042 IC50 (MOI)
Human	Cell Line	A549	Lung	3.249	0.862	0.96	0.986
Mouse	Cell Line	CMT-93	Rectum	>15	>15	>15	>15
		JC	Breast	>15	>15	>15	>15
		LL/2	Lung	>15	>15	>15	>15
		LL/2-hCD46	Lung	>15	>15	>15	>15
	Primary	CD-1053	Alveolar	>15	>15	>15	>15
		CD-1034	Kidney	>15	>15	>15	>15
Rat	Cell Line	L2	Lung	>15	>15	>15	>15
		NMU	Breast	>15	>15	>15	>15
	Primary	RA-6053	Alveolar	>15	26.69	>15	>15
		RA-6034	Kidney	>15	>15	>15	>15
Hamster	Cell Line	HaK	Kidney	2.435	4.881	>15	>15
		HapT1	Pancreas	1.18	0.486	>15	>15
	Primary	HM-6053	Alveolar	>15	>15	>15	>15
		HM-6034	Kidney	>15	>15	>15	>15
Cotton Rat	Cell Line	LCRT	Breast	3.553	2.881	>15	>15
		CCRT	Bone	>15	>15	>15	>15
Rabbit	Cell Line	RK-13	Kidney	9.644	3.624	>15	>15
		SF-1EP	Epidermis	>15	>15	>15	>15
	Primary	N-6053	Alveolar	>15	40.36	>15	>15
		N-6034	Kidney	>15	>15	>15	>15
Dog	Cell Line	DK	Kidney	>15	>15	>15	>15
	Primary	D-6053	Alveolar	>15	>15	>15	>15
		D-6034	Kidney	>15	>15	>15	>15
Cynomolgus monkey	Primary	MK-6053	Alveolar	>15	>15	>15	>15
		MK-6034	Kidney	>15	>15	>15	>15
Pig	Cell Line	LLC-PK1	Kidney	>15	8.422	>15	>15
		PK(15)	Kidney	>15	>15	>15	>15
	Primary	P-6053	Alveolar	>15	13.46	>15	>15
		P-6034	Kidney	2.435	4.881	>15	>15

Animal primary cells and cell lines were infected with either Wt Ad5, ICVB-874, ICVB-1041, or ICVB-1042 followed by cell viability measurement by WST-1 assay 4 or 5 days after infection. Absorbance values relative to uninfected were plotted against MOI (PFU/cell) and plots fitted with a restricted (Bottom = 0.00, Top < 1.20, HillSlope < 0) four parameter non-linear curve to calculate IC_{50} (MOI). IC_{50} values were reported if goodness of fit (R^2) values were greater than or equal to 0.900, otherwise they were reported as >15. ICVB-874 and ICVB-1041 are engineered OVs based on Ad5. Like ICVB-1042, their genomes include the deletion of 12.5K, 6.7K, 19K, RID-alpha, and 14.7K genes in the E3 region while ADP (E3-11.6K) is retained as YPet-P2A-ADP. ICVB-874 retains fiber from Wt Ad5, whereas ICVB-1041 encodes the chimeric Ad5/Ad34 fiber like ICVB-1042. Neither virus incorporates the hexon modification or the dual tumor-selective modifications of E1A and E4orf6/7 unlike ICVB-1042.

Supplementary Table 3. Replication rate of ICVB-1042 in human tumor cell lines.

Tissue	Cell line	ICVB-1042 T80%
Bladder	SW 780	123.86
	HT-1376	95.09
	SCaBER	88.13
	TCCSUP	82.02
	U-BLC1	81.91
	UM-UC-3	78.49
	J82	61.54
	RT4	60.01
	T24	51.81
Brain	LN-18	107.85
	H4	99.2
	M059J	84.25
	M059K	78.68
	LN-229	73.37
	U-138 MG	64.5
	A-172	60.28
	KNS-42	58.97
	SW 1088	58.96
	ANGM-CSS	54.46
	CCF-STTG1	52.86
	T98G	48.62
Breast	HCC70	85.64
	MDA-MB-231	76.53
	HCC1954	74.1
	Hs 578T	57.31
Cervix	Ca Ski	85.59
Colon	LS123	91.39
	SW1116	86.76
	SK-CO-1	81.56
	SNU-C1	75.16
	SW 626	74.51
	T84	68.07
	SW948	59.38
	LoVo	48.72
	HCT-116	47.29
Ovary	SK-OV-3	76.92
	Caov-3	59.01
	ES-2	40.53
Head and neck	UPCI-SCC-154	101.52
	A-253	91.3
	CAL 27	91.03
	FaDu	83.34
	SCC-25	82.88
	SCC-9	82.59
	SCC-15	75.61
	UPCI-SCC-090	75.25
	UPCI-SCC-152	70.55
Lung	A549	100
	NCI-H1975	80.09
Prostate	DU 145	94.45
	PWR-1E	89.09
	LNCaP clone FGC	87.49
	WPE1-NA22	81.25
	MDA PCa 2b	68.91
	NCI-H660	59.64
	VCaP	58.4
	22Rv1	54.22
Skin	MET-4	76.76
	A-375	65.98
	SK-MEL-5	64.54
	RPMI-7951	61.83
	SCC IC1MET	57.76
	A-431	57.5
	SK-MEL-28	53.96
	SK-MEL-24	53.33
	SCC IC18	52.17
	Malme-3M	50.48
	SK-MEL-31	49.12
	SCC T2	46.9
	G-361	46.6
	SK-MEL-2	42.8
Stomach	NCI-N87	87.96
Myeloma	NCI-H929	49.5
B-cell lymphoma	EB-3	0
T-cell lymphoma	CEM/C1	0
	SUP-T1	0

Replication rate of ICVB-1042 was expressed as RelT80 (relative time to achieve 80%-maximum YPet intensity) in fluorescent-based viral kinetics assays at an interpolated MOI of 0.05. For interpolation, linear regression was conducted on a semi-log graph of log-transformed MOI and 80%-YPet timing across examined MOIs. RelT80 value is calculated by dividing the T80 of A549 cells infected with ICVB-1042 by the T80 of a cell line of interest in the same experiment.

Supplementary Table 4. List of human cell lines

Cell line	Tumor type	Vendor	Catalog number
HT-1197	Bladder	ATCC	CRL-1473
HT-1376	Bladder	ATCC	CRL-1472
J82	Bladder	ATCC	HTB-126
RT4	Bladder	ATCC	HTB-26
SCaBER	Bladder	ATCC	HTB-3
SW 780	Bladder	ATCC	CRL-2169
T24	Bladder	ATCC	HTB-4
TCCSUP	Bladder	ATCC	HTB-5
U-BLC1	Bladder	MilliporeSigma	6013102
UM-UC-3	Bladder	ATCC	CRL-1749
SUP-T1	Blood	ATCC	CRL-1942
NCI-H929	Bone	ATCC	CRL-9068
A-172	Brain	ATCC	CRL-1620
ANGM-CSS	Brain	MilliporeSigma	08040401
CCF-STTG1	Brain	ATCC	CRL-1718
H4	Brain	ATCC	HTB-148
KNS-42	Brain	JCRB	IFO50356
LN-18	Brain	ATCC	CRL-2610
LN-229	Brain	ATCC	CRL-2611
M059J	Brain	ATCC	CRL-2366
M059K	Brain	ATCC	CRL-2365
SW 1088	Brain	ATCC	HTB-12
T98G	Brain	ATCC	CRL-1690
U138-MG	Brain	ATCC	HTB-16
HCC1954	Breast	ATCC	CRL-2338
HCC70	Breast	ATCC	CRL-2315
Hs 578T	Breast	ATCC	HTB-126
MDA-MB-231	Breast	ATCC	HTB-26
MDA-MB-415	Breast	ATCC	HTB-128
Ca Ski	Cervical	ATCC	CRL-1550
HCT-116	Colon	ATCC	CCL-247
LoVo	Colon	ATCC	CCL-229

Cell line	Tumor type	Vendor	Catalog number
LS123	Colon	ATCC	CCL-255
SK-CO-1	Colon	ATCC	HTB-39
SW 626	Colon	ATCC	HTB-78
SW948	Colon	ATCC	CCL-237
SW1116	Colon	ATCC	CCL-233
SNU-C1	Colorectal	ATCC	CRL-5972
T84	Colorectal	ATCC	CCL-248
NCI-N87	Gastric	ATCC	CRL-5822
A-253	Head and neck	ATCC	HTB-41
CAL 27	Head and neck	ATCC	CRL-2095
FaDu	Head and neck	ATCC	HTB-43
SCC-9	Head and neck	ATCC	CRL-1629
SCC-15	Head and neck	ATCC	CRL-1623
SCC-25	Head and neck	ATCC	CRL-1628
UPCI-SCC-090	Head and neck	ATCC	CRL-3239
UPCI-SCC-152	Head and neck	ATCC	CRL-3240
UPCI-SCC-154	Head and neck	ATCC	CRL-3241
A549	Lung	ATCC	CCL-185
NCI-H1975	Lung	ATCC	CRL-5908
NCI-H660	Lung	ATCC	CRL-5813
CEM/C1	Lymphoblast	ATCC	CRL-2265
EB-3	Lymphoblast	ATCC	CCL-85
Caov-3	Ovarian	ATCC	HTB-75
SK-OV-3	Ovarian	ATCC	HTB-77
ES-2	Ovarian	ATCC	CRL-1978
22Rv1	Prostate	ATCC	CRL-2505
DU 145	Prostate	ATCC	HTB-81
LNCaP clone FGC	Prostate	ATCC	CRL-1740
MDA PCa 2b	Prostate	ATCC	CRL-2422
PC-3	Prostate	ATCC	CRL-1435
PC-3-Luc2	Prostate	ATCC	CRL-1435-LUC2
PWR-1E	Prostate	ATCC	CRL-11611
VCaP	Prostate	ATCC	CRL-2876
WPE1-NA22	Prostate	ATCC	CRL-2849

Cell line	Tumor type	Vendor	Catalog number
A375	Skin	ATCC	CRL-1619
A375-Luc2	Skin	ATCC	CRL-1619-LUC2
A-431	Skin	ATCC	CRL-2660
G-361	Skin	ATCC	CRL-1424
Malme-3M	Skin	ATCC	HTB-102
MET-4	Skin	Ximbio	153570
RPMI-7951	Skin	ATCC	HTB-66
SCC IC18	Skin	Ximbio	153670
SCC IC1MET	Skin	Ximbio	153676
SCC T2	Skin	Ximbio	15367
SK-MEL-2	Skin	ATCC	HTB-68
SK-MEL-5	Skin	ATCC	HTB-70
SK-MEL-24	Skin	ATCC	HTB-71
SK-MEL-28	Skin	ATCC	HTB-72
SK-MEL-31	Skin	ATCC	HTB-73

Supplementary Table 5. List of human primary normal cells

**Primary cells**	**Vendor**	**Catalogue number**
Aortic smooth muscle cells	Lonza	CC-2571
Astrocytes	Lonza	CC-2565
Bladder smooth muscle cells	Lonza	CC-2533
Bladder epithelial cells	ATCC	PCS-420-010
Bronchial epithelial cells	Lonza	CC-2540
Cardiac fibroblasts	Lonza	CC-2903
Cardiac microvascular endothelial cells	Lonza	CC-7030
Hepatocytes	Lonza	HUCPL
Intestinal epithelial cells	Cell Biologics	H-6061
Keratinocytes	Lonza	00192627
Lung fibroblasts	Lonza	CC-2512
Mammary epithelial cells	Lonza	CC-2551
Melanocytes	Lonza	CC-2586
Ovarian epithelial cells	Cell Biologics	H-6036
Peripheral blood mononuclear cells (PBMC)	STEMCELL Technologies	70500
Prostate epithelial cells	Lonza	CC-2555
Prostate stromal cells	Lonza	CC-2508
Renal cortical epithelial cells	Lonza	CC-2554
Renal epithelial cells	Lonza	CC-2558
Small airway epithelial cells	Lonza	CC-2547
Umbilical vein endothelial cells	Lonza	CC-2519

Supplementary Table 6 Characteristics of dissociated tumor cells

Pt ID	Primary Diagnosis: Diagnosis Name	Tumor Location	Overall Clinical Stage	Pt Age at Collection (yr)	Gender	Race	SKU Name	PTVCs per mL (in millions)	% Cell Viability	HLA-A02 by Flow	EpCAM+	CD45+	CD3+CD4+	CD3+CD8+	CD19+	CD3-/CD56+	CD11b+	CD15+	CD14+	Her2	ER	PR
110006754	Brain Cancer, Glioblastoma Multiforme	-	Grade IV (WHO)	69	F	W	BTC1000-E1110006754080718MS	4.88	65.33	P	0.00	3.06	26.40	12.90	4.29	41.10	3.57	4.76	3.57			
110043044	Brain Cancer, Glioblastoma Multiforme	-	IV	71	F	W	BTC1000-G2110043044012218MS	2.31	61.44	—	0.00	5.39	8.99	2.63	6.12	3.30	66.70	4.24	57.60			
200015596	Breast Cancer, Invasive Ductal Carcinoma	Left breast	II-B	48	F		BTC1000-5F200015596060421MS	0.49	65.29	N	86.10	8.55	46.30	8.58	2.80	0.61	34.60	0.19	31.70	N	P	P
200015498	Breast Cancer, Invasive Ductal Carcinoma						BTC1000-5F200015498061421MS													N	P	P
200000959	Bladder Cancer, Squamous Cell Carcinoma		I	91	M	W	BTC1000-E1200000959100119MS	7.80	83.69	N	89.40	7.33	36.00	9.40	5.06	17.60	38.60	5.77	37.20			
200003341	Bladder Cancer, Transitional Cell Carcinoma (Papillary)	Bladder	I	65	M	W	BTC1000-E1200003341032519MS	4.80	81.77	N	85.30	7.91	51.30	17.20	2.70	10.60	20.00	1.45	20.00			
200020755	Lung Cancer, Adenocarcinoma		II-B	69	M	W	BTC1000-9D200020755092021MS	1.83	59.61	P	21.80	59.30	23.60	35.20	3.36	0.16	27.10	4.56	22.20			
200013795	Lung Cancer, Adenocarcinoma	Right lung	I-B	67	M	W	BTC1000-E1200013795060820MS	1.76	77.81	P	18.70	64.40	20.40	15.00	28.70	9.80	18.50	1.63	16.20			

Abbreviations: ER = estrogen receptor; F = female; ID = identification number; M = male; N = negative; P = Positive; PR = progesterone receptor; Pt = patient; PTVCs = post-thaw viable cells; W = white; yr = years

Note: For all cells, the product description is 1.0 mL Tumor Derived Cells; For all cells, ethnicity was non-Hispanic/Latino except for cells from patient id 200015490 for which the status is not available. For all cells, treatment status was pre-treatment except for cells from patient id 200015498 for which the status is not available.

Supplemental References

1. O'Connor, R. J. & Hearing, P. The E4-6/7 Protein Functionally Compensates for the Loss of E1A Expression in Adenovirus Infection. *J. Virol.* **74**, 5819–5824 (2000).
2. Reichel, R. *et al.* The adenovirus E4 gene, in addition to the E1A gene, is important for trans-activation of E2 transcription and for E2F activation. *J. Virol.* **63**, 3643–3650 (1989).
3. Huang, M. M. & Hearing, P. The adenovirus early region 4 open reading frame 6/7 protein regulates the DNA binding activity of the cellular transcription factor, E2F, through a direct complex. *Genes Dev.* **3**, 1699–1710 (1989).
4. Heise, C. *et al.* An adenovirus E1A mutant that demonstrates potent and selective systemic anti-tumoral efficacy. *Nat. Med.* **6**, 1134–1139 (2000).
5. Stone D, Ni S, Li ZY, et al. Development and assessment of human adenovirus type 11 as a gene transfer vector. *J Virol.* 2005;79(8):5090-104. doi: 10.1128/JVI.79.8.5090-5104.2005
6. Verhaagh S, de Jong E, Goudsmit J et al. Human CD46-transgenic mice in studies involving replication-incompetent adenoviral type 35 vectors. J Gen Virol. 2006;87(Pt 2):255-65. doi: 10.1099/vir.0.81293-0
