# Supplementary material for: Nonclinical characterization of ICVB-1042 as a selective oncolytic adenovirus for solid tumor treatment
Source: Commun Biol. 2024 Sep 13;7:1132. doi: 10.1038/s42003-024-06839-6 (PMC11399272; doi:10.1038/s42003-024-06839-6)
Supplement: Supplementary file 2 — Description of Additional Supplemental Material [file 42003_2024_6839_MOESM2_ESM.pdf]

Description of Additional Supplementary Files

File name: Supplementary Data 1

Description: Source data for Figure 2

File name: Supplementary Data 2

Description: Source data for Figure 3

File name: Supplementary Data 3

Description: Source data for Figure 4

File name: Supplementary Data 4

Description: Source data for Figures 5 and 6

File name: Supplementary Data 5

Description: Source data for Figure 7

File name: Supplementary Data 6

Description: Human plasma membrane and secreted proteins screened by Retrogenix™ Cell Microarray Technology (6497 features)

File name: Supplementary Data 7

Description: Source data for Supplemental Figures 2 B; 5; 6 E, H, J, L, N; 7; 8A; 9; 10; 11; 12; 14
