# Supplementary material for: Nonclinical characterization of ICVB-1042 as a selective oncolytic adenovirus for solid tumor treatment
Source: Commun Biol. 2024 Sep 13;7:1132. doi: 10.1038/s42003-024-06839-6 (PMC11399272; doi:10.1038/s42003-024-06839-6)
Supplement: Supplementary file 8 — Supplementary Data 6 [file 42003_2024_6839_MOESM8_ESM.pdf]

Human plasma membrane and secreted proteins screened by Retrogenix™ Cell Microarray Technology (6497 features)

Individual proteins (6101)

A1BG	A2ML1	AADACL2	AAK1	AAK1	AAMP	ABCA1	ABCA10
ABCA12	ABCA3	ABCA3	ABCA4	ABCA7	ABCA8	ABCA8	ABCB1
ABCB11	ABCB4	ABCB4	ABCB5	ABCB6	ABCC1	ABCC10	ABCC12
ABCC2	ABCC3	ABCC3	ABCC4	ABCC4	ABCC5	ABCC5	ABCC6
ABCC8	ABCC9	ABCE1	ABCG1	ABCG1	ABCG2	ABCG2	ABCG4
ABCG5	ABCG8	ABHD12	ABHD15	ABHD17A	ABHD17B	ABHD18	ABHD2
ABHD6	ABI3BP	ABO	ABRA	ACE	ACE	ACE	ACE
ACE	ACE2	ACE2	ACHE	ACHE	ACHE	ACHE	ACKR1
ACKR1	ACKR1	ACKR2	ACKR3	ACKR3	ACKR4	ACP3	ACP3
ACP3	ACP4	ACP7	ACRBP	ACVR1	ACVR1B	ACVR1B	ACVR1C
ACVR1C	ACVR1C	ACVR2A	ACVR2A	ACVR2B	ACVRL1	ADA	ADA2
ADAM10	ADAM11	ADAM12	ADAM12	ADAM12	ADAM12	ADAM12	ADAM15
ADAM15	ADAM15	ADAM17	ADAM18	ADAM18	ADAM2	ADAM2	ADAM20
ADAM20	ADAM21	ADAM22	ADAM22	ADAM23	ADAM23	ADAM29	ADAM30
ADAM32	ADAM33	ADAM7	ADAM7	ADAM8	ADAM8	ADAM9	ADAM9
ADAMDEC1	ADAMTS1	ADAMTS10	ADAMTS13	ADAMTS15	ADAMTS16	ADAMTS17	ADAMTS18
ADAMTS19	ADAMTS3	ADAMTS4	ADAMTS4	ADAMTS5	ADAMTS6	ADAMTS8	ADAMTS9
ADAMTSL1	ADAMTSL2	ADAMTSL3	ADAMTSL5	ADCY2	ADCY3	ADCY4	ADCY5
ADCY6	ADCY9	ADCYAP1	ADCYAP1R1	ADCYAP1R1	ADCYAP1R1	ADD2	ADGRA1
ADGRA2	ADGRA3	ADGRB1	ADGRB1	ADGRB2	ADGRB3	ADGRD1	ADGRD1
ADGRD2	ADGRE1	ADGRE1	ADGRE2	ADGRE3	ADGRE3	ADGRE5	ADGRE5
ADGRE5	ADGRE5	ADGRF1	ADGRF1	ADGRF1	ADGRF2	ADGRF3	ADGRF3
ADGRF4	ADGRF5	ADGRG1	ADGRG1	ADGRG1	ADGRG1	ADGRG2	ADGRG2
ADGRG2	ADGRG2	ADGRG3	ADGRG4	ADGRG5	ADGRG6	ADGRG7	ADGRL1
ADGRL1	ADGRL4	ADGRL4	ADIPOQ	ADIPOQ	ADIPOR1	ADIPOR2	ADM
ADM2	ADM5	ADORA1	ADORA2A	ADORA2B	ADORA3	ADORA3	ADPGK
ADRA1A	ADRA1A	ADRA1B	ADRA1D	ADRA2A	ADRA2B	ADRA2C	ADRB1
ADRB2	ADRB3	ADTRP	AEBP1	AFM	AFP	AGER	AGER
AGER	AGER	AGER	AGGF1	AGR2	AGRP	AGT	AGTR1
AGTR1	AGTR2	AGTRAP	AGTRAP	AGTRAP	AGTRAP	AHCY	AHSG
AIF1L	AIF1L	AIF1L	AIFM2	AIG1	AIMP1	AJAP1	AKR1A1
AKR1B10	ALB	ALCAM	ALCAM	ALCAM	ALCAM	ALG10	ALG10B
ALK	ALKAL1	ALKAL2	ALOX12	ALPG	ALPI	ALPL	ALPL
ALPP	AMBN	AMBP	AMBP	AMELX	AMELY	AMH	AMHR2
AMIGO1	AMIGO2	AMIGO3	AMN	AMTN	AMTN	AMY1A	AMY2A
AMY2B	ANG	ANGPT1	ANGPT1	ANGPT1	ANGPT2	ANGPT2	ANGPT2
ANGPT2	ANGPT4	ANGPTL2	ANGPTL3	ANGPTL4	ANGPTL5	ANGPTL6	ANGPTL7
ANGPTL8	ANKH	ANKS1B	ANO1	ANO10	ANO10	ANO2	ANO2
ANO3	ANO4	ANO4	ANO5	ANO6	ANO7	ANO9	ANOS1
ANOS1	ANPEP	ANTXR1	ANTXR1	ANTXR1	ANTXR2	ANTXR2	ANTXRL
ANXA1	ANXA1	ANXA13	ANXA13	ANXA2	ANXA2	ANXA2	ANXA2P2

ANXA3	ANXA5	ANXA6	ANXA7	ANXA9	AOAH	AOC1	AOC2
AOC3	AOC3	AP2A2	APBB1	APCDD1	APCS	APELA	APH1B
APH1B	APLN	APLNR	APLP1	APLP1	APLP2	APLP2	APLP2
APMAP	APMAP	APMAP	APOA1	APOA1	APOA2	APOA4	APOA5
APOC1	APOC1	APOC2	APOC3	APOC4	APOD	APOE	APOE
APOF	APOH	APOH	APOL1	APOL1	APOL4	APOL4	APOLD1
APOM	APOM	APOO	APP	APP	APP	AQP1	AQP1
AQP1	AQP1	AQP10	AQP11	AQP2	AQP3	AQP4	AQP5
AQP6	AQP7	AQP7	AQP7P3	AQP8	AQP8	AQP9	ARC
ARHGAP17	ARHGEF1	ARHGEF2	ARL6IP5	ARMH4	ARSF	ARSF	ARSI
ARSJ	ARSK	ART3	ART3	ART4	ART5	ARTN	ASAH1
ASAH2	ASAH2	ASAH2	ASGR1	ASGR1	ASGR2	ASGR2	ASIC1
ASIC1	ASIC2	ASIC2	ASIC3	ASIC3	ASIC4	ASIC5	ASIP
ASPN	ASPRV1	ASTN1	ATP10D	ATP10D	ATP11A	ATP11B	ATP11C
ATP13A1	ATP13A1	ATP13A2	ATP13A4	ATP1A1	ATP1A2	ATP1A3	ATP1A4
ATP1A4	ATP1A4	ATP1B1	ATP1B2	ATP1B3	ATP2B2	ATP2B3	ATP2B4
ATP2C2	ATP4A	ATP4B	ATP5F1A	ATP6AP2	ATP6V0A2	ATP6V0A4	ATP6V0E1
ATP7B	ATP9A	ATP9B	ATRAID	ATRAID	ATRAID	ATRAID	ATRN
ATRN	ATRNL1	ATRNL1	ATRNL1	AVP	AVPR1A	AVPR1B	AVPR2
AVPR2	AXL	AZGP1	AZGP1	B2M	B2M	B3GAT1	B4GALT1
B4GALT1	B4GAT1	BACE1	BACE1	BACE1	BACE1	BACE2	BACE2
BACE2	BAG6	BAGE	BAGE2	BAGE3	BAGE4	BAGE5	BAIAP2L2
BAMBI	BASP1	BCAM	BCAN	BCAN	BCAN	BCHE	BCHE
BDKRB1	BDKRB1	BDKRB2	BDNF	BDNF	BEAN1	BEST1	BEST1
BEST1	BEST2	BEST3	BEST3	BEST4	BGLAP	BGN	BLK
BMP1	BMP10	BMP10	BMP15	BMP2	BMP3	BMP4	BMP5
BMP6	BMP7	BMP8A	BMP8B	BMPER	BMPR1A	BMPR1B	BMPR2
BOC	BOC	BPI	BPI	BPIFA1	BPIFA2	BPIFA3	BPIFB1
BPIFB2	BPIFB3	BPIFB4	BPIFB6	BPIFC	BRICD5	BRICD5	BRINP2
BRINP3	BRS3	BSG	BSG	BSG	BSND	BSPH1	BST1
BST2	BTBD17	BTC	BTC	BTD	BTLA	BTLA	BTLA
BTN1A1	BTN1A1	BTN2A1	BTN2A1	BTN2A1	BTN2A2	BTN2A2	BTN2A2
BTN2A2	BTN2A2	BTN3A1	BTN3A1	BTN3A2	BTN3A2	BTN3A2	BTN3A3
BTN3A3	BTNL2	BTNL3	BTNL8	BTNL8	BTNL9	BTNL9	BVES
C10orf25	C11orf24	C11orf44	C11orf45	C11orf87	C11orf94	C12orf49	C12orf73
C14orf93	C15orf61	C16orf89	C16orf89	C16orf89	C17orf67	C17orf77	C17orf80
C17orf80	C17orf99	C19orf18	C1orf210	C1orf54	C1QA	C1QB	C1QBP
C1QBP	C1QC	C1QL1	C1QL2	C1QL3	C1QL4	C1QTNF1	C1QTNF12
C1QTNF2	C1QTNF3	C1QTNF4	C1QTNF5	C1QTNF6	C1QTNF7	C1QTNF8	C1QTNF9
C1QTNF9B	C1R	C1RL	C1S	C2	C22orf46	C2orf66	C2orf69
C3	C3AR1	C3AR1	C4BPA	C4BPB	C4orf48	C5AR1	C5AR1
C5AR2	C5orf38	C5orf46	C5orf64	C6	C6	C6orf120	C6orf15
C6orf89	C7	C7	C7orf69	C8A	C8A	C8B	C8B
C8G	C8G	C8G	C9	C9	C9orf135	C9orf47	C9orf72
CA11	CA11	CA12	CA12	CA12	CA14	CA2	CA4

CA6	CA9	CABP1	CABP1	CABP2	CABP7	CACHD1	CACNA1A
CACNA1C	CACNA1G	CACNA1S	CACNA2D1	CACNA2D2	CACNA2D2	CACNA2D2	CACNA2D3
CACNA2D4	CACNA2D4	CACNB1	CACNB1	CACNB1	CACNB3	CACNB4	CACNG1
CACNG2	CACNG3	CACNG4	CACNG5	CACNG5	CACNG6	CACNG7	CACNG8
CADM1	CADM1	CADM2	CADM3	CADM3	CADM4	CALCA	CALCB
CALCR	CALCR	CALCRL	CALHM1	CALHM3	CALHM4	CALHM6	CALN1
CALR	CALR	CALU	CALY	CAMK1G	CAMK2A	CAMK2D	CAMK2G
CAMK2N1	CAMLG	CAMP	CANT1	CANT1	CANT1	CAPG	CAPN1
CAPN10	CARTPT	CASP4	CASR	CATSPER1	CATSPER2	CATSPER2	CATSPER2
CATSPER3	CATSPER4	CATSPERD	CATSPERE	CATSPERE	CATSPERG	CATSPERG	CBARP
CBLIF	CBLIF	CBLN1	CBLN1	CBLN2	CBLN3	CBLN3	CBLN4
CBLN4	CCBE1	CCDC126	CCDC134	CCDC3	CCDC70	CCDC70	CCDC80
CCER2	CCK	CCKAR	CCKBR	CCKBR	CCL1	CCL1	CCL11
CCL13	CCL14	CCL14	CCL15	CCL16	CCL17	CCL18	CCL19
CCL2	CCL20	CCL21	CCL22	CCL23	CCL24	CCL25	CCL26
CCL27	CCL28	CCL3	CCL3L1	CCL3L1	CCL4	CCL4L1	CCL5
CCL7	CCL8	CCN1	CCN1	CCN2	CCN2	CCN3	CCN4
CCN5	CCN6	CCNYL1	CCR1	CCR1	CCR10	CCR10	CCR2
CCR3	CCR4	CCR5	CCR6	CCR6	CCR7	CCR7	CCR8
CCR9	CCR9	CCR9	CCRL2	CCRL2	CCRL2	CCSMST1	CD101
CD109	CD14	CD14	CD151	CD160	CD163	CD163	CD163L1
CD164	CD164	CD164L2	CD177	CD177	CD180	CD19	CD19
CD1A	CD1A	CD1B	CD1C	CD1D	CD1E	CD2	CD200
CD200	CD200	CD200R1	CD200R1	CD200R1	CD200R1	CD200R1L	CD207
CD209	CD209	CD209	CD22	CD22	CD22	CD226	CD24
CD244	CD244	CD247	CD247	CD248	CD27	CD274	CD274
CD276	CD276	CD28	CD300A	CD300C	CD300E	CD300H	CD300H
CD300LB	CD300LB	CD300LD	CD300LF	CD300LF	CD300LG	CD302	CD302
CD320	CD320	CD33	CD33	CD34	CD36	CD37	CD38
CD3D	CD3E	CD3G	CD4	CD4	CD40	CD40	CD40
CD40	CD40LG	CD40LG	CD44	CD44	CD44	CD44	CD46
CD46	CD46	CD47	CD47	CD47	CD48	CD5	CD52
CD53	CD55	CD55	CD58	CD58	CD59	CD59	CD5L
CD5L	CD6	CD6	CD6	CD63	CD63	CD68	CD69
CD7	CD7	CD70	CD70	CD72	CD74	CD74	CD74
CD79A	CD79B	CD80	CD81	CD82	CD83	CD84	CD84
CD86	CD86	CD8A	CD8A	CD8A	CD8B	CD8B	CD8B
CD8B	CD8B	CD8B	CD8B2	CD8B2	CD9	CD93	CD96
CD96	CD96	CD99	CD99L2	CD99L2	CD99L2	CD99L2	CDCP1
CDCP1	CDCP1	CDCP2	CDH1	CDH10	CDH11	CDH12	CDH13
CDH15	CDH16	CDH16	CDH17	CDH18	CDH18	CDH19	CDH2
CDH2	CDH20	CDH22	CDH23	CDH24	CDH26	CDH3	CDH4
CDH5	CDH6	CDH6	CDH7	CDH7	CDH8	CDH9	CDHR1
CDHR1	CDHR1	CDHR2	CDHR3	CDHR4	CDHR4	CDHR5	CDIPT
CDNF	CDON	CDSN	CDSN	CEACAM1	CEACAM1	CEACAM1	CEACAM1

CEACAM16	CEACAM19	CEACAM19	CEACAM20	CEACAM20	CEACAM21	CEACAM21	CEACAM3
CEACAM4	CEACAM5	CEACAM6	CEACAM7	CEACAM8	CEL	CELA1	CELA2A
CELA2B	CELSR1	CELSR2	CELSR3	CELSR3	CEMIP2	CEND1	CER1
CES4A	CES5A	CETP	CFB	CFB	CFC1	CFC1	CFC1B
CFD	CFH	CFHR1	CFHR2	CFHR3	CFHR4	CFHR5	CFI
CFP	CGA	CGB1	CGB2	CGB3	CGB7	CGREF1	CHAD
CHADL	CHGA	CHGB	CHI3L1	CHI3L2	CHIA	CHIC2	CHID1
CHIT1	CHL1	CHL1	CHODL	CHODL	CHRD	CHRDL1	CHRDL2
CHRDL2	CHRFAM7A	CHRFAM7A	CHRM1	CHRM1	CHRM1	CHRM2	CHRM3
CHRM3	CHRM4	CHRM5	CHRNA1	CHRNA1	CHRNA10	CHRNA2	CHRNA3
CHRNA3	CHRNA3	CHRNA4	CHRNA4	CHRNA4	CHRNA5	CHRNA6	CHRNA7
CHRNA7	CHRNA9	CHRNB1	CHRNB2	CHRNB3	CHRNB4	CHRND	CHRNE
CHRNG	CHRNG	CHST9	CHSY1	CIB1	CILP	CILP2	CKAP4
CKLF	CKLF	CLC	CLC	CLCA1	CLCA1	CLCA2	CLCA2
CLCA4	CLCA4	CLCF1	CLCN1	CLCN2	CLCN2	CLCN3	CLCN4
CLCN5	CLCNKB	CLCNKB	CLDN1	CLDN10	CLDN11	CLDN12	CLDN14
CLDN14	CLDN15	CLDN16	CLDN17	CLDN18	CLDN18	CLDN19	CLDN19
CLDN2	CLDN20	CLDN22	CLDN23	CLDN24	CLDN25	CLDN3	CLDN34
CLDN4	CLDN5	CLDN6	CLDN7	CLDN8	CLDN9	CLDND1	CLDND1
CLEC10A	CLEC10A	CLEC11A	CLEC12A	CLEC12B	CLEC12B	CLEC14A	CLEC17A
CLEC18A	CLEC18B	CLEC18C	CLEC19A	CLEC1A	CLEC1B	CLEC1B	CLEC2A
CLEC2B	CLEC2D	CLEC2D	CLEC2D	CLEC3A	CLEC3B	CLEC4A	CLEC4C
CLEC4D	CLEC4E	CLEC4F	CLEC4F	CLEC4G	CLEC4G	CLEC4M	CLEC4M
CLEC5A	CLEC6A	CLEC7A	CLEC7A	CLEC7A	CLEC7A	CLEC9A	CLECL1
CLIC1	CLIC4	CLIC5	CLIC6	CLMP	CLMP	CLN3	CLPS
CLPS	CLPSL1	CLPSL2	CLPTM1	CLPTM1	CLPTM1L	CLRN1	CLRN2
CLSTN1	CLSTN1	CLSTN2	CLSTN3	CLTRN	CLU	CLUL1	CMA1
CMKLR1	CMKLR1	CMTM2	CMTM3	CMTM4	CMTM5	CMTM5	CMTM6
CMTM7	CNDP1	CNGA1	CNGA2	CNGA3	CNGA3	CNGA4	CNGB1
CNGB3	CNIH2	CNIH3	CNMD	CNMD	CNNM1	CNNM2	CNNM3
CNNM4	CNP	CNPY4	CNR1	CNR1	CNR2	CNST	CNST
CNTFR	CNTN1	CNTN2	CNTN3	CNTN4	CNTN4	CNTN5	CNTN6
CNTNAP1	CNTNAP2	CNTNAP3	CNTNAP3B	CNTNAP4	CNTNAP5	COCH	COL10A1
COL13A1	COL15A1	COL17A1	COL19A1	COL1A1	COL1A1	COL1A2	COL1A2
COL20A1	COL21A1	COL23A1	COL23A1	COL25A1	COL25A1	COL25A1	COL25A1
COL26A1	COL28A1	COL3A1	COL4A3	COL6A1	COL6A1	COL6A2	COL6A2
COL6A2	COL6A2	COL8A1	COL9A1	COL9A2	COLEC10	COLEC11	COLEC12
COLQ	COMP	COMT	COMT	CORIN	CORT	CP	CPA1
CPA2	CPA4	CPA5	CPA6	CPB1	CPB2	CPD	CPE
CPE	CPLX3	CPM	CPN1	CPN1	CPN2	CPO	CPQ
CPXM1	CPXM2	CPZ	CR1	CR1L	CR2	CR2	CRB1
CRB2	CRB3	CREG1	CREG2	CRELD1	CRELD1	CRELD2	CRH
CRHBP	CRHBP	CRHR1	CRHR1	CRHR2	CRIM1	CRIM1	CRISP2
CRISP3	CRISPLD1	CRISPLD2	CRLF1	CRLF2	CRLF2	CRLF3	CRP
CRTAC1	CRTAC1	CRTAM	CRTAP	CRTAP	CSF1	CSF1	CSF1

CSF1R	CSF2	CSF2RA	CSF2RA	CSF2RA	CSF2RA	CSF2RA	CSF2RB
CSF2RB	CSF3	CSF3R	CSF3R	CSF3R	CSH1	CSH2	CSHL1
CSMD2	CSN1S1	CSN2	CSN3	CSNK2B	CSPG4	CSPG5	CSPG5
CSPG5	CST1	CST11	CST2	CST3	CST4	CST5	CST6
CST7	CST8	CST9	CST9L	CSTL1	CT83	CTF1	CTHRC1
CTLA4	CTNNA1	CTNNB1	CTNS	CTRB1	CTRB2	CTSB	CTSB
CTSD	CTSG	CTSK	CTSS	CUTA	CUZD1	CX3CL1	CX3CL1
CX3CL1	CX3CR1	CX3CR1	CXADR	CXADR	CXCL1	CXCL10	CXCL11
CXCL12	CXCL13	CXCL14	CXCL16	CXCL16	CXCL16	CXCL17	CXCL2
CXCL3	CXCL5	CXCL6	CXCL8	CXCL9	CXCR1	CXCR1	CXCR2
CXCR2	CXCR3	CXCR3	CXCR4	CXCR4	CXCR5	CXCR5	CXCR6
CXCR6	CXorf66	CYB5D2	CYBA	CYBB	CYBRD1	CYSLTR1	CYSLTR1
CYSLTR2	CYTH1	CYTL1	CYYR1	DAG1	DAG1	DAGLA	DAGLB
DAND5	DAPP1	DBH	DCBLD1	DCBLD2	DCC	DCD	DCHS1
DCLK1	DCN	DCST1	DCST2	DCSTAMP	DDR1	DDR1	DDR2
DEAF1	DEFA1	DEFA3	DEFA3	DEFA4	DEFA5	DEFA6	DEFB1
DEFB103A; DEF	DEFB104A; DEF	DEFB105A; DEF	DEFB106A	DEFB106B	DEFB107A	DEFB107B	DEFB108B
DEFB108B	DEFB110	DEFB110	DEFB112	DEFB112	DEFB113	DEFB113	DEFB114
DEFB115	DEFB115	DEFB116	DEFB116	DEFB118	DEFB119	DEFB121	DEFB123
DEFB124	DEFB124	DEFB125	DEFB126	DEFB127	DEFB128	DEFB129	DEFB130A
DEFB131A	DEFB131B	DEFB132	DEFB133	DEFB134	DEFB135	DEFB135	DEFB136
DEFB136	DEFB4A	DES	DGCR2	DGKB	DHH	DHH	DHRS11
DHRS13	DHRS4L2	DHRS7B	DHRS7C	DHRSX	DIO3	DIO3	DIPK2A
DIPK2B	DIRAS1	DIRAS2	DIRAS3	DISP1	DKK1	DKK1	DKK2
DKK3	DKK4	DKKL1	DLG2	DLG3	DLG4	DLK1	DLK2
DLK2	DLL1	DLL3	DLL3	DLL4	DMD	DMKN	DMP1
DNAJB4	DNAJC16	DNASE1	DNASE1L2	DNASE1L3	DNER	DPEP1	DPEP2
DPEP2	DPP10	DPP4	DPP4	DPP6	DPP7	DPT	DRAM2
DRAXIN	DRD1	DRD2	DRD2	DRD3	DRD4	DRD5	DSC1
DSC2	DSC3	DSCAM	DSCAML1	DSG1	DSG2	DSG3	DSG4
DUOX1	DUOX2	DUOXA1	DUOXA1	DUOXA2	DUSP15	DYNAP	DYSF
EBAG9	EBI3	EBI3	ECE1	ECM1	ECM2	ECRG4	ECSCR
EDA	EDA	EDA	EDA	EDA	EDA2R	EDAR	EDDM13
EDDM3A	EDDM3B	EDIL3	EDN1	EDN2	EDN3	EDNRA	EDNRB
EFEMP1	EFEMP2	EFNA1	EFNA2	EFNA3	EFNA4	EFNA5	EFNB1
EFNB2	EFNB3	EFR3A	EGF	EGFL6	EGFL7	EGFL8	EGFLAM
EGFLAM	EGFR	EGFR	EGFR	EGFR	EGFR	ELAPOR1	ELAPOR1
ELAPOR1	ELAPOR2	ELFN1	ELN	ELSPBP1	EMB	EMC1	EMC10
EMC10	EMC7	EMCN	EMCN	EMID1	EMILIN1	EMILIN2	EMILIN3
EMP1	EMP2	EMP2	ENAM	ENDOD1	ENDOU	ENG	ENHO
ENO1	ENO2	ENOX1	ENOX1	ENOX2	ENOX2	ENPEP	ENPP1
ENPP1	ENPP1	ENPP2	ENPP2	ENPP3	ENPP3	ENPP4	ENPP5
ENPP5	ENPP6	ENPP7	ENTPD1	ENTPD1	ENTPD2	ENTPD3	ENTPD3
ENTPD5	ENTPD6	ENTPD8	ENTPD8	EPB41L2	EPB41L3	EPB41L3	EPB41L5
EPCAM	EPDR1	EPDR1	EPGN	EPGN	EPGN	EPHA1	EPHA10

EPHA10	EPHA10	EPHA2	EPHA3	EPHA3	EPHA3	EPHA4	EPHA4
EPHA5	EPHA6	EPHA7	EPHA7	EPHA8	EPHA8	EPHB1	EPHB2
EPHB2	EPHB3	EPHB4	EPHB6	EPHX4	EPO	EPOR	EPPIN
EPYC	ERAS	ERBB2	ERBB3	ERBB3	ERBB3	ERBB3	ERBB4
ERBB4	EREG	ERFE	ERMAP	ERVFRD-1	ERVH48-1	ERVMER34-1	ERVMER34-1
ERVV-1	ERVV-2	ERVW-1	ESAM	ESM1	ESR1	ESYT3	EVA1C
EVA1C	EVC	EVC2	EVI2A	EVI2A	EVI2A	EVI2B	EXTL2
EXTL2	F10	F10	F10	F11	F11	F11R	F12
F12	F13A1	F13B	F2	F2	F2R	F2R	F2RL1
F2RL1	F2RL2	F2RL3	F3	F3	F5	F5	F5
F5	F7	F7	F8	F8	F9	F9	FABP5
FADS2	FAIM2	FAM168B	FAM168B	FAM171A1	FAM171A2	FAM171B	FAM171B
FAM172A	FAM174A	FAM174B	FAM180A	FAM180B	FAM187B	FAM187B	FAM200A
FAM209A	FAM209B	FAM20A	FAM20C	FAM234A	FAM24A	FAM24B	FAM3A
FAM3B	FAM3D	FAM3D	FAP	FAP	FAS	FAS	FAS
FAS	FAS	FAS	FAS	FASLG	FASLG	FBLN1	FBLN2
FBLN5	FBLN7	FBN3	FCAMR	FCAR	FCAR	FCAR	FCAR
FCAR	FCAR	FCAR	FCAR	FCAR	FCER1A	FCER1G	FCER1G
FCER2	FCER2	FCGR1A	FCGR1B	FCGR1B	FCGR2A	FCGR2A	FCGR2B
FCGR2B	FCGR2C	FCGR3A	FCGR3A	FCGR3A	FCGR3B	FCGR3B	FCGRT
FCMR	FCMR	FCMR	FCN1	FCN1	FCN2	FCN3	FCRL1
FCRL2	FCRL2	FCRL2	FCRL3	FCRL3	FCRL4	FCRL5	FCRL6
FCRLA	FDCSP	FETUB	FFAR1	FFAR2	FFAR3	FFAR4	FGA
FGA	FGB	FGB	FGF1	FGF10	FGF10	FGF16	FGF17
FGF18	FGF18	FGF19	FGF2	FGF20	FGF21	FGF22	FGF23
FGF3	FGF4	FGF5	FGF6	FGF6	FGF7	FGF8	FGF9
FGFBP1	FGFBP1	FGFBP2	FGFBP3	FGFR1	FGFR1	FGFR1	FGFR1
FGFR1	FGFR2	FGFR2	FGFR3	FGFR3	FGFR3	FGFR4	FGFR4
FGFRL1	FGG	FGG	FGL1	FGL2	FIBCD1	FIBIN	FITM1
FITM2	FJX1	FKRP	FLNB	FLOT1	FLRT1	FLRT1	FLRT1
FLRT1	FLRT2	FLRT2	FLRT3	FLRT3	FLRT3	FLT1	FLT1
FLT1	FLT3	FLT3LG	FLT4	FLVCR1	FLVCR2	FMO5	FMOD
FMR1NB	FN1	FNDC10	FNDC4	FNDC4	FNDC5	FNDC5	FNDC5
FNDC5	FNDC5	FNDC7	FOLH1	FOLH1	FOLH1	FOLR1	FOLR1
FOLR2	FOLR2	FOLR3	FPR1	FPR2	FPR2	FPR3	FRRS1L
FRZB	FRZB	FSHB	FSHR	FST	FSTL1	FSTL3	FSTL4
FSTL5	FUCA2	FURIN	FURIN	FUT6	FXYD1	FXYD2	FXYD3
FXYD4	FXYD5	FXYD6	FXYD6	FXYD7	FZD1	FZD10	FZD2
FZD3	FZD4	FZD5	FZD6	FZD7	FZD8	FZD9	GABBR1
GABBR1	GABBR1	GABBR1	GABBR2	GABBR2	GABRA1	GABRA2	GABRA3
GABRA4	GABRA5	GABRA5	GABRA6	GABRB1	GABRB2	GABRB2	GABRB3
GABRB3	GABRD	GABRE	GABRE	GABRG1	GABRG2	GABRG3	GABRP
GABRQ	GABRR1	GABRR1	GABRR1	GABRR2	GABRR3	GAD2	GAL
GALNT1	GALNT2	GALNT2	GALP	GALP	GALR1	GALR2	GALR3
GAPT	GARS1	GAS1	GAS6	GASK1A	GAST	GAST	GATD1

GBP1	GBP1	GBP1	GC	GC	GCG	GCG	GCGR
GCNT7	GDE1	GDF1	GDF10	GDF11	GDF15	GDF2	GDF2
GDF3	GDF5	GDF6	GDF7	GDF9	GDNF	GDPD1	GDPD1
GDPD2	GDPD3	GDPD4	GDPD5	GDPD5	GFER	GFOD1	GFOD2
GFRA1	GFRA1	GFRA2	GFRA3	GFRA4	GFRA4	GFRA4	GFRAL
GGH	GGH	GGT1	GGT1	GGT2	GGT5	GGT5	GGT5
GGT6	GGT7	GGTLC1	GGTLC2	GH1	GH2	GHR	GHRH
GHRHR	GHRL	GHSR	GHSR	GHSR	GINM1	GIP	GIPR
GJA1	GJA10	GJA3	GJA3	GJA4	GJA5	GJA8	GJA9
GJB1	GJB2	GJB3	GJB4	GJB5	GJB6	GJB7	GJC1
GJC2	GJC3	GJD2	GJD3	GJD3	GJD4	GKN1	GKN2
GKN2	GLB1L	GLB1L2	GLDN	GLG1	GLIPR1	GLIPR1L1	GLIPR1L2
GLIPR1L2	GLP1R	GLP2R	GLRA1	GLRA1	GLRA2	GLRA2	GLRA3
GLRA4	GLRB	GLT1D1	GML	GNA11	GNA13	GNA15	GNAS
GNAS	GNAS	GNB1	GNB3	GNB3	GNB4	GNG11	GNG12
GNG2	GNG5	GNGT1	GNLY	GNPTG	GNRH1	GNRH2	GNRHR
GP1BA	GP1BA	GP1BA	GP1BB	GP2	GP2	GP5	GP6
GP9	GPA33	GPBAR1	GPC1	GPC1	GPC2	GPC2	GPC2
GPC3	GPC3	GPC3	GPC4	GPC4	GPC5	GPC5	GPC6
GPC6	GPER1	GPER1	GPHA2	GPHB5	GPI	GPIHBP1	GPLD1
GPLD1	GPM6A	GPM6B	GPM6B	GPNMB	GPNMB	GPR1	GPR101
GPR107	GPR108	GPR119	GPR12	GPR132	GPR132	GPR135	GPR135
GPR137	GPR137B	GPR137C	GPR139	GPR141	GPR142	GPR143	GPR143
GPR146	GPR148	GPR149	GPR15	GPR150	GPR151	GPR151	GPR152
GPR153	GPR156	GPR157	GPR157	GPR158	GPR15L	GPR160	GPR161
GPR161	GPR162	GPR162	GPR17	GPR17	GPR171	GPR173	GPR173
GPR174	GPR176	GPR179	GPR18	GPR182	GPR182	GPR183	GPR183
GPR20	GPR21	GPR22	GPR25	GPR26	GPR27	GPR3	GPR3
GPR31	GPR32	GPR33	GPR34	GPR34	GPR35	GPR37	GPR37L1
GPR39	GPR4	GPR42	GPR45	GPR50	GPR52	GPR55	GPR55
GPR6	GPR61	GPR62	GPR63	GPR65	GPR65	GPR68	GPR75
GPR78	GPR82	GPR83	GPR84	GPR84	GPR85	GPR85	GPR87
GPR88	GPR89B	GPRC5A	GPRC5B	GPRC5B	GPRC5C	GPRC5C	GPRC5C
GPRC5C	GPRC5D	GPRC6A	GPX3	GPX5	GPX6	GPX7	GRAMD1A
GRAMD1B	GRAMD1C	GRAMD2A	GREM1	GREM2	GRIA1	GRIA2	GRIA3
GRIA3	GRIA4	GRIA4	GRID1	GRID1	GRID2	GRIK1	GRIK1
GRIK2	GRIK2	GRIK3	GRIK4	GRIK5	GRIN1	GRIN1	GRIN2A
GRIN2B	GRIN2C	GRIN2D	GRIN3A	GRIN3B	GRK7	GRM1	GRM2
GRM3	GRM3	GRM4	GRM5	GRM6	GRM7	GRM8	GRN
GRP	GRPR	GSG1L	GSG1L	GSG1L	GSG1L2	GSN	GUCA2A
GUCA2B	GUCY2C	GUCY2C	GUCY2D	GUCY2F	GYPA	GYPB	GYPC
GYPC	GYPE	GZMA	GZMA	GZMK	GZMM	HABP2	HAMP
HAPLN1	HAPLN2	HAPLN3	HAPLN3	HAPLN4	HAS1	HAS2	HAS3
HAS3	HAS3	HAVCR1	HAVCR1	HAVCR2	HBEGF	HBEGF	HCAR1
HCAR1	HCAR2	HCAR3	HCG22	HCK	HCN1	HCN2	HCN3

HCN4	HCRTR1	HCRTR2	HCST	HDGF	HEG1	HEPACAM	HEPH
HEPHL1	HFE	HFE	HFE	HGFAC	HHIP	HHIP	HHIPL1
HHIPL2	HHLA1	HHLA2	HIDE1	HIGD1B	HIGD1C	HIGD2B	HILPDA
HILPDA	HJV	HJV	HJV	HLA-A	HLA-A	HLA-A	HLA-B
HLA-C	HLA-DOA	HLA-DOB	HLA-DPA1	HLA-DPA1	HLA-DPB1	HLA-DPB1	HLA-DQA1
HLA-DQA2	HLA-DQB1	HLA-DQB2	HLA-DQB2	HLA-DRA	HLA-DRB1	HLA-DRB1	HLA-DRB3
HLA-DRB4	HLA-DRB5	HLA-E	HLA-F	HLA-G	HLA-G	HLA-H	HM13
HMGB1	HMGB2	HMMR	HMMR	HMSD	HNRNPA2B1	HOMER1	HOMER2
HOMER3	HP	HPN	HPR	HPSE	HPSE2	HPX	HRG
HRH1	HRH2	HRH2	HRH3	HRH4	HS6ST1	HSD11B1L	HSD17B11
HSD17B13	HSD17B7	HSP90AB1	HSPB6	HTN1	HTN3	HTR1A	HTR1B
HTR1D	HTR1D	HTR1E	HTR1F	HTR2A	HTR2A	HTR2A	HTR2B
HTR2C	HTR3A	HTR3A	HTR3B	HTR3C	HTR3C	HTR3D	HTR3D
HTR3E	HTR3E	HTR4	HTR5A	HTR6	HTR7	HTR7	HTRA1
HTRA3	HTRA4	HVCN1	HYAL1	HYAL2	HYAL3	HYAL4	IAPP
IBSP	ICAM1	ICAM2	ICAM2	ICAM3	ICAM4	ICAM4	ICAM4
ICAM4	ICAM5	ICOS	ICOS	ICOSLG	IDE	IDE	IER3
IFI30	IFI30	IFITM1	IFITM10	IFITM2	IFITM3	IFITM5	IFNA1
IFNA10	IFNA14	IFNA16	IFNA16	IFNA17	IFNA2	IFNA21	IFNA4
IFNA5	IFNA6	IFNA7	IFNA8	IFNAR1	IFNAR2	IFNAR2	IFNAR2
IFNB1	IFNE	IFNG	IFNG	IFNGR1	IFNGR2	IFNGR2	IFNK
IFNL1	IFNL2	IFNL3	IFNL4	IFNLR1	IFNLR1	IFNW1	IGDCC3
IGDCC4	IGF1	IGF1R	IGF2	IGFALS	IGFBP1	IGFBP2	IGFBP3
IGFBP4	IGFBP5	IGFBP6	IGFBP7	IGFBPL1	IGFL1	IGFL2	IGFL3
IGFL4	IGFLR1	IGHA1	IGHA1	IGHA2	IGHD	IGHE	IGHG1
IGHG2	IGHG3	IGHG3	IGHG4	IGHM	IGHM	IGIP	IGKC
IGLC1	IGLC2	IGLC3	IGLC6	IGLC7	IGLL1	IGLL1	IGLL5
IGLL5	IGLON5	IGSF1	IGSF1	IGSF1	IGSF11	IGSF11	IGSF21
IGSF23	IGSF3	IGSF5	IGSF6	IGSF8	IGSF9	IGSF9	IGSF9B
IGSF9B	IHH	IHH	IL10	IL10RA	IL10RB	IL11	IL11RA
IL11RA	IL11RA	IL12A	IL12B	IL12B	IL12B	IL12RB1	IL12RB1
IL12RB2	IL13	IL13RA1	IL13RA2	IL15	IL15	IL15RA	IL15RA
IL15RA	IL16	IL16	IL17A	IL17B	IL17C	IL17D	IL17F
IL17RA	IL17RA	IL17RB	IL17RC	IL17RC	IL17RD	IL17RE	IL17RE
IL18	IL18	IL18BP	IL18R1	IL18RAP	IL19	IL1A	IL1A
IL1B	IL1B	IL1F10	IL1R1	IL1R1	IL1R1	IL1R2	IL1R2
IL1R2	IL1RAP	IL1RAP	IL1RAP	IL1RAPL1	IL1RAPL2	IL1RL1	IL1RL1
IL1RL1	IL1RL2	IL1RL2	IL1RN	IL1RN	IL1RN	IL2	IL20
IL20RA	IL20RB	IL21	IL21R	IL22	IL22RA1	IL22RA2	IL23A
IL23A	IL23R	IL24	IL25	IL26	IL26	IL27	IL27RA
IL2RA	IL2RB	IL2RG	IL3	IL31	IL31RA	IL31RA	IL31RA
IL32	IL33	IL34	IL36A	IL36B	IL36G	IL36RN	IL37
IL3RA	IL3RA	IL4	IL4R	IL4R	IL5	IL5RA	IL5RA
IL6	IL6R	IL6R	IL6R	IL6ST	IL6ST	IL6ST	IL7
IL7R	IL9	IL9R	IL9R	ILDR1	ILDR1	IMPG1	INHA

INHBA	INHBB	INHBC	INHBE	INPP5D	INS	INSL3	INSL4
INSL5	INSL6	INSR	INSR	INSRR	ISG15	ISLR	ISLR
ISLR2	ISM1	ISM2	ITFG1	ITFG1	ITGA10	ITGA11	ITGA2
ITGA2B	ITGA3	ITGA4	ITGA5	ITGA6	ITGA7	ITGA7	ITGA8
ITGA9	ITGA9	ITGAD	ITGAE	ITGAL	ITGAM	ITGAM	ITGAM
ITGAV	ITGAV	ITGAX	ITGB1	ITGB1	ITGB1	ITGB1	ITGB2
ITGB3	ITGB4	ITGB5	ITGB6	ITGB7	ITGB8	ITGB8	ITGBL1
ITGBL1	ITIH1	ITIH2	ITIH3	ITIH4	ITIH6	ITLN1	ITLN1
ITLN1	ITLN2	ITM2A	ITM2B	ITM2B	ITM2C	ITPRIP	ITPRIPL1
ITPRIPL2	IYD	IZUMO1	IZUMO1R	IZUMO2	IZUMO2	IZUMO3	IZUMO4
JAG1	JAG2	JAM2	JAM3	JAM3	JAML	JAML	JCHAIN
JTB	KARS1	KAZALD1	KCNA1	KCNA10	KCNA2	KCNA2	KCNA3
KCNA4	KCNA5	KCNA5	KCNA6	KCNA7	KCNAB2	KCNB1	KCNB2
KCNC1	KCNC2	KCNC3	KCNC4	KCND1	KCND2	KCND3	KCNE1
KCNE2	KCNE3	KCNE4	KCNE4	KCNE5	KCNG1	KCNG1	KCNG3
KCNG4	KCNG4	KCNH1	KCNH2	KCNH2	KCNH3	KCNH4	KCNH5
KCNH6	KCNH6	KCNH7	KCNH7	KCNH8	KCNIP1	KCNIP1	KCNIP2
KCNIP2	KCNIP3	KCNJ1	KCNJ1	KCNJ10	KCNJ11	KCNJ11	KCNJ12
KCNJ13	KCNJ14	KCNJ15	KCNJ16	KCNJ18	KCNJ2	KCNJ3	KCNJ4
KCNJ5	KCNJ6	KCNJ8	KCNJ9	KCNK1	KCNK10	KCNK12	KCNK13
KCNK16	KCNK17	KCNK18	KCNK2	KCNK2	KCNK3	KCNK4	KCNK5
KCNK6	KCNK7	KCNK7	KCNK7	KCNK9	KCNMA1	KCNMA1	KCNMB1
KCNMB1	KCNMB2	KCNMB3	KCNMB4	KCNN1	KCNN2	KCNN2	KCNN3
KCNN3	KCNN3	KCNN4	KCNQ1	KCNQ1	KCNQ2	KCNQ2	KCNQ3
KCNQ4	KCNQ5	KCNQ5	KCNS1	KCNS2	KCNS3	KCNT1	KCNT2
KCNV1	KCNV2	KCT2	KCTD12	KDR	KEL	KERA	KIAA0319
KIAA0319	KIAA0319L	KIAA0319L	KIAA2013	KIDINS220	KIR2DL1	KIR2DL1	KIR2DL2
KIR2DL3	KIR2DL4	KIR2DL5A	KIR2DL5B	KIR2DS1	KIR2DS2	KIR2DS2	KIR2DS3
KIR2DS4	KIR2DS5	KIR3DL1	KIR3DL2	KIR3DL3	KIR3DS1	KIR3DS1	KIR3DX1
KIRREL1	KIRREL2	KIRREL2	KIRREL3	KIRREL3	KISS1	KISS1R	KIT
KIT	KITLG	KITLG	KITLG	KL	KL	KLB	KLK10
KLK11	KLK12	KLK13	KLK14	KLK15	KLK2	KLK3	KLK4
KLK5	KLK6	KLK7	KLK7	KLK8	KLK9	KLKB1	KLKB1
KLRB1	KLRC1	KLRC1	KLRC2	KLRC2	KLRC3	KLRC4	KLRD1
KLRF1	KLRF1	KLRF2	KLRG1	KLRG2	KLRK1	KNG1	KNG1
KRAS	KREMEN1	KREMEN2	KREMEN2	KRT10	KRT19	KRTDAP	L1CAM
LACRT	LAD1	LAG3	LAG3	LAIR1	LAIR2	LAIR2	LALBA
LALBA	LAMA4	LAMA4	LAMB2	LAMB3	LAMC1	LAMC3	LAMP1
LAMP2	LAMP2	LAMP5	LAPTM4B	LAS2	LAS2	LAT	LAT
LAT	LAT2	LAX1	LAX1	LAYN	LAYN	LBP	LBP
LCAT	LCAT	LCK	LCK	LCN1	LCN10	LCN12	LCN15
LCN2	LCN6	LCN8	LCN9	LCN9	LCP1	LCT	LCTL
LDLR	LDLR	LDLRAD2	LDLRAD3	LEAP2	LECT2	LEFTY1	LEFTY2
LEG1	LEG1	LEP	LEPR	LEPR	LEPR	LGALS1	LGALS1
LGALS1	LGALS3	LGALS3	LGALS3BP	LGALS7	LGALS9	LGALS9	LGALS9

LGI1	LGI2	LGI3	LGI4	LGR4	LGR4	LGR5	LGR6
LGR6	LHB	LHCGR	LHFPL2	LHFPL3	LHFPL3	LHFPL4	LHFPL5
LIF	LIFR	LILRA1	LILRA2	LILRA2	LILRA3	LILRA3	LILRA4
LILRA5	LILRA5	LILRA6	LILRB1	LILRB1	LILRB2	LILRB3	LILRB4
LILRB5	LIM2	LIM2	LIME1	LIMS2	LINGO1	LINGO1	LINGO2
LINGO3	LINGO4	LIPC	LIPC	LIPF	LIPG	LIPH	LIPH
LIPI	LIPI	LIPK	LIPM	LIPN	LLCFC1	LMBR1	LMBR1L
LMBR1L	LMBRD1	LMBRD1	LMBRD2	LNPEP	LNPEP	LNPEP	LOX
LOXL1	LOXL2	LOXL3	LOXL4	LPAR1	LPAR1	LPAR2	LPAR3
LPAR4	LPAR5	LPAR5	LPAR6	LPAR6	LPCAT1	LPCAT2	LPL
LPL	LPO	LRCH3	LRCH3	LRCH3	LRFN1	LRFN2	LRFN3
LRFN4	LRFN5	LRG1	LRIG1	LRIG1	LRIG2	LRIG3	LRIT2
LRP1	LRP10	LRP11	LRP12	LRP3	LRP4	LRP5	LRP6
LRP8	LRP8	LRP8	LRP8	LRP8	LRP8	LRRC15	LRRC17
LRRC19	LRRC25	LRRC26	LRRC32	LRRC37B	LRRC38	LRRC3C	LRRC4
LRRC4	LRRC4B	LRRC4C	LRRC52	LRRC55	LRRC8A	LRRC8B	LRRC8B
LRRC8C	LRRC8D	LRRC8E	LRRN1	LRRN2	LRRN3	LRRN4	LRRN4CL
LRRTM1	LRRTM2	LRRTM3	LRRTM3	LRRTM4	LRRTM4	LRTM1	LRTM2
LSAMP	LSP1	LSR	LST1	LST1	LTA	LTA	LTA
LTB	LTB4R	LTB4R	LTB4R2	LTB4R2	LTBP2	LTBR	LTF
LTF	LTK	LUM	LUZP2	LY6D	LY6E	LY6G5B	LY6G5B
LY6G5C	LY6G5C	LY6G6C	LY6G6D	LY6G6F	LY6H	LY6K	LY6K
LY6L	LY86	LY86	LY9	LY96	LY96	LYG1	LYG2
LYNX1	LYPD1	LYPD2	LYPD3	LYPD4	LYPD4	LYPD5	LYPD5
LYPD6	LYPD6	LYPD6B	LYPD6B	LYPD8	LYPD8	LYSMD3	LYSMD3
LYSMD4	LYVE1	LYZ	LYZL1	LYZL2	LYZL4	LYZL6	M6PR
MADCAM1	MADCAM1	MAG	MAGEE1	MAGT1	MAL	MAL	MAL2
MALL	MAMDC2	MAMDC4	MAN2B2	MANF	MANSC1	MARCHF1	MARCHF6
MARCKSL1	MARCKSL1	MARCO	MARVELD1	MARVELD1	MARVELD2	MARVELD3	MARVELD3
MAS1	MAS1L	MASP1	MASP2	MATN1	MATN2	MATN3	MATN4
MBL2	MC1R	MC1R	MC2R	MC3R	MC3R	MC4R	MC5R
MCAM	MCEMP1	MCHR1	MCHR1	MCHR2	MCOLN1	MCOLN1	MCOLN2
MCOLN3	MCOLN3	MDGA1	MDGA2	MDK	MDK	MEGF10	MEGF10
MEGF11	MEGF6	MEGF9	MEGF9	MELTF	MELTF	MENT	MENT
MEP1A	MEP1B	MEP1B	MEPE	MERTK	MERTK	MET	METRN
METRNL	METRNL	METTL24	MFAP2	MFAP3	MFAP3L	MFAP3L	MFAP3L
MFAP4	MFAP5	MFGE8	MFGE8	MFGE8	MFGE8	MFRP	MFSD10
MFSD14A	MFSD14B	MFSD14B	MFSD2A	MFSD2A	MFSD2B	MFSD4B	MFSD5
MFSD8	MFSD9	MGAT4A	MGAT5	MGP	MIA	MICA	MICB
MICB	MIF	MILR1	MILR1	MIP	MLANA	MLC1	MLN
MLNR	MME	MMEL1	MMP1	MMP10	MMP11	MMP12	MMP13
MMP14	MMP14	MMP15	MMP15	MMP16	MMP17	MMP19	MMP2
MMP2	MMP20	MMP21	MMP23B	MMP24	MMP25	MMP25	MMP26
MMP28	MMP3	MMP7	MMP8	MMP9	MMP9	MMRN1	MMRN1
MMRN2	MOG	MOG	MOSMO	MPEG1	MPIG6B	MPIG6B	MPL

MPZ	MPZ	MPZL1	MPZL2	MPZL3	MR1	MR1	MRAP
MRAP	MRAP2	MRAS	MRC1	MRC2	MRGPRD	MRGPRD	MRGPRE
MRGPRE	MRGPRF	MRGPRG	MRGPRX1	MRGPRX2	MRGPRX2	MRGPRX3	MRGPRX3
MRGPRX3	MRGPRX4	MS4A1	MS4A10	MS4A12	MS4A2	MS4A3	MS4A4A
MS4A5	MS4A6A	MS4A6E	MS4A7	MS4A8	MSLN	MSLN	MSLN
MSMB	MSMP	MSN	MSR1	MST1	MST1L	MST1R	MSTN
MTNR1A	MTNR1A	MTNR1B	MTRNR2L1	MTRNR2L10	MTRNR2L2	MTRNR2L3	MTRNR2L4
MTRNR2L5	MTRNR2L6	MTRNR2L7	MTRNR2L8	MTRNR2L9	MTUS1	MUC1	MUC1
MUC1	MUC1	MUC1	MUC1	MUC1	MUC1	MUC1	MUC1
MUC13	MUC13	MUC15	MUC15	MUC20	MUC20	MUC20	MUC21
MUC22	MUC4	MUC4	MUC7	MUCL1	MUCL3	MUSK	MXRA8
MYADM	MYADM	MYDGF	MYMK	MYMX	MYOC	MYOF	MZB1
NAALAD2	NAALADL1	NAALADL2	NAALADL2	NAE1	NALCN	NAMPT	NAPSA
NAXD	NAXE	NBEA	NBL1	NCAM1	NCAM1	NCAN	NCEH1
NCKAP1	NCMAP	NCMAP	NCR1	NCR1	NCR2	NCR3	NCR3LG1
NCSTN	NCSTN	NDFIP1	NDFIP1	NDFIP2	NDFIP2	NDNF	NDP
NECTIN1	NECTIN1	NECTIN1	NECTIN1	NECTIN2	NECTIN2	NECTIN3	NECTIN3
NECTIN4	NECTIN4	NEGR1	NELL1	NELL2	NELL2	NENF	NEO1
NETO1	NETO1	NETO2	NEU1	NEU1	NFAM1	NFASC	NFASC
NFASC	NGEF	NGF	NGFR	NGRN	NHLRC3	NIBAN2	NID1
NINJ1	NINJ2	NINJ2	NIPA1	NIPA1	NIPA1	NIPA2	NIPAL1
NIPAL4	NKAIN1	NKAIN1	NKAIN2	NKAIN2	NKAIN3	NKAIN4	NKD2
NKG7	NLGN1	NLGN2	NLGN3	NLGN4X	NLGN4Y	NLGN4Y	NLRP3
NMB	NMBR	NMS	NMS	NMU	NMUR1	NMUR2	NODAL
NOG	NOMO1	NOMO3	NOTCH1	NOTCH2	NOTCH2NLA	NOTCH2NLC	NOTCH3
NOTCH4	NOTUM	NOX1	NOX3	NOX4	NOX5	NPB	NPBWR1
NPBWR1	NPBWR2	NPC1L1	NPC2	NPFF	NPFF	NPFFR1	NPFFR2
NPHS1	NPIPB15	NPNT	NPPA	NPPB	NPPC	NPR1	NPR2
NPR3	NPS	NPS	NPSR1	NPSR1	NPTN	NPTN	NPTX2
NPTXR	NPTXR	NPVF	NPW	NPY	NPY1R	NPY2R	NPY4R
NPY4R2	NPY5R	NPY6R	NRAC	NRAC	NRCAM	NRCAM	NRG1
NRG1	NRG1	NRG1	NRG1	NRG1	NRG1	NRG2	NRG2
NRG2	NRG2	NRG3	NRG3	NRG4	NRN1	NRN1L	NRN1L
NRP1	NRP1	NRP1	NRP1	NRP2	NRP2	NRP2	NRP2
NRROS	NRSN2	NRTN	NRXN1	NRXN2	NRXN3	NRXN3	NRXN3
NSG1	NSUN2	NT5E	NT5E	NT5E	NTF3	NTF4	NTM
NTM	NTN1	NTN3	NTN4	NTN5	NTNG1	NTNG1	NTNG1
NTNG2	NTRK1	NTRK1	NTRK1	NTRK2	NTRK2	NTRK3	NTRK3
NTRK3	NTS	NTSR1	NTSR2	NTSR2	NUCB1	NXPE1	NXPE3
NXPE4	NXPH1	NXPH2	NXPH3	NXPH4	NYX	NYX	OAS1
OBP2A	OBP2B	OC90	OCA2	OCA2	OCLN	OCSTAMP	ODAM
ODAPH	OGN	OIT3	OLFM1	OLFM2	OLFM3	OLFM4	OLFM4
OLFML1	OLFML2A	OLFML2B	OLFML3	OLR1	OLR1	OMD	OMG
OOSP2	OPALIN	OPCML	OPN1LW	OPN1MW	OPN1SW	OPN1SW	OPN3
OPN4	OPN5	OPRD1	OPRK1	OPRL1	OPRM1	OPRPN	OPTC

OR10R2	OR10T2	OR13A1	OR1D2	OR2L13	OR2W3	OR4N2	OR4N2
OR51E1	OR51E2	OR5V1	OR7A5	OR7C1	OR7D2	OR8G1	OR8S1
OR9Q1	ORAI1	ORAI2	ORAI3	ORM1	ORM2	OSCAR	OSCAR
OSCAR	OSCAR	OSCAR	OSCP1	OSM	OSMR	OSMR	OSTC
OSTN	OTOA	OTOA	OTOL1	OTOP1	OTOP1	OTOP2	OTOP3
OTOR	OTOS	OVCH1	OXER1	OXGR1	OXT	OXTR	P2RX1
P2RX2	P2RX2	P2RX3	P2RX4	P2RX4	P2RX5	P2RX5	P2RX5
P2RX6	P2RX6	P2RX7	P2RX7	P2RY1	P2RY10	P2RY11	P2RY12
P2RY12	P2RY12	P2RY12	P2RY13	P2RY13	P2RY14	P2RY14	P2RY2
P2RY2	P2RY2	P2RY4	P2RY4	P2RY6	P2RY6	P2RY6	P2RY8
P2RY8	P2RY8	P3H1	P4HB	PACC1	PAEP	PAG1	PAG1
PAM	PAMR1	PANX1	PANX1	PANX2	PANX2	PANX3	PAPLN
PAPLN	PAPPA	PAQR4	PAQR5	PAQR5	PAQR6	PAQR6	PAQR6
PAQR7	PAQR8	PAQR8	PAQR9	PARD6A	PARM1	PARM1	PATE1
PATE2	PATE3	PCDH1	PCDH1	PCDH10	PCDH10	PCDH11X	PCDH11Y
PCDH12	PCDH15	PCDH17	PCDH18	PCDH19	PCDH20	PCDH20	PCDH7
PCDH8	PCDH8	PCDH9	PCDHA1	PCDHA1	PCDHA10	PCDHA10	PCDHA10
PCDHA11	PCDHA12	PCDHA13	PCDHA2	PCDHA2	PCDHA3	PCDHA4	PCDHA5
PCDHA6	PCDHA6	PCDHA6	PCDHA6	PCDHA7	PCDHA7	PCDHA8	PCDHA9
PCDHAC1	PCDHAC2	PCDHAC2	PCDHB1	PCDHB10	PCDHB11	PCDHB12	PCDHB13
PCDHB14	PCDHB15	PCDHB16	PCDHB16	PCDHB2	PCDHB3	PCDHB4	PCDHB5
PCDHB6	PCDHB7	PCDHB8	PCDHB9	PCDHGA1	PCDHGA10	PCDHGA11	PCDHGA12
PCDHGA2	PCDHGA2	PCDHGA3	PCDHGA4	PCDHGA5	PCDHGA5	PCDHGA6	PCDHGA7
PCDHGA8	PCDHGA8	PCDHGA9	PCDHGA9	PCDHGB1	PCDHGB2	PCDHGB3	PCDHGB4
PCDHGB5	PCDHGB6	PCDHGB6	PCDHGB7	PCDHGC3	PCDHGC3	PCDHGC4	PCDHGC4
PCDHGC5	PCOLCE	PCOLCE2	PCSK1N	PCSK2	PCSK5	PCSK6	PCSK7
PCSK9	PCSK9	PCYOX1	PCYOX1L	PDCD1	PDCD1LG2	PDCD6IP	PDGFA
PDGFB	PDGFB	PDGFC	PDGFD	PDGFRA	PDGFRA	PDGFRA	PDGFRB
PDGFRL	PDGFRL	PDIA6	PDLIM5	PDLIM5	PDLIM5	PDLIM5	PDLIM5
PDPN	PDPN	PDPN	PDPN	PDYN	PDYN	PDZD11	PEAR1
PECAM1	PENK	PERP	PF4	PF4V1	PGA3	PGA4	PGA5
PGAP6	PGC	PGF	PGLYRP1	PGLYRP2	PGLYRP2	PGLYRP3	PGLYRP4
PGRMC1	PHEX	PI15	PI16	PI16	PI16	PI3	PI3
PIANP	PIBF1	PIEZO1	PIEZO1	PIGBOS1	PIGF	PIGO	PIGP
PIGQ	PIGR	PIGR	PIGU	PIGZ	PIK3IP1	PILRA	PILRA
PILRA	PILRA	PILRB	PILRB	PILRB	PINLYP	PIP	PIP4P1
PIP4P2	PKD1L1	PKD1L2	PKD1L2	PKD1L2	PKD1L3	PKD2	PKD2L1
PKD2L2	PKD2L2	PKDCC	PLA1A	PLA2G10	PLA2G12A	PLA2G12B	PLA2G15
PLA2G1B	PLA2G2A	PLA2G2A	PLA2G2C	PLA2G2D	PLA2G2E	PLA2G2E	PLA2G2F
PLA2G3	PLA2G3	PLA2G3	PLA2G5	PLA2G5	PLA2G6	PLA2G7	PLA2R1
PLA2R1	PLA2R1	PLAAT3	PLAC1	PLAC9	PLAT	PLAT	PLAU
PLAUR	PLAUR	PLAUR	PLAUR	PLB1	PLB1	PLB1	PLET1
PLG	PLGLB2	PLGRKT	PLLP	PLOD3	PLP1	PLP2	PLP2
PLPP1	PLPP2	PLPP3	PLPP5	PLPP7	PLPPR5	PLSCR1	PLSCR2
PLSCR2	PLSCR3	PLSCR4	PLSCR4	PLTP	PLVAP	PLVAP	PLXDC1

PLXDC2	PLXDC2	PLXNA1	PLXNA2	PLXNA3	PLXNA4	PLXNA4	PLXNB1
PLXNB2	PLXNB3	PLXNC1	PLXND1	PM20D1	PMCH	PMEL	PMEL
PMP22	PNLDC1	PNLIP	PNLIPRP1	PNLIPRP2	PNLIPRP3	PNOC	PNPLA2
PODN	PODNL1	PODXL	PODXL	PODXL2	POMC	PON1	PON3
POPDC2	POPDC3	PORCN	PORCN	POSTN	POTED	PPBP	PPIA
PPM1L	PPM1L	PPM1L	PPP1R3F	PPT1	PPT1	PPY	PRADC1
PRAP1	PRB2	PRB3	PRCD	PRELP	PRELP	PRF1	PRF1
PRG2	PRH1	PRIMA1	PRKCA	PRKCH	PRL	PRLH	PRLHR
PRLR	PRLR	PRMT8	PRMT8	PRND	PRNP	PROC	PROC
PROCR	PROK1	PROK2	PROKR1	PROKR2	PROM1	PROM1	PROM2
PROS1	PROZ	PRR27	PRR4	PRR7	PRR7	PRRG1	PRRG2
PRRG3	PRRG4	PRRT1	PRRT1	PRRT2	PRRT3	PRSS1	PRSS12
PRSS2	PRSS21	PRSS22	PRSS22	PRSS23	PRSS27	PRSS3	PRSS33
PRSS35	PRSS36	PRSS37	PRSS38	PRSS41	PRSS42P	PRSS48	PRSS53
PRSS54	PRSS55	PRSS57	PRSS58	PRSS8	PRTG	PRTN3	PRXL2A
PSAP	PSAP	PSAPL1	PSCA	PSD2	PSEN1	PSEN2	PSENEN
PSG1	PSG1	PSG11	PSG2	PSG3	PSG4	PSG5	PSG5
PSG6	PSG7	PSG8	PSG9	PSORS1C2	PSPN	PSPN	PTAFR
PTAFR	PTCH1	PTCH1	PTCH2	PTCHD1	PTCRA	PTCRA	PTEN
PTGDR	PTGDR2	PTGDS	PTGER1	PTGER2	PTGER3	PTGER3	PTGER3
PTGER3	PTGER4	PTGFR	PTGFRN	PTGIR	PTH	PTH1R	PTH2
PTH2R	PTHLH	PTK7	PTK7	PTN	PTPRA	PTPRA	PTPRB
PTPRC	PTPRCAP	PTPRD	PTPRE	PTPRF	PTPRG	PTPRH	PTPRJ
PTPRJ	PTPRK	PTPRK	PTPRM	PTPRN	PTPRN	PTPRN2	PTPRN2
PTPRO	PTPRO	PTPRO	PTPRR	PTPRS	PTPRT	PTPRT	PTPRU
PTPRZ1	PTTG1IP	PTX3	PTX4	PVR	PVR	PVRIG	PXDN
PXK	PYM1	PYY	PZP	QPCT	QRFP	QRFP	QRFPR
QRFPR	QSOX1	QSOX2	R3HDML	RABAC1	RAET1E	RAET1E	RAET1G
RAET1G	RAET1L	RAMP1	RAMP2	RAMP3	RARRES2	RBP3	RBP4
RECK	RECK	REEP2	REEP2	REG1A	REG1B	REG3A	REG3G
REG4	RELL1	RELL2	RELT	REN	RESP18	RET	RET
RETN	RETNLB	RFTN2	RGMA	RGMB	RGMB	RGMB	RGR
RGS9BP	RHAG	RHBDD1	RHBDD2	RHBDD2	RHBDF2	RHBDL2	RHBG
RHBG	RHCE	RHCG	RHD	RHD	RHEB	RHEX	RHO
RHO	RIPOR2	RLN1	RLN2	RLN3	RNASE1	RNASE1	RNASE10
RNASE11	RNASE12	RNASE13	RNASE3	RNASE4	RNASE6	RNASE6	RNASE7
RNASE8	RNASE9	RNASET2	RNF130	RNF130	RNF144A	RNF150	RNF150
RNF180	RNF180	RNF215	RNF43	RNF43	RNFT2	RNLS	RNPEP
RNPEP	ROBO1	ROBO2	ROBO3	ROBO4	ROR1	ROR1	ROR2
ROS1	RPSA	RPTN	RRAS2	RRH	RS1	RS1	RSPO1
RSPO2	RSPO3	RSPO3	RSPO4	RSPRY1	RTBDN	RTL8C	RTN4
RTN4R	RTN4R	RTN4R	RTN4RL1	RTN4RL2	RTP1	RTP1	RTP2
RXFP1	RXFP1	RXFP1	RXFP2	RXFP3	RXFP4	RXYLT1	RYK
RYK	S100A12	S100A13	S100A7	S100A7	S100A8	S100A8	S100A9
S100A9	S1PR1	S1PR2	S1PR3	S1PR4	S1PR5	S1PR5	S1PR5

SAA1	SAA2	SAA4	SAMD1	SBSN	SBSPON	SCAMP5	SCAMP5
SCARA3	SCARA3	SCARA5	SCARA5	SCARB1	SCARB1	SCARF1	SCARF2
SCG2	SCG3	SCG5	SCG5	SCGB1A1	SCGB1C1	SCGB1C2	SCGB1D1
SCGB1D2	SCGB1D4	SCGB2A1	SCGB2A2	SCGB2B2	SCGB3A1	SCGB3A2	SCGN
SCGN	SCIMP	SCN10A	SCN1B	SCN1B	SCN1B	SCN1B	SCN2A
SCN2B	SCN3A	SCN3B	SCN4A	SCN4B	SCN4B	SCN5A	SCN5A
SCN7A	SCN9A	SCNN1A	SCNN1B	SCNN1D	SCNN1D	SCNN1G	SCPEP1
SCRG1	SCRG1	SCT	SCT	SCTR	SCUBE1	SCUBE1	SCUBE2
SCUBE3	SDC1	SDC1	SDC2	SDC3	SDC3	SDC4	SDC4
SDCBP	SDCBP	SDCBP	SDF2	SDK1	SDK2	SEC23B	SEC61G
SECTM1	SECTM1	SELE	SELENOK	SELENOP	SELENOW	SELL	SELL
SELL	SELP	SELP	SELPLG	SEMA3A	SEMA3A	SEMA3B	SEMA3C
SEMA3C	SEMA3D	SEMA3E	SEMA3F	SEMA3G	SEMA4A	SEMA4B	SEMA4C
SEMA4D	SEMA4F	SEMA4G	SEMA5A	SEMA5B	SEMA6A	SEMA6B	SEMA6C
SEMA6D	SEMA6D	SEMA7A	SEMG1	SEMG2	SERBP1	SERBP1	SERINC1
SERINC3	SERINC5	SERP1	SERPINA1	SERPINA10	SERPINA10	SERPINA11	SERPINA12
SERPINA3	SERPINA4	SERPINA5	SERPINA5	SERPINA6	SERPINA6	SERPINA7	SERPINA9
SERPINA9	SERPINB1	SERPINB2	SERPINB2	SERPINB3	SERPINB4	SERPINB5	SERPINB5
SERPINC1	SERPINC1	SERPINE1	SERPINE1	SERPINE2	SERPINE2	SERPINE3	SERPINF1
SERPINF2	SERPING1	SERPINI2	SEZ6	SEZ6L	SEZ6L2	SFN	SFRP1
SFRP2	SFRP2	SFRP4	SFRP4	SFRP5	SFTA2	SFTA3	SFTPA1
SFTPA2	SFTPB	SFTPC	SFTPC	SFTPD	SGCA	SGCB	SGCD
SGCD	SGCD	SGCE	SGCG	SGCZ	SGCZ	SGMS2	SGPP1
SHBG	SHISA2	SHISA4	SHISA5	SHISA7	SHISA8	SHISA9	SHISAL1
SIAE	SIDT1	SIDT2	SIGIRR	SIGLEC1	SIGLEC10	SIGLEC10	SIGLEC11
SIGLEC11	SIGLEC11	SIGLEC12	SIGLEC14	SIGLEC15	SIGLEC5	SIGLEC6	SIGLEC6
SIGLEC6	SIGLEC7	SIGLEC8	SIGLEC9	SIGLEC9	SIGLECL1	SIGMAR1	SIRPA
SIRPA	SIRPA	SIRPB1	SIRPB1	SIRPB2	SIRPD	SIRPD	SIRPG
SIT1	SLAMF1	SLAMF1	SLAMF6	SLAMF7	SLAMF7	SLAMF7	SLAMF7
SLAMF7	SLAMF7	SLAMF7	SLAMF7	SLAMF7	SLAMF7	SLAMF7	SLAMF7
SLAMF8	SLAMF9	SLC10A1	SLC10A2	SLC10A4	SLC10A5	SLC10A6	SLC11A1
SLC11A2	SLC11A2	SLC12A1	SLC12A2	SLC12A3	SLC12A6	SLC12A7	SLC12A8
SLC12A9	SLC12A9	SLC13A1	SLC13A2	SLC13A2	SLC13A3	SLC13A3	SLC13A4
SLC13A5	SLC14A1	SLC14A2	SLC15A1	SLC15A2	SLC15A3	SLC16A1	SLC16A1
SLC16A10	SLC16A10	SLC16A11	SLC16A12	SLC16A13	SLC16A14	SLC16A2	SLC16A3
SLC16A4	SLC16A4	SLC16A5	SLC16A6	SLC16A7	SLC16A8	SLC17A1	SLC17A3
SLC17A4	SLC17A5	SLC17A7	SLC18A1	SLC18A2	SLC18B1	SLC19A1	SLC19A1
SLC19A2	SLC19A3	SLC1A1	SLC1A2	SLC1A3	SLC1A4	SLC1A5	SLC1A6
SLC1A6	SLC1A7	SLC1A7	SLC20A1	SLC20A2	SLC22A1	SLC22A1	SLC22A10
SLC22A11	SLC22A12	SLC22A12	SLC22A13	SLC22A13	SLC22A14	SLC22A16	SLC22A17
SLC22A18	SLC22A2	SLC22A23	SLC22A23	SLC22A24	SLC22A25	SLC22A31	SLC22A4
SLC22A5	SLC22A6	SLC22A6	SLC22A7	SLC22A8	SLC22A9	SLC22A9	SLC22A9
SLC23A1	SLC23A1	SLC23A1	SLC23A2	SLC23A2	SLC23A3	SLC23A3	SLC24A1
SLC24A3	SLC24A4	SLC24A4	SLC24A5	SLC26A1	SLC26A11	SLC26A11	SLC26A2
SLC26A3	SLC26A4	SLC26A5	SLC26A5	SLC26A5	SLC26A5	SLC26A6	SLC26A7

SLC26A8	SLC26A9	SLC27A1	SLC27A2	SLC27A3	SLC27A4	SLC27A5	SLC27A6
SLC28A1	SLC28A1	SLC28A1	SLC28A2	SLC28A3	SLC29A1	SLC29A2	SLC29A3
SLC29A4	SLC2A1	SLC2A10	SLC2A11	SLC2A12	SLC2A13	SLC2A13	SLC2A14
SLC2A2	SLC2A2	SLC2A3	SLC2A4	SLC2A5	SLC2A5	SLC2A6	SLC2A7
SLC2A8	SLC2A9	SLC2A9	SLC2A9	SLC30A1	SLC30A10	SLC30A5	SLC30A5
SLC30A7	SLC30A8	SLC30A8	SLC31A1	SLC31A1	SLC31A2	SLC33A1	SLC34A1
SLC34A2	SLC34A3	SLC35D2	SLC35E3	SLC35F2	SLC35G1	SLC35G2	SLC36A1
SLC36A1	SLC36A2	SLC36A2	SLC36A3	SLC36A3	SLC36A4	SLC38A1	SLC38A2
SLC38A3	SLC38A4	SLC38A5	SLC38A6	SLC38A6	SLC38A7	SLC38A7	SLC38A8
SLC39A1	SLC39A11	SLC39A12	SLC39A14	SLC39A14	SLC39A2	SLC39A3	SLC39A3
SLC39A4	SLC39A4	SLC39A5	SLC39A5	SLC39A6	SLC39A6	SLC39A6	SLC39A8
SLC39A8	SLC39A9	SLC39A9	SLC3A1	SLC3A2	SLC3A2	SLC3A2	SLC3A2
SLC40A1	SLC40A1	SLC41A1	SLC41A2	SLC41A2	SLC41A3	SLC41A3	SLC43A1
SLC43A2	SLC43A2	SLC43A3	SLC44A1	SLC44A2	SLC44A3	SLC44A3	SLC44A4
SLC44A4	SLC44A5	SLC45A1	SLC45A2	SLC45A2	SLC45A3	SLC46A1	SLC46A1
SLC46A2	SLC46A3	SLC46A3	SLC47A1	SLC47A1	SLC47A2	SLC4A1	SLC4A10
SLC4A11	SLC4A2	SLC4A2	SLC4A2	SLC4A4	SLC4A5	SLC4A7	SLC4A8
SLC4A8	SLC50A1	SLC50A1	SLC51A	SLC51A	SLC51B	SLC51B	SLC52A1
SLC52A2	SLC52A3	SLC5A1	SLC5A1	SLC5A10	SLC5A10	SLC5A11	SLC5A12
SLC5A12	SLC5A2	SLC5A3	SLC5A4	SLC5A5	SLC5A6	SLC5A7	SLC5A8
SLC5A9	SLC6A1	SLC6A11	SLC6A11	SLC6A12	SLC6A13	SLC6A13	SLC6A14
SLC6A15	SLC6A15	SLC6A15	SLC6A16	SLC6A16	SLC6A17	SLC6A18	SLC6A19
SLC6A2	SLC6A20	SLC6A3	SLC6A4	SLC6A5	SLC6A6	SLC6A6	SLC6A7
SLC6A7	SLC6A8	SLC6A9	SLC7A1	SLC7A10	SLC7A11	SLC7A13	SLC7A2
SLC7A2	SLC7A3	SLC7A4	SLC7A5	SLC7A6	SLC7A7	SLC7A8	SLC7A8
SLC7A9	SLC8A1	SLC8A2	SLC8A3	SLC8A3	SLC8B1	SLC9A1	SLC9A1
SLC9A2	SLC9A3	SLC9A3R1	SLC9A4	SLC9A5	SLC9A6	SLC9A6	SLC9A7
SLC9B1	SLC9B2	SLCO1A2	SLCO1A2	SLCO1A2	SLCO1B1	SLCO1B3	SLCO1B7
SLCO1C1	SLCO2A1	SLCO2B1	SLCO3A1	SLCO3A1	SLCO4A1	SLCO4C1	SLCO5A1
SLCO5A1	SLCO6A1	SLCO6A1	SLIT1	SLIT2	SLIT3	SLITRK1	SLITRK1
SLITRK2	SLITRK3	SLITRK4	SLITRK5	SLITRK6	SLMAP	SLPI	SLURP1
SLURP2	SMAGP	SMIM1	SMIM23	SMIM29	SMIM7	SMIM9	SMO
SMOC1	SMOC2	SMPD1	SMPD1	SMPD2	SMPD3	SMPDL3A	SMPDL3B
SMR3A	SMR3B	SNAP23	SNCA	SNCA	SNED1	SNORC	SOD3
SORBS1	SORBS1	SORCS2	SORD	SORD	SORL1	SORT1	SOST
SOSTDC1	SPA17	SPACA1	SPACA3	SPACA4	SPACA5	SPACA6	SPACA7
SPAG11B	SPAM1	SPAM1	SPARC	SPARCL1	SPATA20	SPATA6	SPCS1
SPG11	SPG11	SPINK1	SPINK13	SPINK14	SPINK2	SPINK2	SPINK4
SPINK5	SPINK6	SPINK7	SPINK8	SPINK9	SPINT1	SPINT1	SPINT1
SPINT2	SPINT3	SPINT4	SPN	SPNS2	SPNS3	SPOCK1	SPOCK2
SPOCK3	SPON1	SPON2	SPP1	SPPL2B	SPRED1	SPRN	SPX
SRGN	SRGN	SRI	SRPX	SRPX2	SSC4D	SSC5D	SSPN
SSPN	SST	SSTR1	SSTR1	SSTR2	SSTR3	SSTR4	SSTR5
ST14	ST3GAL1	ST3GAL1	ST3GAL2	ST3GAL3	ST3GAL4	ST6GAL1	ST6GAL1
ST6GALNAC6	ST7	STAB2	STARD3	STARD3	STARD3NL	STATH	STBD1

STC1	STC2	STEAP1	STEAP1B	STEAP2	STEAP4	STEAP4	STIM1
STIM2	STING1	STOM	STOML1	STOML3	STRA6	STX1A	STX1A
STX3	STX4	STXBP3	STYK1	SUCNR1	SULF1	SUSD1	SUSD1
SUSD2	SUSD3	SUSD4	SUSD4	SUSD4	SUSD5	SUSD6	SV2A
SV2A	SV2B	SV2C	SVBP	SYNDIG1	SYNDIG1	SYNDIG1L	SYNGR2
SYNGR4	SYT14	SYT14	SYT15	SYT15	SYT3	SYT6	SYT7
SYT8	TAAR1	TAAR2	TAAR2	TAAR3P	TAAR5	TAAR6	TAAR8
TAAR9	TAC1	TAC1	TAC3	TAC4	TACR1	TACR1	TACR2
TACR3	TACSTD2	TAFA1	TAFA2	TAFA3	TAFA4	TAFA5	TAGLN2
TAOK3	TAPBPL	TAPT1	TARM1	TAS1R1	TAS1R1	TAS1R2	TAS1R3
TAS2R1	TAS2R10	TAS2R13	TAS2R14	TAS2R16	TAS2R19	TAS2R20	TAS2R20
TAS2R3	TAS2R30	TAS2R31	TAS2R38	TAS2R39	TAS2R4	TAS2R40	TAS2R41
TAS2R42	TAS2R43	TAS2R45	TAS2R46	TAS2R5	TAS2R50	TAS2R60	TAS2R7
TAS2R7	TAS2R8	TAS2R9	TBXA2R	TCN1	TCN2	TCTA	TCTN1
TCTN2	TCTN2	TCTN3	TDGF1	TDGF1	TECTA	TECTB	TECTB
TEK	TEK	TEK	TENM1	TEPP	TEX101	TEX101	TEX264
TEX29	TF	TF	TFF1	TFF2	TFF3	TFPI	TFPI
TFPI	TFPI2	TFPI2	TFR2	TFR2	TFRC	TFRC	TG
TGFA	TGFA	TGFA	TGFB1	TGFB2	TGFB3	TGFBI	TGFBI
TGFBR1	TGFBR1	TGFBR2	TGFBR2	TGFBR3	TGFBR3	TGFBR3L	TGM2
TGM2	TGOLN2	TGOLN2	THBD	THBS1	THBS4	THEM6	THNSL2
THOP1	THPO	THSD1	THSD4	THSD7A	THY1	THY1	TICAM2
TIE1	TIGIT	TIMD4	TIMP1	TIMP2	TIMP3	TIMP4	TINAG
TINAGL1	TIRAP	TLCD1	TLCD2	TLCD3A	TLL1	TLR1	TLR10
TLR2	TLR3	TLR4	TLR5	TLR6	TLR6	TLR7	TLR8
TLR9	TM2D1	TM4SF1	TM4SF1	TM4SF18	TM4SF19	TM4SF4	TM4SF5
		TM7SF3	TM9SF2	TMC1	TMC2	TMC3	TMC4
TMC4	TMC5	TMC5	TMC7	TMDD1	TMED1	TMED10	TMEFF1
TMEFF2	TMEFF2	TMEM100	TMEM102	TMEM106A	TMEM108	TMEM114	TMEM117
TMEM119	TMEM120A	TMEM123	TMEM127	TMEM132A	TMEM132B	TMEM132C	TMEM132D
TMEM132E	TMEM134	TMEM139	TMEM140	TMEM147	TMEM150A	TMEM150A	TMEM150A
TMEM150B	TMEM150C	TMEM154	TMEM155	TMEM156	TMEM161A	TMEM163	TMEM167A
TMEM167B	TMEM169	TMEM17	TMEM170B	TMEM178A	TMEM182	TMEM182	TMEM184A
TMEM184B	TMEM190	TMEM198	TMEM198	TMEM200A	TMEM200C	TMEM204	TMEM205
TMEM210	TMEM213	TMEM219	TMEM222	TMEM222	TMEM222	TMEM229B	TMEM231
TMEM233	TMEM238	TMEM240	TMEM241	TMEM25	TMEM25	TMEM25	TMEM256
TMEM266	TMEM273	TMEM30A	TMEM30B	TMEM39A	TMEM39A	TMEM44	TMEM44
TMEM47	TMEM50A	TMEM59	TMEM63A	TMEM63B	TMEM63C	TMEM65	TMEM67
TMEM79	TMEM79	TMEM81	TMEM86A	TMEM86B	TMEM86B	TMEM88	TMEM89
TMEM8B	TMEM8B	TMEM9	TMEM91	TMEM92	TMEM95	TMEM97	TMEM97
TMEM98	TMIE	TMIGD1	TMIGD2	TMIGD3	TMPRSS11A	TMPRSS11A	TMPRSS11B
TMPRSS11D	TMPRSS11D	TMPRSS11E	TMPRSS11E	TMPRSS11F	TMPRSS12	TMPRSS13	TMPRSS15
TMPRSS2	TMPRSS2	TMPRSS3	TMPRSS3	TMPRSS4	TMPRSS4	TMPRSS5	TMPRSS6
TMPRSS6	TMPRSS7	TMPRSS7	TMPRSS9	TMUB1	TMX1	TMX2	TMX2
TNC	TNF	TNF	TNFRSF10A	TNFRSF10B	TNFRSF10C	TNFRSF10D	TNFRSF11A

TNFRSF11B	TNFRSF12A	TNFRSF13B	TNFRSF13B	TNFRSF13C	TNFRSF14	TNFRSF14	TNFRSF17
TNFRSF18	TNFRSF18	TNFRSF18	TNFRSF18	TNFRSF19	TNFRSF19	TNFRSF1A	TNFRSF1A
TNFRSF1B	TNFRSF1B	TNFRSF1B	TNFRSF21	TNFRSF25	TNFRSF25	TNFRSF25	TNFRSF25
TNFRSF25	TNFRSF25	TNFRSF25	TNFRSF4	TNFRSF6B	TNFRSF6B	TNFRSF8	TNFRSF9
TNFSF10	TNFSF11	TNFSF11	TNFSF11	TNFSF12	TNFSF12	TNFSF12	TNFSF13
TNFSF13	TNFSF13B	TNFSF13B	TNFSF14	TNFSF14	TNFSF15	TNFSF15	TNFSF18
TNFSF18	TNFSF4	TNFSF8	TNFSF9	TNMD	TNN	TOR2A	TP53I11
TP53I13	TPBG	TPBGL	TPCN1	TPCN1	TPCN2	TPO	TPSAB1
TPSB2	TPSB2	TPSD1	TRABD2A	TRABD2A	TRABD2B	TRAF3IP3	TRAF3IP3
TRARG1	TRAT1	TRAT1	TRAV20	TRBC1	TRBC2	TRDV1	TRDV2
TRDV2	TREH	TREH	TREM1	TREM1	TREM2	TREM2	TREM2
TREML1	TREML2	TREML2	TREML4	TRGV9	TRH	TRHDE	TRHR
TRIL	TRIM72	TRO	TRPA1	TRPC1	TRPC4	TRPC5	TRPC6
TRPC7	TRPM2	TRPM3	TRPM4	TRPM5	TRPM6	TRPM7	TRPM8
TRPV1	TRPV2	TRPV3	TRPV4	TRPV4	TRPV5	TRPV5	TRPV6
TSHB	TSHR	TSKU	TSLP	TSPAN1	TSPAN10	TSPAN11	TSPAN12
TSPAN13	TSPAN14	TSPAN14	TSPAN15	TSPAN16	TSPAN17	TSPAN18	TSPAN19
TSPAN2	TSPAN3	TSPAN31	TSPAN32	TSPAN32	TSPAN33	TSPAN33	TSPAN4
TSPAN4	TSPAN5	TSPAN6	TSPAN7	TSPAN8	TSPAN9	TSPEAR	TTR
TTYH1	TTYH1	TTYH2	TTYH2	TTYH3	TTYH3	TUB	TUFT1
TULP1	TULP2	TULP3	TWSG1	TXN	TXNDC15	TXNDC16	TYRO3
TYROBP	TYROBP	UBAC1	UBE2B	UBL3	UCMA	UCMA	UCN
UCN2	UCN3	UGT2A1	UGT2A3	UGT3A1	UGT3A1	UGT3A2	ULBP1
ULBP2	ULBP2	ULBP3	UMOD	UMOD	UMODL1	UNC5A	UNC5B
UNC5C	UNC5CL	UNC5D	UNC93A	UPK1A	UPK1B	UPK2	UPK3A
UPK3B	UPK3B	UPK3BL1	UPK3BL1	UTS2	UTS2	UTS2B	UTS2R
VAMP2	VAMP5	VAMP8	VANGL1	VANGL2	VAPA	VASH1	VASH2
VASN	VASN	VASP	VCAM1	VCL	VDAC1	VEGFA	VEGFA
VEGFA	VEGFB	VEGFC	VEGFD	VGF	VIP	VIPR1	VIPR1
VIPR2	VIPR2	VIT	VLDLR	VLDLR	VMO1	VMP1	VMP1
VN1R1	VN1R2	VN1R3	VN1R3	VN1R4	VN1R5	VNN1	VNN2
VNN3	VOPP1	VPREB1	VSIG1	VSIG10	VSIG2	VSIG4	VSIG8
VSIR	VSTM1	VSTM1	VSTM1	VSTM2A	VSTM2B	VSTM4	VSTM5
VTCN1	VTCN1	VTCN1	VTI1A	VTN	VWA1	VWA2	VWA3A
VWA5B1	VWA7	VWC2	VWC2L	VWCE	VWDE	VWF	WFDC1
WFDC10A	WFDC10B	WFDC11	WFDC11	WFDC12	WFDC13	WFDC2	WFDC3
WFDC5	WFDC6	WFDC8	WFDC9	WFDC9	WFIKKN1	WFIKKN2	WIF1
WLS	WNT1	WNT10A	WNT10B	WNT11	WNT16	WNT2	WNT2
WNT2B	WNT3	WNT3A	WNT3A	WNT4	WNT4	WNT5A	WNT5A
WNT5B	WNT5B	WNT6	WNT7A	WNT7A	WNT7B	WNT7B	WNT8A
WNT8B	WNT9A	WNT9A	WNT9B	WNT9B	WWP1	XCL1	XCL1
XCL2	XCR1	XG	XG	XK	XKR3	XKR4	XKR8
XKRX	XPNPEP2	XPR1	XYLT1	XYLT2	YBX1	YIF1B	YIF1B
YIPF3	YKT6	ZACN	ZACN	ZDHHC17	ZDHHC2	ZDHHC20	ZDHHC3
ZDHHC4	ZDHHC5	ZDHHC5	ZFYVE27	ZG16	ZG16	ZG16B	ZMYND19

ZNRF3	ZP1	ZP1	ZP2	ZP2	ZP3	ZP3	ZP3
ZP4	ZP4	ZPBP	ZPBP2	ZPLD1	ZPLD1	ZPLD1	
**Heterodimers (396 pairs)**							
ACKR3: CXCR4	ACKR4: CXCR3	ACVR1: TGFBR	ACVR1: TGFBR	ACVR1: BMPR2	ACVR1: ACVR2	ACVR1: ACVR2	ACVR1: AMHR2
ACVR1B: TGFB	ACVR1B: TGFB	ACVR1B: BMPF	ACVR1B: ACVF	ACVR1B: ACVF	ACVR1B: AMHI	ACVR1B: TGFB	ACVR1B: TGFBR2
ACVR1B: BMPF	ACVR1B: ACVF	ACVR1B: ACVF	ACVR1B: AMHI	ACVR1C: TGFB	ACVR1C: TGFB	ACVR1C: BMPF	ACVR1C: ACVR2A
ACVR1C: ACVF	ACVR1C: AMHI	ACVRL1: TGFB	ACVRL1: TGFB	ACVRL1: BMPF	ACVRL1: ACVF	ACVRL1: ACVF	ACVRL1: AMHR2
ADIPOR1: ADIF	ADRA1A: ADRA	AMIGO1: AMIG	AMIGO1: AMIG	AMIGO2: AMIG	AOC2: AOC3	ASIC1 (1a): ASI	ASIC1 (1a): ASIC2 (2b)
ASIC1 (1a): ASI	ASIC1 (1a): ASI	ASIC1 (1a): ASI	ASIC2 (2a): ASI	ATP1A1: ATP1E	ATP1A1: ATP1E	ATP1A1: ATP1E	ATP1A2: ATP1B1
ATP1A2: ATP1E	ATP1A2: ATP1E	ATP1A3: ATP1E	ATP1A3: ATP1E	ATP1A3: ATP1E	ATP4A: ATP4B	BMPR1A: TGFB	BMPR1A: TGFBR2
BMPR1A: BMPF	BMPR1A: ACVF	BMPR1A: ACVF	BMPR1A: AMHI	BMPR1B: TGFB	BMPR1B: TGFB	BMPR1B: BMPF	BMPR1B: ACVR2A
BMPR1B: ACVF	BMPR1B: AMHI	BSG (Iso 2):BSG	BSND: CLCNKE	CD19: CD81	CD1A: B2M	CD1B: B2M	CD1C: B2M
CD1D: B2M	CD1E: B2M	CD22: CD22	CD22: CD22	CD3D: CD3E	CD3G: CD3E	CD79A: CD79B	CD8A: CD8B
CD8A: CD8B2	CEACAM8:CEA	CHRNA2: CHRN	CHRNA2: CHRN	CHRNA3: CHRN	CHRNA3: CHRN	CHRNA4: CHRN	CHRNA4: CHRNB4
CHRNA9: CHRN	CLEC4E: CLEC4	CNGA1: CNGB1	CNGA3: CNGB3	CNTNAP1: CNT	CSF2RA: CSF2R	CYBA: CYBB	DPP6: KCND2
DRD1: DRD2	DRD2: DRD4	DUOX2: DUOX	EFNA1: EPHA2	EGFR: ERBB2	EGFR: ERBB3	EGFR: ERBB4	ENO1: ENO2
ERBB2: ERBB3	ERBB2: ERBB4	ERBB3: ERBB4	FAP: DPP4	FCER1G: LILRA	FCGR3A: FCER	FCGR3A: CD24	FCGRT: B2M
GABBR1: GABE	GABBR1: GABE	GFRAL: TGFBR	GFRAL: ENG	GFRAL: CFC1	GFRAL: TDGF1	GFRAL: RGMA	GFRAL: RGMB
GFRAL: HJV	GIPR: GLP1R	GJA3: GJA8	GJB2: GJB4	GLRA1: GLRB	GLRA2: GLRB	GLRA3: GLRB	GNB1: GNG2
GP1BA: GP1BB	GP1BA: GP1BB	GPER1: CRHR1	GPER1: HTR1A	GPER1: PAQR8	GPR135: MTNR	GPR183: CXCR5	GPR50: MTNR1A
GPR50: MTNR1	GPR61: MTNR1	GPR62: MTNR1	GRIK1: GRIK5	GRIK2: GRIK5	GRIK3: GRIK5	GRIK4: GRIK1	GRIK4: GRIK3
GRIN1: GRIN2A	GRIN1: GRIN2B	GRIN1: GRIN2C	GRIN1: GRIN2D	HCN1: HCN2	HCN1: HCN3	HCN1: HCN3	HCN1: HCN4
HCN2: HCN4	HLA-A: B2M	HLA-A: B2M	HLA-A: B2M	HLA-B: B2M	HLA-C: B2M	HLA-DOA: HLA	HLA-DPA1: HLA-DPB1
HLA-DPA1: HL	HLA-DQA1: HL	HLA-DQA2: HL	HLA-DRA: HLA	HLA-DRA: HLA	HLA-DRA: HLA	HLA-DRA: HLA	HLA-E: B2M
HLA-F: B2M	HLA-G: B2M	HLA-G: B2M	HLA-H: B2M	HTR1D: HTR1B	HTR3B: HTR3A	HTR3C: HTR3A	HTR3D: HTR3A
HTR3E: HTR3A	IFNAR1: IFNAR	IFNGR1: IFNGR	IFNLR1: IL10RE	IL10RA: IL10RE	IL12RB1: IL12R	IL17RA: IL17RE	IL17RA: IL17RC
IL17RA: IL17RD	IL17RA: IL17RE	IL18R1: IL18RA	IL1R2: IL1RAP	IL1RL2: IL1RAF	IL20RA: IL20RE	IL20RA: IL10RE	IL21R: IL2RG
IL22RA1: IL10R	IL22RA1: IL20R	IL23R: IL12RB1	IL27RA: IL6ST	IL2RB: IL2RG	IL31RA: OSMR	IL3RA: CSF2RB	IL4R: IL2RG
IL4R: IL13RA1	IL5RA: CSF2RB	IL6ST: IL6R	IL6ST: LIFR	IL6ST: OSMR	IL7R: IL2RG	IL7R: CRLF2	IL9R: IL2RG
INSR: IGF1R	ITGA10: ITGB1	ITGA11: ITGB1	ITGA2: ITGB1	ITGA2B: ITGB3	ITGA3: ITGB1	ITGA4: ITGB1	ITGA4: ITGB7
ITGA5: ITGB1	ITGA6: ITGB1	ITGA6: ITGB4	ITGA7: ITGB1	ITGA8: ITGB1	ITGA9: ITGB1	ITGAD: ITGB2	ITGAE: ITGB7
ITGAL: ITGB2	ITGAM: ITGB2	ITGAV: ITGB1	ITGAV: ITGB3	ITGAV: ITGB5	ITGAV: ITGB6	ITGAV: ITGB8	ITGAX: ITGB2
KCNA1: KCNA2	KCNA1: KCNA4	KCNA1: KCNA5	KCNA1: KCNA6	KCNA2: KCNA4	KCNA2: KCNA5	KCNA2: KCNA6	KCNA4: KCNA5
KCNA4: KCNA6	KCNA5: KCNA6	KCNB1: KCNG1	KCNB1: KCNG3	KCNB1: KCNG4	KCNB1: KCNH1	KCNB1: KCNH2	KCNB1: KCNS1
KCNB1: KCNS2	KCNB1: KCNS3	KCNB1: KCNV1	KCND1: KCND2	KCND2: KCNIP	KCNE2: KCNQ1	KCNH1: KCNH5	KCNH2: KCNH6
KCNH2: KCNE1	KCNH2: KCNE2	KCNH7: KCNH2	KCNH7: KCNH6	KCNJ10: KCNJ1	KCNJ12: KCNJ4	KCNJ2: KCNJ4	KCNJ2: KCNJ16
KCNJ3: KCNJ6	KCNJ3: KCNJ9	KCNJ3: KCNJ5	KCNJ6: KCNJ5	KCNK1: KCNK9	KCNQ2: KCNQ3	KCNQ4: KCNQ3	KCNQ5: KCNQ3
KCNQ5: KCNQ1	KDR: FLT1	KDR: FLT4	KEL: XK	KIRREL3: NPHS	KLRC1: KLRD1	KLRC2: KLRD1	KLRC2: KLRD1
KLRC3: KLRD1	KLRC4: KLRD1	KLRK1: HCST	LRFN1: LRFN2	LRFN1: LRFN3	LRFN1: LRFN4	LRFN1: LRFN5	LRFN2: LRFN3
LRFN2: LRFN4	LRFN2: LRFN5	LRFN3: LRFN4	LRFN3: LRFN5	LRFN4: LRFN5	LTA: LTB	MCOLN1: MCO	MCOLN1: MCOLN3
MCOLN3: TRPV	MEP1A: MEP1B	MOG: MOG	MR1: B2M	MRAP: MRAP2	NDFIP1: NDFIP2	NLGN1: NLGN2	NLGN1: NLGN3
NLGN1: NRXN1	NLGN2: NLGN3	NRP1: NRP2	NRP1: PLXNA1	NRP1: PLXNA2	NRP1: PLXNA4	NRP1: KDR	OPRM1: OPRD1
OPRM1: OPRK1	OPRM1: OPRL1	OPRM1: ADRA2	OPRM1: SSTR2	OPRM1: CNR1	OPRM1: CCR5	P2RX1: P2RX2	P2RX1: P2RX3
P2RX1: P2RX4	P2RX1: P2RX5	P2RX1: P2RX6	P2RX2: P2RX3	P2RX2: P2RX4	P2RX2: P2RX5	P2RX2: P2RX6	P2RX3: P2RX4
P2RX3: P2RX5	P2RX3: P2RX6	P2RX4: P2RX5	P2RX4: P2RX6	P2RX5: P2RX6	PANX1: PANX2	PDGFRA: PDGF	PKD1L1: PKD2L1

PLXNB1: PLXN	RAMP1: CALCR	RAMP2: CALCR	RAMP3: CALCR	SCN1B: SCN4A	SCN1B: SCN5A	SCN1B: SCN9A	SCN1B: SCN10A
SCN2B: SCN5A	SCN2B: SCN9A	SCN2B: SCN10A	SCN3B: SCN5A	SCN3B: SCN9A	SCN3B: SCN10A	SCN4B: SCN5A	SGCB: SGCD
SLC3A1: SLC7A	SLC3A2: SLC7A	SLC3A2: SLC7A	SLC3A2: SLC7A	SLC3A2: SLC7A	SLC3A2: SLC7A	SLC3A2: SLC7A	SLC51A: SLC51B
SSTR2: SSTR3	SSTR2: SSTR5	STIM1: STIM2	TAS1R1: TAS1R	TAS1R2: TAS1R	TEK: TIE1	TGFBR1: TGFBI	TGFBR1: TGFBR2
TGFBR1: BMPR	TGFBR1: ACVR	TGFBR1: ACVR	TGFBR1: AMHR	TGFBR1: TGFBI	TGFBR1: TGFBI	TGFBR1: BMPR	TGFBR1: ACVR2A
TGFBR1: ACVR	TGFBR1: AMHR	TGFBR1: ENG	TGFBR2: ENG	TIGIT: PVR	TLR2: TLR1	TLR2: TLR6	TMPRSS11E: SERPINA5
TMPRSS11E: SE	TMPRSS7: SERI	TRGV9: TRDV1	TRGV9: TRDV2	TRPC1: TRPC4	TRPC1: TRPC5	TRPC4: TRPC5	TRPV1: TRPV3
TRPV5: TRPV6	UPK1B: UPK3A	UPK1B: UPK3B	VANGL1: VANGL2				
