# Supplementary material for: Nonclinical characterization of ICVB-1042 as a selective oncolytic adenovirus for solid tumor treatment
Source: Commun Biol. 2024 Sep 13;7:1132. doi: 10.1038/s42003-024-06839-6 (PMC11399272; doi:10.1038/s42003-024-06839-6)
Supplement: Supplementary file 10 — Reporting Summary [file 42003_2024_6839_MOESM10_ESM.pdf]

nature portfolio

Corresponding author(s): Heba Nowyhed

Last updated by author(s): Aug 12, 2024

Reporting Summary

Nature Portfolio wishes to improve the reproducibility of the work that we publish. This form provides structure for consistency and transparency in reporting. For further information on Nature Portfolio policies, see our Editorial Policies and the Editorial Policy Checklist.

Please do not complete any field with "not applicable" or n/a. Refer to the help text for what text to use if an item is not relevant to your study.
For final submission: please carefully check your responses for accuracy; you will not be able to make changes later.

Statistics

For all statistical analyses, confirm that the following items are present in the figure legend, table legend, main text, or Methods section.

n/a	Confirmed	
☐	☑	The exact sample size (n) for each experimental group/condition, given as a discrete number and unit of measurement
☐	☑	A statement on whether measurements were taken from distinct samples or whether the same sample was measured repeatedly
☐	☑	The statistical test(s) used AND whether they are one- or two-sided<br>*Only common tests should be described solely by name; describe more complex techniques in the Methods section.*
☑	☐	A description of all covariates tested
☑	☐	A description of any assumptions or corrections, such as tests of normality and adjustment for multiple comparisons
☐	☑	A full description of the statistical parameters including central tendency (e.g. means) or other basic estimates (e.g. regression coefficient) AND variation (e.g. standard deviation) or associated estimates of uncertainty (e.g. confidence intervals)
☑	☐	For null hypothesis testing, the test statistic (e.g. F, t, r) with confidence intervals, effect sizes, degrees of freedom and P value noted<br>*Give P values as exact values whenever suitable.*
☑	☐	For Bayesian analysis, information on the choice of priors and Markov chain Monte Carlo settings
☑	☐	For hierarchical and complex designs, identification of the appropriate level for tests and full reporting of outcomes
☑	☐	Estimates of effect sizes (e.g. Cohen's d, Pearson's r), indicating how they were calculated

Our web collection on statistics for biologists contains articles on many of the points above.

Software and code

Policy information about availability of computer code

Data collection	Retrogenix Cell Microarray Technology, MSD SECTOR Imager 2400, BD FACSymphony A3, xCELLigence RTCAeSight, Biotek Cytation 5, QX200 Droplet Reader
Data analysis	ImageQuant 8.2, BD FlowJo 9, BD FlowJo 10, GraphPad Prism 10, MSD Discovery Workbench v.4.0.13, Biotej Gen 5, QX Manager 1.2 Standard Edition, UniProt

For manuscripts utilizing custom algorithms or software that are central to the research but not yet described in published literature, software must be made available to editors and reviewers. We strongly encourage code deposition in a community repository (e.g. GitHub). See the Nature Portfolio guidelines for submitting code & software for further information.

Data

Policy information about availability of data

All manuscripts must include a data availability statement. This statement should provide the following information, where applicable:

- Accession codes, unique identifiers, or web links for publicly available datasets
- A description of any restrictions on data availability
- For clinical datasets or third party data, please ensure that the statement adheres to our policy

The source data supporting this study's findings are available in the Supplementary Data files. Source data for Figure 2 are in Supplementary Data 1. Source data for Figure 3 are in Supplementary Data 6. Source data for Figure 4 are in Supplementary Data 3. Source data for Figures 5 and 6 are in Supplementary Data 4. Source data for Figure 7 are in Supplementary Data 5. Supplementary Data 6 has the table of human plasma membrane and secreted proteins screened by Retrgoenix™ Cell Microarray Technology. Supplementary Data 7 has source data for Supplementary Figures 2B, 5, 6E, 6H, 6J, 6L, 6N, 7, 8A, 9, 10, 11, 12, and 14.

Research involving human participants, their data, or biological material

Policy information about studies with human participants or human data. See also policy information about sex, gender (identity/presentation), and sexual orientation and race, ethnicity and racism.

Reporting on sex and gender	N/A
Reporting on race, ethnicity, or other socially relevant groupings	N/A
Population characteristics	N/A
Recruitment	N/A
Ethics oversight	A statement of ethics approval for the use of human cancer cells was provided by Discovery Life Sciences.

Note that full information on the approval of the study protocol must also be provided in the manuscript.

Field-specific reporting

Please select the one below that is the best fit for your research. If you are not sure, read the appropriate sections before making your selection.

☑ Life sciences ☐ Behavioural & social sciences ☐ Ecological, evolutionary & environmental sciences

For a reference copy of the document with all sections, see nature.com/documents/nr-reporting-summary-flat.pdf

Life sciences study design

All studies must disclose on these points even when the disclosure is negative.

Sample size	In all animal studies, at least a sample size of 5 was used to detect a significant difference between groups with a signal to noise ratio of 2.0 with 80% power.
Data exclusions	In Figure 5B, illustrating the mean volume implanted in NSG mice, data were omitted for groups in which the survival rate was below 50%.
Replication	All murine experiments and in vitro experiments report pooled results from multiple biological replicates and/or multiple experiments.
Randomization	Randomization was not used for this study. Mice were evenly distributed into experimental groups from the same cohort of mice.
Blinding	Investigators were not blinded for sample processing or data analysis

Behavioural & social sciences study design

All studies must disclose on these points even when the disclosure is negative.

Study description	NA
Research sample	NA
Sampling strategy	NA
Data collection	NA
Timing	NA
Data exclusions	NA
Non-participation	NA
Randomization	NA

Ecological, evolutionary & environmental sciences study design

All studies must disclose on these points even when the disclosure is negative.

Study description	NA
Research sample	NA
Sampling strategy	NA
Data collection	NA
Timing and spatial scale	NA
Data exclusions	NA
Reproducibility	NA
Randomization	NA
Blinding	NA

Did the study involve field work? ☐ Yes ☒ No

Field work, collection and transport

Field conditions	NA
Location	NA
Access & import/export	NA
Disturbance	NA

Reporting for specific materials, systems and methods

We require information from authors about some types of materials, experimental systems and methods used in many studies. Here, indicate whether each material, system or method listed is relevant to your study. If you are not sure if a list item applies to your research, read the appropriate section before selecting a response.

Materials & experimental systems

n/a	Involved in the study	
☐	☑	Antibodies
☐	☑	Eukaryotic cell lines
☑	☐	Palaeontology and archaeology
☐	☑	Animals and other organisms
☑	☐	Clinical data
☑	☐	Dual use research of concern
☑	☐	Plants

Methods

n/a	Involved in the study	
☑	☐	ChIP-seq
☐	☑	Flow cytometry
☑	☐	MRI-based neuroimaging

Antibodies

Antibodies used	NA
Validation	The antibodies utilized were confirmed to be specific to the respective antigens through testing conducted by the manufacturers (BioLegend or BD).

Eukaryotic cell lines

Policy information about cell lines and Sex and Gender in Research

Cell line source(s)	Refer to Supplemental Table 4 and Supplemental Table 5 for the respective catalogs of all human cell lines primary normal cells used.
Authentication	The identity of the cell lines used in this manuscript was verified by the respective vendors. Additionally, cells were not passaged for more than 2 months. Additionally, cell identity was confirmed by IDEXX BioAnalytics prior to xenograft studies.
Mycoplasma contamination	All cell lines used in animal studies were confirmed negative for Mycoplasma contamination by IDEXX BioAnalytics.
Commonly misidentified lines (See ICLAC register)	N/A

Palaeontology and Archaeology

Specimen provenance	NA
Specimen deposition	NA
Dating methods	NA

☐ Tick this box to confirm that the raw and calibrated dates are available in the paper or in Supplementary Information.

Ethics oversight	NA

Note that full information on the approval of the study protocol must also be provided in the manuscript.

Animals and other research organisms

Policy information about studies involving animals; ARRIVE guidelines recommended for reporting animal research, and Sex and Gender in Research

Laboratory animals	NOD.Cg-PrkdcscidIl2rgtm1Wjl/SzJ (NSG), C57BL/6J, and CD46tg mice (B6.FVB-Tg(CD46)2Gsv/J) were obtained from The Jackson Laboratory. Hsd:Athymic Nude-Foxn1nu mice were purchased from Envigo. CD1 mice (ICR) were purchased from Charles River Laboratories. NOD-Prkdcem26Cd52Il2rgem26Cd22/NjuCrl (NCG) were purchased from Charles River Laboratories and humanized at TransCure bioServices.
Wild animals	N/A
Reporting on sex	Male and female mice were used in this manuscript. Specific details regarding the sex of mice used in each experiment are outlined in the Supplementary Methods and Figure Legends.
Field-collected samples	N/A
Ethics oversight	The respective IACUCs for IconOVir, LabCorp, Transcure, and Southern Research approved all animal studies at the respective institutions. Mice at or nearing euthanasia criteria were euthanized. There were a small number of animals in the vehicle groups that went slightly over the limit because the prior day they were not near the limit. Euthanasia criteria are described in methods.

Note that full information on the approval of the study protocol must also be provided in the manuscript.

Clinical data

Policy information about clinical studies

All manuscripts should comply with the ICMJE guidelines for publication of clinical research and a completed CONSORT checklist must be included with all submissions.

Clinical trial registration	NA
Study protocol	NA
Data collection	NA
Outcomes	NA

Dual use research of concern

Policy information about dual use research of concern

Hazards

Could the accidental, deliberate or reckless misuse of agents or technologies generated in the work, or the application of information presented in the manuscript, pose a threat to:

No | Yes

- [X] No [ ] Yes — Public health
- [X] No [ ] Yes — National security
- [X] No [ ] Yes — Crops and/or livestock
- [X] No [ ] Yes — Ecosystems
- [X] No [ ] Yes — Any other significant area

Experiments of concern

Does the work involve any of these experiments of concern:

No | Yes

- [X] No [ ] Yes — Demonstrate how to render a vaccine ineffective
- [X] No [ ] Yes — Confer resistance to therapeutically useful antibiotics or antiviral agents
- [X] No [ ] Yes — Enhance the virulence of a pathogen or render a nonpathogen virulent
- [X] No [ ] Yes — Increase transmissibility of a pathogen
- [X] No [ ] Yes — Alter the host range of a pathogen
- [X] No [ ] Yes — Enable evasion of diagnostic/detection modalities
- [X] No [ ] Yes — Enable the weaponization of a biological agent or toxin
- [X] No [ ] Yes — Any other potentially harmful combination of experiments and agents

Plants

Seed stocks: NA

Novel plant genotypes: NA

Authentication: NA

ChIP-seq

Data deposition

- [ ] Confirm that both raw and final processed data have been deposited in a public database such as GEO.
- [ ] Confirm that you have deposited or provided access to graph files (e.g. BED files) for the called peaks.

Data access links
May remain private before publication.: NA

Files in database submission: NA

Genome browser session
(e.g. UCSC): NA

Methodology

Replicates: NA

Sequencing depth: NA

Antibodies: NA

Peak calling parameters: NA

Data quality: NA

Software: NA

Flow Cytometry

Plots

Confirm that:

- [x] The axis labels state the marker and fluorochrome used (e.g. CD4-FITC).
- [x] The axis scales are clearly visible. Include numbers along axes only for bottom left plot of group (a 'group' is an analysis of identical markers).
- [x] All plots are contour plots with outliers or pseudocolor plots.
- [x] A numerical value for number of cells or percentage (with statistics) is provided.

Methodology

Sample preparation	See Materials and Methods and Supplementary Methods.
Instrument	BD FACSymphony A3 were used for data collection.
Software	BD FlowJo 9 and BD FlowJo 10 were used to analyse fcs files.
Cell population abundance	At least 100,000 cells were acquired on the flow cytometer to determine cell population abundance.
Gating strategy	Gating strategies are described within the manuscript Materials and Methods and Figures.

- [x] Tick this box to confirm that a figure exemplifying the gating strategy is provided in the Supplementary Information.

Magnetic resonance imaging

Experimental design

Design type	NA
Design specifications	NA
Behavioral performance measures	NA

Imaging type(s)	NA
Field strength	NA
Sequence & imaging parameters	NA
Area of acquisition	NA

Diffusion MRI ☐ Used ☑ Not used

Preprocessing

Preprocessing software	NA
Normalization	NA
Normalization template	NA
Noise and artifact removal	NA
Volume censoring	NA

Statistical modeling & inference

Model type and settings	NA
Effect(s) tested	NA

Specify type of analysis: ☐ Whole brain ☐ ROI-based ☐ Both

Statistic type for inference<br>(See Eklund et al. 2016)	NA
Correction	NA

Models & analysis

n/a	Involved in the study
X	☐ Functional and/or effective connectivity
X	☐ Graph analysis
X	☐ Multivariate modeling or predictive analysis

Functional and/or effective connectivity	NA
Graph analysis	NA
Multivariate modeling and predictive analysis	NA

This checklist template is licensed under a Creative Commons Attribution 4.0 International License, which permits use, sharing, adaptation, distribution and reproduction in any medium or format, as long as you give appropriate credit to the original author(s) and the source, provide a link to the Creative Commons license, and indicate if changes were made. The images or other third party material in this article are included in the article's Creative Commons license, unless indicated otherwise in a credit line to the material. If material is not included in the article's Creative Commons license and your intended use is not permitted by statutory regulation or exceeds the permitted use, you will need to obtain permission directly from the copyright holder. To view a copy of this license, visit http://creativecommons.org/licenses/by/4.0/
